# Changes in electrophysiological aperiodic activity during cognitive control in Parkinson’s disease

**DOI:** 10.1093/braincomms/fcae306

**Published:** 2024-09-07

**Authors:** Noémie Monchy, Julien Modolo, Jean-François Houvenaghel, Bradley Voytek, Joan Duprez

**Affiliations:** LTSI-U1099, University of Rennes, Rennes F-35000, France; LTSI-U1099, University of Rennes, Rennes F-35000, France; LTSI-U1099, University of Rennes, Rennes F-35000, France; Department of Neurology, Rennes University Hospital, Rennes 35033, France; Department of Cognitive Science, Halıcıoğlu Data Science Institute, University of California, San Diego, La Jolla, CA, USA; LTSI-U1099, University of Rennes, Rennes F-35000, France

**Keywords:** EEG, 1/f, Parkinson’s disease, cognitive control, Simon task

## Abstract

Cognitive symptoms in Parkinson’s disease are common and can significantly affect patients’ quality of life. Therefore, there is an urgent clinical need to identify a signature derived from behavioural and/or neuroimaging indicators that could predict which patients are at increased risk for early and rapid cognitive decline. Recently, converging evidence identified that aperiodic activity of the EEG reflects meaningful physiological information associated with age, development, cognitive and perceptual states or pathologies. In this study, we aimed to investigate aperiodic activity in Parkinson’s disease during cognitive control and characterize its possible association with behaviour. Here, we recorded high-density EEG in 30 healthy controls and 30 Parkinson’s disease patients during a Simon task. We analysed task-related behavioural data in the context of the activation–suppression model and extracted aperiodic parameters (offset, exponent) at both scalp and source levels. Our results showed lower behavioural performances in cognitive control as well as higher offsets in patients in the parieto-occipital areas, suggesting increased excitability in Parkinson’s disease. A small congruence effect on aperiodic parameters in pre- and post-central brain areas was also found, possibly associated with task execution. Significant differences in aperiodic parameters between the resting-state, pre- and post-stimulus phases were seen across the whole brain, which confirmed that the observed changes in aperiodic activity are linked to task execution. No correlation was found between aperiodic activity and behaviour or clinical features. Our findings provide evidence that EEG aperiodic activity in Parkinson’s disease is characterized by greater offsets, and that aperiodic parameters differ depending on arousal state. However, our results do not support the hypothesis that the behaviour-related differences observed in Parkinson’s disease are related to aperiodic changes. Overall, this study highlights the importance of considering aperiodic activity contributions in brain disorders and further investigating the relationship between aperiodic activity and behaviour.

## Teaser

Monchy *et al*. investigated EEG aperiodic activity in Parkinson’s disease during cognitive control. As compared to controls, patients had greater offsets in parieto-occipital regions, suggesting increased excitability. Significant differences in aperiodic parameters between resting-state, pre- and post-stimulus phases provide evidence that aperiodic activity differs depending on arousal state.

## Graphical Abstract

**Graphical Abstract:**
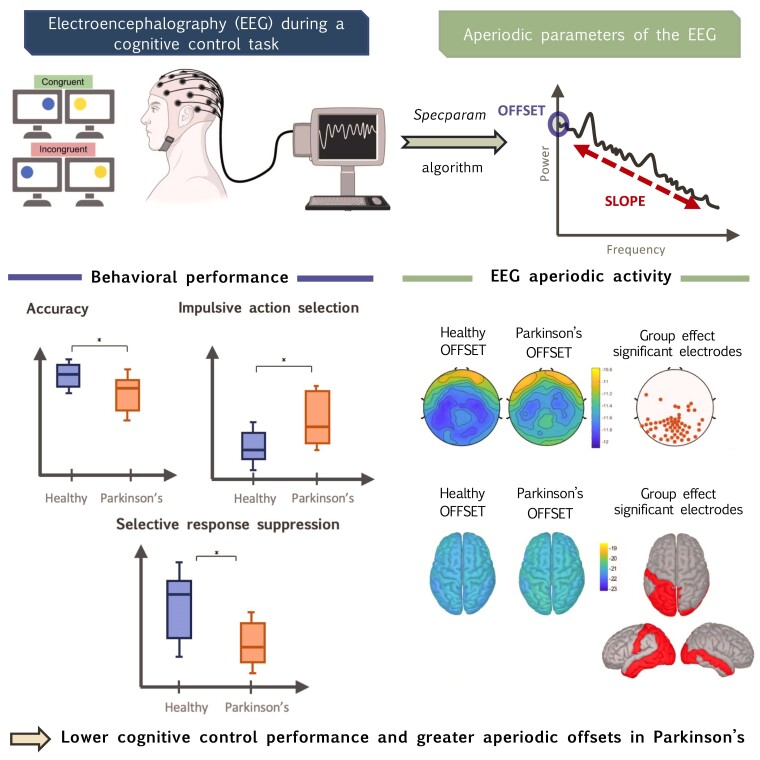

## Introduction

Parkinson’s disease is a multisystem disorder characterized by motor (bradykinesia, resting tremor, postural instability and rigidity) and non-motor symptoms such as cognitive deficits.^1^ Cognitive symptoms can have a major impact on patients’ quality of life^2^ and may precede the onset of motor features.^3^ These alterations are quite heterogeneous among patients in their progression and severity.^4,5^ In Parkinson’s disease, and other neurodegenerative diseases as well, cognitive symptoms remain hard to alleviate; although some available treatments showed positive effects,^6-8^ some treatments can even worsen them.^9-11^ Unfortunately, tools to predict cognitive deterioration, or the influence of therapies on cognition, are still lacking, highlighting the need to incorporate new techniques. Therefore, there is a strong clinical need in identifying a signature derived from several behavioural and/or neuroimaging indicators, which could ultimately predict which patients are at increased risk of early and rapid cognitive decline.

One of the most common cognitive impairments in Parkinson’s disease is the alteration of efficient and rapid adaptation to environmental changes.^12-14^ Specifically, Parkinson’s disease patients exhibit disorders in cognitive action control (CAC), a subprocess of cognitive control that suppresses automatic responses in favour of voluntary goal-directed actions.^15^ CAC is classically studied using conflict tasks, such as the Simon task,^16^ in which participants have to respond according to the stimulus colour while ignoring its location. Automatic processing of the stimulus’ spatial location can induce conflict (incongruent trials) that increases reaction times (RTs) and decreases accuracy, reflecting the so-called congruence effect.^17-19^ Most studies focusing on the effect of Parkinson’s disease on CAC performances demonstrated a higher congruence effect on RT or accuracy in Parkinson’s disease patients,^14,20-22^ although others did not replicate this result.^23,24^ To understand this divergence, some authors have used the activation–suppression model, which investigated the dynamics of CAC using distributional analyses of the congruence effect. These studies showed that Parkinson’s disease patients tend to react automatically (greater impulsive action selection) and have difficulty suppressing the automatic activation, even when they have time to do so (impaired selective response suppression).^25-29^

CAC is mostly supported by a prefrontal–basal ganglia network where the pre-supplementary motor area, the dorsolateral prefrontal cortex, the inferior frontal cortex and the subthalamic nucleus all play a major role in impulsive action selection and suppression.^30^ In Parkinson’s disease, cognitive impairments could be explained by the accumulation of alpha-synuclein, which leads to synaptic and axonal dysfunctions.^31^ This would impact the integrity and efficiency of synaptic communication, undoubtedly essential for cognitive processes requiring fast decision making such as CAC.

The study of cognitive processes such as CAC is usually carried out using EEG with an excellent temporal resolution particularly adapted to dynamic processes. It is also arguably a more direct measure of neural activity than other neuroimaging modalities, especially regarding the measurement of neural oscillations that have been linked to behaviour and notably with CAC.^32^ For instance, Singh *et al*.^33^ investigated mid-frontal theta (4–8 Hz) in Parkinson’s disease patients during a Simon task. Results suggested that patients had an overall attenuated mid-frontal theta activity, but which was not specifically associated with changes in response conflict. This neural measure was correlated with cognitive dysfunction, supporting that cognitive failures in Parkinson’s disease are related to attenuated cortical cognitive control mechanisms.^34^

One difficulty in analysing electrophysiological signals is that they typically exhibit both *periodic* and *aperiodic* properties, and these signal features are conflated.^35,36^ The periodic component, commonly referred to as neural oscillations, has received considerable attention following historical traditions, whereas the aperiodic part has only recently gained more interest and was commonly discarded or not considered. While oscillatory power is concentrated at specific frequencies, which is visible as peaks on the power spectrum,^37^ neural power spectra also exhibit a broadband 1/f^χ^ scaling, where power decreases exponentially as a function of frequency.^38,39^ This ‘1/f-like’ characteristic can be described by two parameters: *aperiodic offset* corresponding to the broadband offset of the spectrum and *aperiodic exponent* defined as the χ in ‘1/f^χ^’ describing the overall decreasing slope of power across frequencies.^35^ Importantly, the generative mechanisms of aperiodic activity have not been firmly established to date. Among the possible mechanisms, animal and computational models have suggested that the aperiodic exponent could be a reflection of the ‘excitation–inhibition’ balance (the so-called ‘E:I ratio’) in cortical circuits.^40,41^ Recently, converging evidence points at the conclusion that changes in the aperiodic component are associated with cognitive^35,42^ and perceptual states,^43^ development,^44^ aging^45^ and pathological conditions.^46-52^

Thus, ignoring the aperiodic component can lead to a misrepresentation and misinterpretation of physiological mechanisms.^35,44^ Furthermore, the use of *a priori* frequency bands for oscillatory analyses can result in misinterpreting aperiodic activity as periodic activity, and thus bias the power of actual physiological oscillations, or even lead to extracting power from oscillations that simply do not exist in some cases.^53^ Therefore, there is an increasing consensus on the imperative careful parametrization of spectral features to minimize its associated bias.^35^

Since changes in aperiodic component are associated with cognitive state and Parkinson’s disease patients exhibit cognitive alterations, we argue that studying aperiodic activity in Parkinson’s disease patients could provide further insights regarding the pathophysiology of cognitive decline and the design of electrophysiology-based biomarkers. To date, only a few studies have focused on aperiodic parameters in Parkinson’s disease patients. Four studies have investigated local field potential signals in the subthalamic nucleus and evidenced a lower exponent during movements versus rest, which correlated with motor symptom severity,^54,55^ and confirmed that aperiodic exponent can predict the motor response to subthalamic nucleus deep brain stimulation (STN-DBS).^55^ Regarding STN-DBS, a systematic increase was also found in both exponent and offset of the aperiodic spectrum 18-month follow-up after surgery in Parkinson’s disease patients.^56^ More recently, Wiest *et al.*^41^ evidenced that the aperiodic exponent of subthalamic field potentials reflects E:I ratio in Parkinson’s disease. Five other studies were based on non-invasive scalp EEG recordings. Recent studies evidenced that Parkinson’s disease is associated with significant increase in aperiodic activity during resting state^57-59^ while a decreased aperiodic activity in patients has been observed by Rosenblum *et al.*^60^ More specifically, Wang *et al.*^61^ found that aperiodic parameters were increased in Parkinson’s disease patients after dopaminergic medication, especially in bilateral central brain regions, while McKeown *et al*.^59^ found that the increase in aperiodic activity in Parkinson’s disease was independent of medication status.

To the best of our knowledge, it has still not been investigated to what extent aperiodic components are associated with behavioural alterations in Parkinson’s disease. In this study, we focused on aperiodic activity in Parkinson’s disease during cognitive control and tested the hypothesis that aperiodic EEG activity is related to the CAC alterations observed in Parkinson’s disease. According to the literature, we expected to identify (i) behavioural alterations (higher congruence effect, increased impulsive action selection, decreased selective response suppression) and (ii) different aperiodic parameters between Parkinson’s disease and healthy control (HC), since Parkinson’s disease is associated with changes in cortical E:I ratio.^62^

## Materials and methods

### Participants

Thirty HCs (15 males, 15 females) aged between 45 and 70 years (mean = 61.7, SD = 7.3) and 30 patients (15 males, 15 females) diagnosed with idiopathic Parkinson’s disease,^63^ aged between 45 and 73 years (mean = 60.4, SD = 7.3) were enrolled in this study. One patient was excluded due to a technical acquisition issue during an experimental session. Thus, all subsequent analyses were performed on 30 HC and 29 Parkinson’s disease patients (Table 1). HC and patients did not significantly differ in age, sex or education.

**Table 1:** Demographic characteristic of participants

	Healthy controls (*n* = 30)	Parkinson’s disease patients (*n* = 29)	*t*-test
**Age (years)**	61.7 (7.3)	60.4 (7.3)	*t* = 0.77; *P* = 0.44
**Sex (M:F)**	15:15	14:15	
**Education (years)**	13.5 (3.6)	12.5 (3.2)	*t* = 1.19; *P* = 0.24
**MoCA (/30)**	28 (1.9)	26.6 (2.0)	*t* = 2.72; *P* < 0.01
**Disease duration (years)**		12.1 (4.2)	
**Side of onset (L:R)**		16:13	
**LEDD (mg/day)**		1348.0 (462.0)	
**UPDRS III-ON**		10.0 (5.7)	
**UPDRS III-OFF**		36.8 (12.5)	
**Hoehn & Yarh-ON**		1.1 (0.8)	
**Hoehn & Yarh-OFF**		2.0 (0.4)	
**Schwabb & England-ON**		93.0 (5.6)	
**Schwabb & England-OFF**		76.5 (10.3)	

Data are presented as mean (standard deviation). Unpaired *t*-tests were used to assess differences between the two study populations.

MoCA, Montreal Cognitive Assessment Scale; LEDD, levodopa equivalent daily dose; UPDRS, Unified Parkinson’s Disease Rating Scale; M, male; F, female; L, left; R, right.

All patients were recruited from the Neurology Department of Rennes University Hospital (France) during a 1-week hospitalization, as part of their usual clinical care. During that week, clinical examination included an OFF-medication period but patients were ON dopaminergic medication, measured by levodopa equivalent daily dose (LEDD), for all parts of the experiment (Table 1). Patients with DBS devices were not included. All HCs were recruited from the general population by advertising. Participants had normal or corrected-to-normal vision.

All participants underwent a neuropsychological assessment that revealed no major cognitive impairment [all Montreal Cognitive Assessment Scale (MoCA) scores >22]^64^ nor severe neurocognitive disorder according to the Diagnostic and Statistical Manual of Mental Disorders-V. We limited ourselves to using the recommended MoCA thresholds to exclude patients with Parkinson’s disease dementia, mostly in order to limit patients’ fatigue. We did respect the Level 1 criteria to include patients who, in the worst-case scenario, have mild cognitive impairment. All participants were also free from moderate or severe psychiatric symptoms and present or past neurological pathology (other than Parkinson’s disease for patients). The severity of patients’ motor symptoms was assessed using the Unified Parkinson’s Disease Rating Scale (UPDRS) III scale^65^ (Table 1).

This study was conducted in accordance with the Declaration of Helsinki and was approved by a national ethics committee (CPP ID-RCB: 2019-A00608-49; approval number: 19.03.08.63626). After a complete description of the study, all participants gave their informed written consent.

### Task design and procedure

Participants were asked to perform a colour version of the Simon task^16^ to assess CAC. They were asked to press a right or left button, as quickly and accurately as possible, according to the colour of the circle while ignoring its right or left location on the computer screen (colour/side mapping was counterbalanced across participants). Participants had to respond within 1000 ms after stimulus offset. In congruent trials, the side of presentation of the circle matched with the side of the button press associated with the colour, facilitating the response of the subject. In incongruent trials, colour and location did not match and activated conflicting responses (Supplementary Fig. 1). Participants first performed a training session composed of 60 trials and then an experimental phase organized in 10 blocks of 60 trials. Each block contained 30 congruent and 30 incongruent randomized trials. In total, 600 trials were performed with 300 congruent and 300 incongruent trials. For further details, see Duprez *et al*.^66^

### High-density EEG recording and processing

#### Recording

EEG data were recorded using a high-density EEG (HD-EEG) system (EGI, Electrical Geodesic Inc., 256 channels) with a sampling frequency of 1000 Hz, an electrode impedance maintained below 25 kΩ, the Cz electrode used as the reference and a ground close to Pz. Electrodes on the net were placed using the standard 10-10 geodesic montage. Most jaw and neck electrodes were removed due to excessive muscular artefacts, resulting in a total of 199 exploitable electrodes. The EEG recording consisted in a 5-min resting-state period with eyes closed followed by the training.

#### Pre-processing

All EEG pre-processing and subsequent analyses were performed manually using the Brainstorm toolbox^67^ in MATLAB (MathWorks^Ⓡ^, USA). First, direct current (DC) offset removal was applied (removed the mean of the entire recorded signal). Second, a notch filter (50 Hz) and a band-pass filter (1–90 Hz) were applied (finite impulse response; FIR filter using Kaiser window). Third, signals were visually inspected and bad channels were removed before being interpolated using Brainstorm’s default parameters. Fourth, independent component analysis (*jade* method) was used to remove eye blinks and muscle artefacts following visual inspection of the independent components. Fifth, signals were segmented into 4-s epochs for the resting-state period and from −700 to 1200 ms relative to the stimulus onset during the task. Finally, a visual inspection was performed to manually reject remaining bad epochs. As a result, an average of 59 (SD = 14) resting-state epochs and 326 (SD = 13) correct trials were studied per subject, corresponding at 80% (SD = 18%) resting-state epochs per subject and 55% (SD = 11%) correct trials per subject. Only correct congruent and incongruent trials were studied, since they are associated with efficient control.

**Figure 1:**
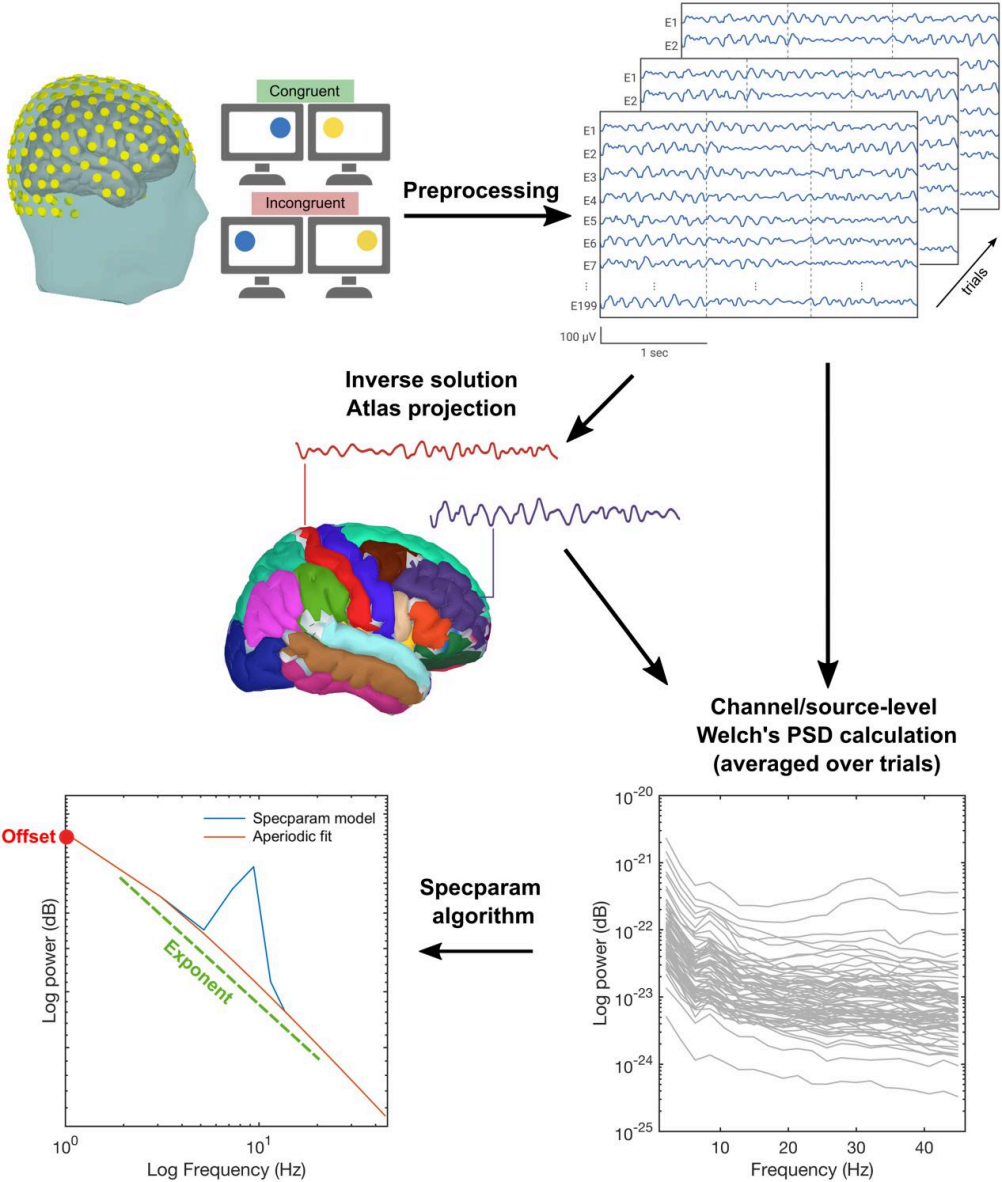
**Overview of the pipeline analysis of HD-EEG data.** First, HD-EEG data were recorded during the Simon task (only correct trials were considered for the post-stimulus part). After pre-processing, PSD was estimated on the resting-state period, on the pre-stimulus part (−500 to 0 ms) and on the post-stimulus part (0–1000 ms) for each epoch. The spectra obtained were averaged by subject and condition for the post-stimulus part and only by subject for resting state and pre-stimulus period. Finally, we applied the *specparam* algorithm to extract the aperiodic parameters for each averaged spectrum.

After pre-processing, several steps were applied to extract aperiodic parameters at both scalp and source levels, as summarized in Fig. 1 and explained below.

#### Source reconstruction

A realistic head model was used along with the position of the electrodes. In this study, we used the boundary element method head model fitted to the International Consortium for Brain Mapping Magnetic Resonance Imaging template^68^ and the OpenMEEG toolbox.^69^ The EEG inverse problem was solved with the weighted minimum norm estimate (wMNE) method.^70^ We used the Desikan–Killiany atlas parcellation [68 regions of interest (ROIs)]^71^ to project the cortical sources and constrained their orientation normally to the cortical surface.^72^ We used a noise covariance matrix computed from our 700 ms pre-stimulus baseline and set the signal-to-noise ratio and the depth weighting to default values.

### Parameterization of the spectral data

Power spectrum density (PSD) was obtained for each epoch using Welch’s method (window length of 0.5 s and window overlap ratio of 50%), which is a necessary pre-requisite for the subsequent estimation of aperiodic parameters. PSD was calculated on the resting-state period, on the pre-stimulus part, i.e. from −500 to 0 ms before the stimulus appeared on the screen, and on the post-stimulus part, i.e. from 0 to 1000 ms after the stimulus appeared on the screen. The obtained spectra were averaged by subject and condition for the post-stimulus part and by subject only for the resting state and pre-stimulus part. For each resulting power spectrum, we applied the spectral parameterization algorithm across the 1–40 Hz frequency band (*specparam* toolbox^73^ version 1.0), which considers the PSD as a linear combination of two different types of components: aperiodic and periodic (oscillatory) components.^35^ The aperiodic component consisted of the exponent (overall spectrum slope) and offset (intercept of the curve) parameters. In order to optimize parameters of the *specparam* algorithm to our data, we compared the goodness-of-fit metrics of several *specparam* models and selected the model with the best fitting metrics (channel-averaged *R*^2^ = 0.94; channel-averaged mean squared error = 4.5^−3^). The optimal parameters were as follows: peak width limits: [0.5–6]; maximum number of peaks: 4; minimum peak height: 1.0; peak threshold: 2.0; and aperiodic mode = ‘fixed’. Aperiodic parameters were estimated at both the scalp and the cortical ROI levels.

### Statistical analysis

All statistical analyses were conducted in R v.4.1.3^74^ implemented with the *tidyverse* and *lme4* package.^75,76^ For all analyses, we chose the standard significance threshold of *P* = 0.05.

#### Behavioural responses

For each participant and for each trial, RT and accuracy scores were extracted. To estimate the congruence effect, we compared congruent and incongruent RT of correct responses as well as accuracy. Then, we also compared these data between groups to estimate the impact of Parkinson’s disease. In light of the activation–suppression model,^77^ we further analysed behavioural data with distributional analyses.^29,78^ First, impulsive action selection (incongruent accuracy of the fastest trials) was investigated using conditional accuracy functions (CAFs) displaying accuracy rate against the RT distribution for each condition and group. For each participant, RTs were rank ordered and split into seven bins containing an equal number of trials, and mean accuracy was then plotted for each bin. The activation–suppression model postulates that incongruent accuracy of the first bin (fastest trials) informs about impulsive action selection. Second, we used delta plots to assess the dynamics of selective response suppression (slope value of the congruence effect for the slowest trials). Delta plots display the mean congruence effect (incongruent RT–congruent RT) as a function of the RT distribution of correct responses, splitted into seven bins, as we did for CAFs. The activation–suppression model postulates that the slope between the two last bins of delta plots informs about the selective response suppression.

The effect of congruence and group on RT and accuracy were analysed using linear and non-linear mixed models, respectively. These models are composed of two fixed factors: group and congruence, and a random effect of condition by subject was added. Overall, 551 (SD = 81) correct trials on average per subject were kept for behavioural analyses due to the removal of errors, absence of response within 1000 ms or remaining artefacts. RTs were log-transformed for increased compliance with the model’s assumptions (homogeneity of variance and normal distribution of residuals model).

Fixed effects significance was computed through the Anova function of the {*car*} package^79^ that calculates type II Wald *χ*^2^ tests. Marginal (mR^2^) and conditional (cR^2^) were calculated using the {MuMin} package.^80^ These models offered the possibility to exploit the whole data set, avoiding the loss of statistical power due to averaging data, and also enabled taking into account inter-individual variability and unbalanced data.^81^

Group effects on the average first bin of CAF and on the last slope values of delta plots were estimated with Welch’s *t*-test, since only one averaged value exists for each subject, and are reported with effect size (Cohen’s *d*).

#### EEG parameters

Following the extraction of aperiodic parameters, we first performed analyses on the post-stimulus part (0–1000 ms after the stimulus onset) on both scalp and cortex levels. Within each level, we studied the aperiodic parameters averaged over all the electrodes/ROIs and for each electrode/ROI. Group and congruence effects on aperiodic offset and exponent were assessed using two-way repeated measures ANOVAs where the between-subject factor variable was the group and the within-subject factor variable was the congruence. Classical ANOVAs were chosen over mixed models here because mixed modeling only converges when several repetitions by factor combination are available, which was not the case of the aperiodic parameters as compared to behavioural data. This is because there was only one average value of aperiodic offset or exponent per factor combination, while dozens of trials with corresponding RT and accuracy were available per factor combination. Assumptions were checked for each test (outliers and normality assumption). Paired Student’s *t*-tests were performed as *post hoc* tests where the effect was significant. False discovery rate (FDR) correction was applied to all *post hoc* tests.

Then, we analysed group and period effects (resting state versus pre-stimulus period versus post-stimulus period) on aperiodic parameters in order to verify if aperiodic parameters were different when participants were performing the task compared to resting-state and pre-stimulus periods. We applied the same pipeline analysis as described above except that the within-subject factor variable of the two-way repeated measures ANOVAs was the period (resting-state, pre-stimulus or post-stimulus part).

The onset side of the disease was also assessed on both scalp and cortex levels via two-way repeated measures ANOVAs where the between-subject factor variable was the side of onset and the within-subject factor variable was the congruence.

#### Correlations between behaviour and EEG parameters

Last, we tested whether behavioural variations were associated with aperiodic parameters. To achieve this, we used Spearman correlations between behavioural responses as dependent variables (RT, accuracy, first bin of CAF, last slope of delta plots) and EEG parameters from *specparam* as independent variables (offset, exponent). We then applied a FDR correction to account for multiple comparisons tests. We also tested correlations between aperiodic parameters and clinical characteristics of the population (age, MoCA, disease duration, UPDRS III ON/OFF scores, LEDD).

## Results

### Behavioural analysis

**Figure 2:**
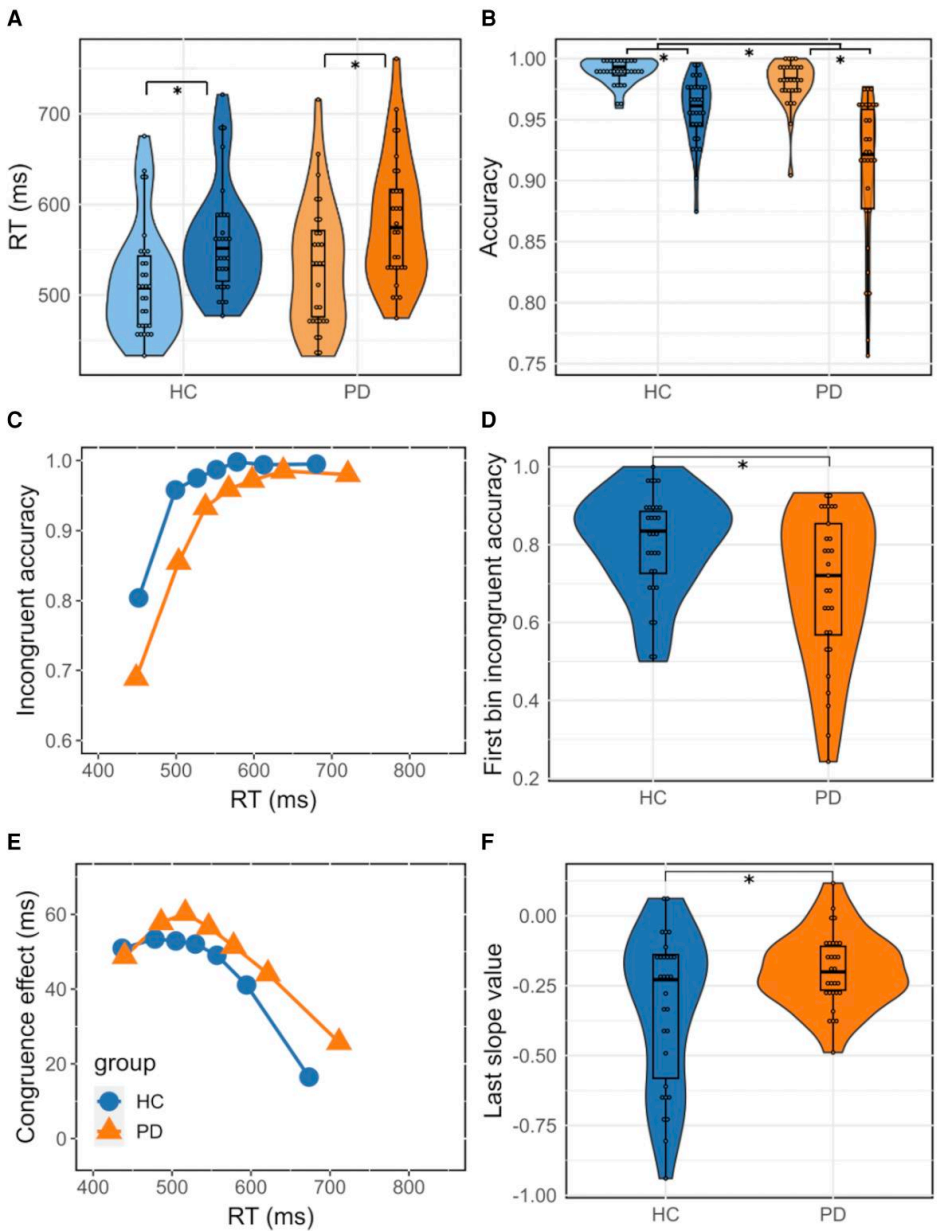
**Subject-averaged RT (A) and accuracy (B) as a function of congruence in both groups.** The lightest colour corresponds to congruent trial values, while the darkest one corresponds to incongruent trial values. The effects of congruence and group on RT were analysed using a linear mixed model (congruence effect: *χ*^2^ = 451.9, *P* < 0.0001; mR^2^ = 0.06; cR^2^ = 0.42; group effect: *χ*^2^ = 1.3, *P* = 0.25), and a non-linear mixed model assessed the effect of congruence and group on accuracy (congruence effect: *χ*^2^ = 163.1, *P* < 0.0001; mR^2^ = 0.18; cR^2^ = 0.3; group effect: *χ*^2^ = 18.1, *P* < 0.0001). CAFs for the incongruent situation (**C**) are plotted against RT distribution as a function of group. Impulsive action selection is denoted by violin plots showing accuracy of the first incongruent bin (**D**) by group. Group effect on the average first bin of CAF was evaluated with Welch’s *t*-test (*t* = 2.67, *P* = 0.01; Cohen’s *d* = 0.70). Delta plots showing changes in the congruence effect as a function of RT distribution in both groups (**E**). The strength of selective inhibition is represented by violin plots showing the value of the last slope of the delta plots in both groups (**F**). Group effect on the last slope values of delta plots was estimated with Welch’s *t*-test (*t* = −2.44, *P* = 0.02; Cohen’s *d* = −0.63). *P < 0.05. HC, healthy control.

The congruence effect classically reported in the literature was observed, as indicated by longer RTs (*χ*^2^ = 451.9, *P* < 0.0001; mR^2^ = 0.06; cR^2^ = 0.42) and more errors (*χ*^2^ = 163.1, *P* < 0.0001; mR^2^ = 0.18; cR^2^ = 0.3) in the incongruent versus congruent situation (Fig. 2A and B). Thus, participants were slower and made more errors overall during conflict. Parkinson’s disease patients were less accurate than HCs regardless of congruence (*χ*^2^ = 18.1, *P* < 0.0001), but had a similar RT (*χ*^2^ = 1.3, *P* = 0.25). The congruence effect was not significantly different between groups, both for RT (*χ*^2^ = 0.25, *P* = 0.62) and accuracy (*χ*^2^ = 0.23, *P* = 0.63). This suggests that conflict resolution, based only on the analyses of overall congruence effect, was not different between the two groups.

CAFs revealed the typical pattern of lower accuracy for the fastest RTs (Fig. 2C). Accuracy of the first bin (the fastest responses) in the incongruent condition (Fig. 2D) was significantly lower in Parkinson’s disease patients in comparison with HCs (*t* = 2.67, *P* = 0.01; Cohen’s *d* = 0.70), reflecting a higher impulsive action selection in the patient group.

Delta plots also displayed the typical decreasing pattern of the congruence effect with RT associated with the Simon task (Fig. 2E). Parkinson’s disease patients had a significantly flatter slope, indicating reduced ability in inhibiting automatic responses (Fig. 2F; *t* = −2.44, *P* = 0.02; Cohen’s *d* = −0.63).

### EEG analysis

#### Onset side on aperiodic parameters

We first tested whether the averaged exponent and offset values across electrodes and ROIs differ according to the onset side in Parkinson’s disease patients. At the scalp level, the averaged aperiodic parameters did not differ according to the affected side [exponent: *F*(1,27) = 0.33; *P* = 0.569; offset: *F*(1,27) = 0.329; *P* = 0.57], as well as at the cortex level [exponent: *F*(1,27) = 0.12; *P* = 0.74; offset: *F*(1,27) = 0.86; *P* = 0.36].

Then, we focused on the distribution of aperiodic parameters for all electrodes and ROIs. At both levels, we found no significant differences in exponent distribution between patients with left alteration and those that had an alteration on the right side (see Supplementary Table 11). Regarding offset distribution, no significant difference has been evidenced between those patients (see Supplementary Table 11).

#### Group and congruence effects on aperiodic parameters

##### Scalp level

**Figure 3:**
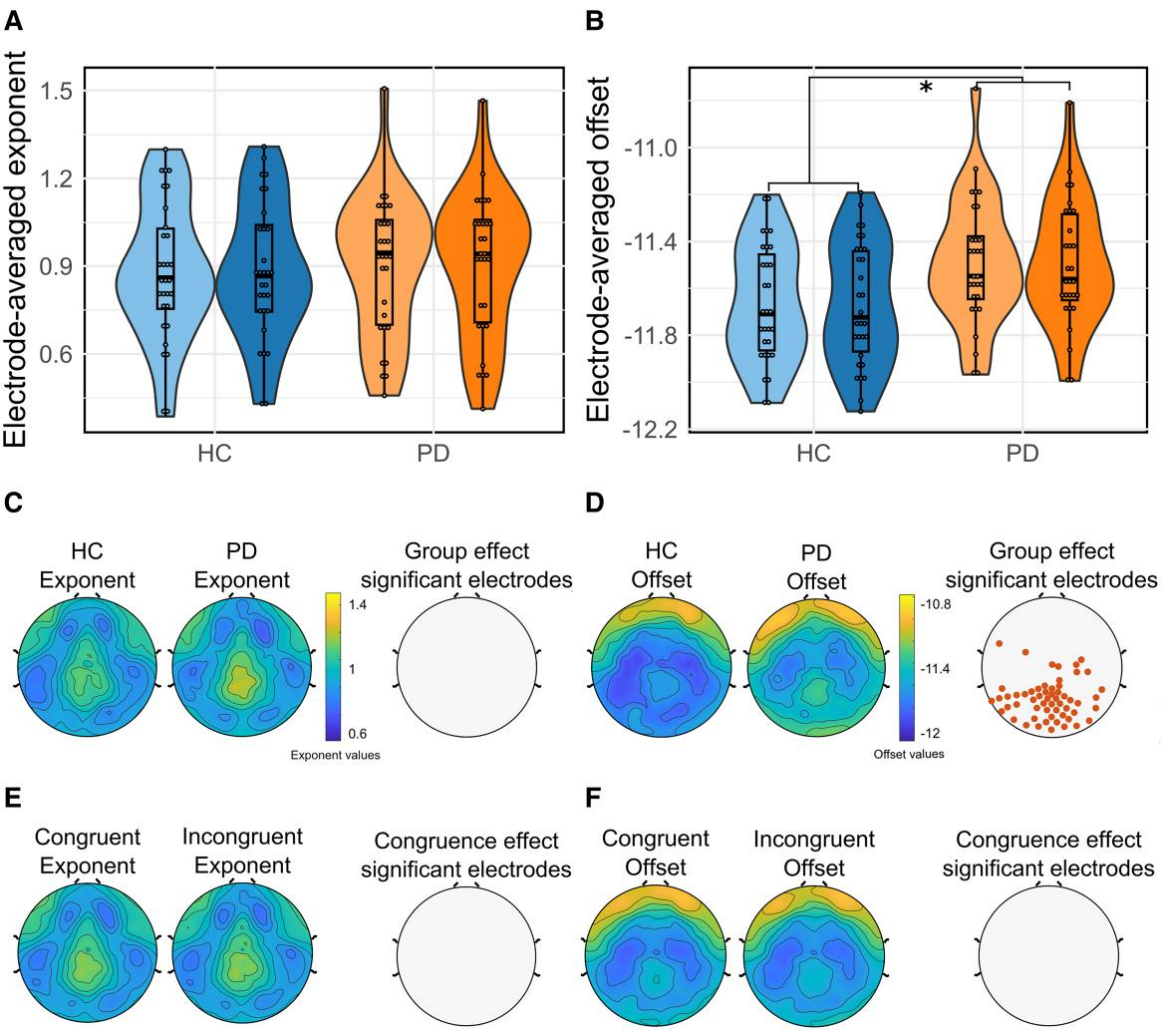
**Violin plots showing the distribution of electrode-averaged exponent (A) and offset (B) values by condition and for each group.** The lightest colour corresponds to congruent trial values, while the darkest one corresponds to incongruent trial values. Congruence and group effects on electrode-averaged aperiodic exponent were assessed using a two-way repeated measures ANOVA [congruence effect: *F*(1,57) = 0.23; *P* = 0.63; group effect: *F*(1,57) = 0.091; *P* = 0.76]. Congruence and group effects on electrode-averaged aperiodic offset were also assessed using a two-way repeated measures ANOVA [congruence effect: *F*(1,57) = 0.044; *P* = 0.834; group effect: *F*(1,57) = 6.36; *P* = 0.014]. Spatial topographies of the aperiodic exponent (**C**) and offset (**D**) values across the scalp regardless congruence for HC and Parkinson’s disease patients. Comparisons between groups were performed with ANOVAs for each electrode (FDR corrected). Electrodes with significant differences in aperiodic parameters between HC and Parkinson’s disease patients are represented with dots. For exact test statistic values, see Supplementary Table 1. Spatial topographies of the aperiodic exponent (**E**) and the offset (**F**) values across the scalp regardless of the group. Comparisons between conditions were performed with ANOVAs for each electrode (FDR corrected). Electrodes with significant differences in aperiodic parameters between condition are represented with red dots. For exact test statistic values, see Supplementary Table 1. HC, healthy control. * indicates *P* < 0.05.

We first took a global approach, averaging the exponent and offset values over all the electrodes for each subject (Fig. 3A and B). The electrode-averaged exponent and offset did not differ according to congruence [exponent: *F*(1,57) = 0.23; *P* = 0.63; offset: *F*(1,57) = 0.044; *P* = 0.834]. However, offset values significantly differed between groups: Parkinson’s disease had higher aperiodic offsets [*F*(1,57) = 6.36; *P* = 0.014] than HC, while electrode-averaged exponent values did not differ between groups [*F*(1,57) = 0.091; *P* = 0.76].

Then, we analysed the distribution of aperiodic parameters for all electrodes across the scalp for each group regardless of condition (Fig. 3C and D). We observed that the distribution pattern of the offset and exponent values seemed similar between HC and Parkinson’s disease patients. Regarding exponent distribution, no significant differences in values have been shown between groups (Fig. 3C). Aperiodic offsets in Parkinson’s disease were significantly higher as compared to HC (Fig. 3D) over a large majority of parieto-occipital electrodes (Fig. 3D). For further details, see Supplementary Table 1.

Distribution of aperiodic parameters across the scalp was also estimated for each condition regardless of group (Fig. 3E and F). We found no significant differences in aperiodic offset and exponent between conditions. For further details, see Supplementary Table 1.

##### Source level

The ROI-averaged exponent and offset did not differ according to congruence [exponent: *F*(1,57) = 0.065; *P* = 0.8; offset: *F*(1,57) = 0.101; *P* = 0.752]. However, ROI-averaged offset values significantly differed between groups: Parkinson’s disease had higher aperiodic offsets [*F*(1,57) = 6.17; *P* = 0.016] than HC, while ROI-averaged exponent values did not differ between groups [*F*(1,57) = 0.705; *P* = 0.405].

**Figure 4:**
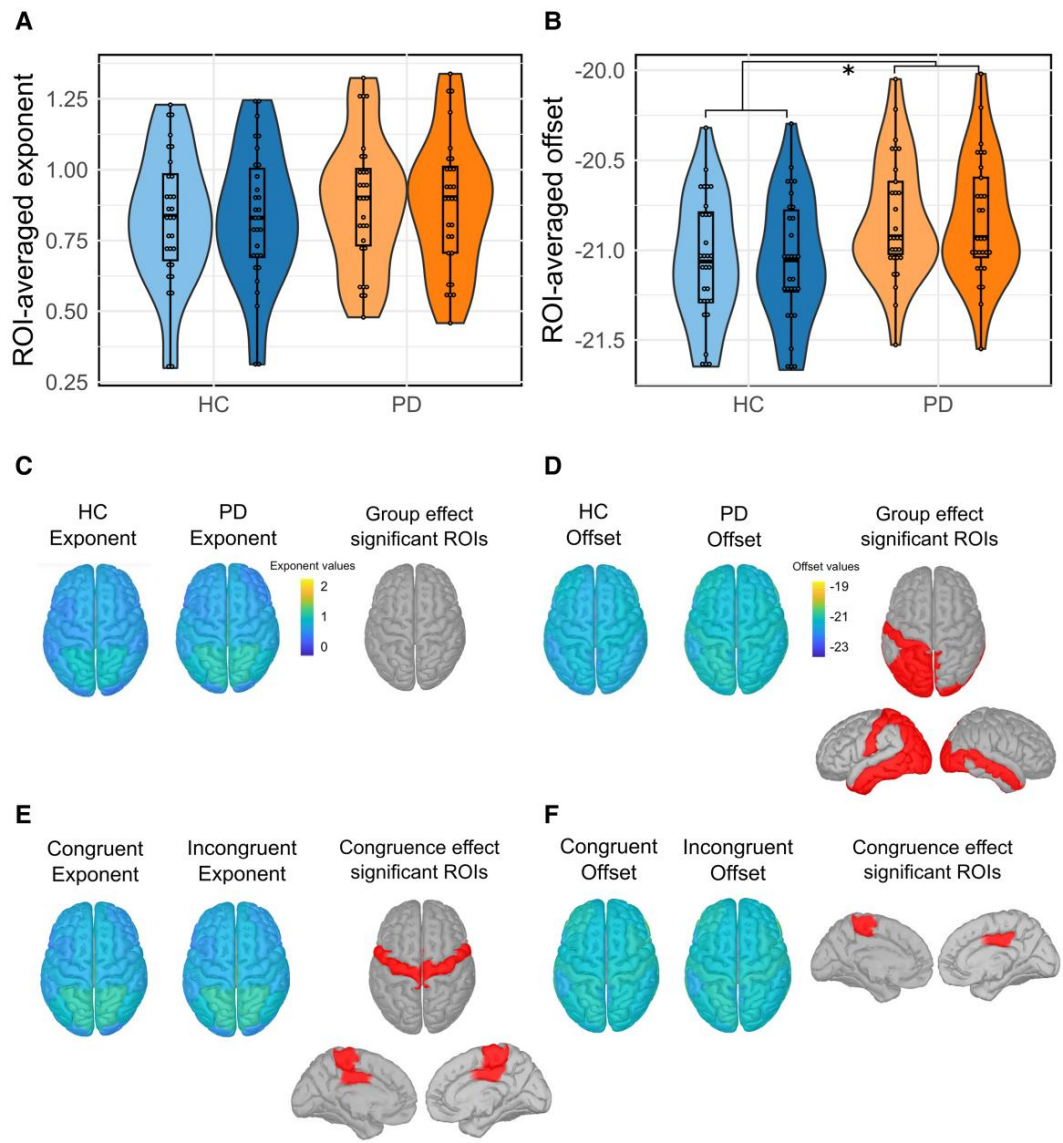
**Violin plots showing the distribution of ROI-averaged exponent (A) and offset (B) values by condition and for each group.** The lightest colour corresponds to congruent trial values, while the darkest one corresponds to incongruent trial values. Congruence and group effects on ROI-averaged aperiodic exponent were assessed using a two-way repeated measures ANOVA [congruence effect: *F*(1,57) = 0.065; *P* = 0.8; group effect: *F*(1,57) = 0.705; *P* = 0.405]. Congruence and group effects on ROI-averaged aperiodic offset were also assessed using a two-way repeated measures ANOVA [congruence effect: *F*(1,57) = 0.101; *P* = 0.752; group effect: *F*(1,57) = 6.17; *P* = 0.016]. Spatial topographies of the aperiodic exponent (**C**) and offset (**D**) values across the cortex regardless of congruence for HC and Parkinson’s disease patients. Comparisons between groups were performed with ANOVAs for each ROI (FDR-corrected tests). ROIs with significant differences in aperiodic parameters between groups are represented in red. For exact test statistic values, see Supplementary Table 2. Spatial topographies of the aperiodic exponent (**E**) and offset (**F**) values across the cortex regardless of the group. Comparisons between conditions were performed with ANOVAs for each ROI (FDR corrected). ROIs with significant differences in aperiodic parameters between conditions are represented with red dots. For exact test statistic values, see Supplementary Table 2. HC, healthy control; ROI, region of interest. * indicates *P* < 0.05.

Distribution of aperiodic parameters for all ROIs was estimated for each group regardless of condition (Fig. 4C and D). We observed that the distribution pattern of the offset and exponent values seemed similar between HC and Parkinson’s disease patients. Regarding exponent distribution, no significant differences in values have been evidenced between groups (Fig. 4C). Aperiodic offsets in Parkinson’s disease were overall higher as compared to HC over left parieto-occipital regions and on both temporal lobes (Fig. 4D). For further details, see Supplementary Tables 2 and 10.

Distribution of aperiodic parameters across the cortex was also estimated for each condition regardless of group (Fig. 4E and F). The distribution pattern of aperiodic parameters seemed overall similar between conditions, too. However, significant differences in exponent parameters were found in precentral areas (Supplementary Table 1). Offset significantly differed in the right posterior cingulate and left precentral medial area (Supplementary Table 1).

Aperiodic exponents in incongruent conditions were greater than in congruent trials, but the effect sizes associated with these differences were very low (congruent: mean = 0.85, SD = 0.4; incongruent: mean = 0.86, SD = 0.4; size effect: 6.7^−3^). Same is found in offset values (congruent: mean = −21.03, SD = 0.6; incongruent: mean = −20.9, SD = 0.6; size effect: 0.149). For further details, see Supplementary Tables 2 and 10.

#### Task period effect on aperiodic parameters

We applied the same approach to test whether aperiodic parameters were different between resting state and during the task (pre- and post-stimulus) to check that our results did not only reflect baseline aperiodic activity.

##### Scalp level

**Figure 5:**
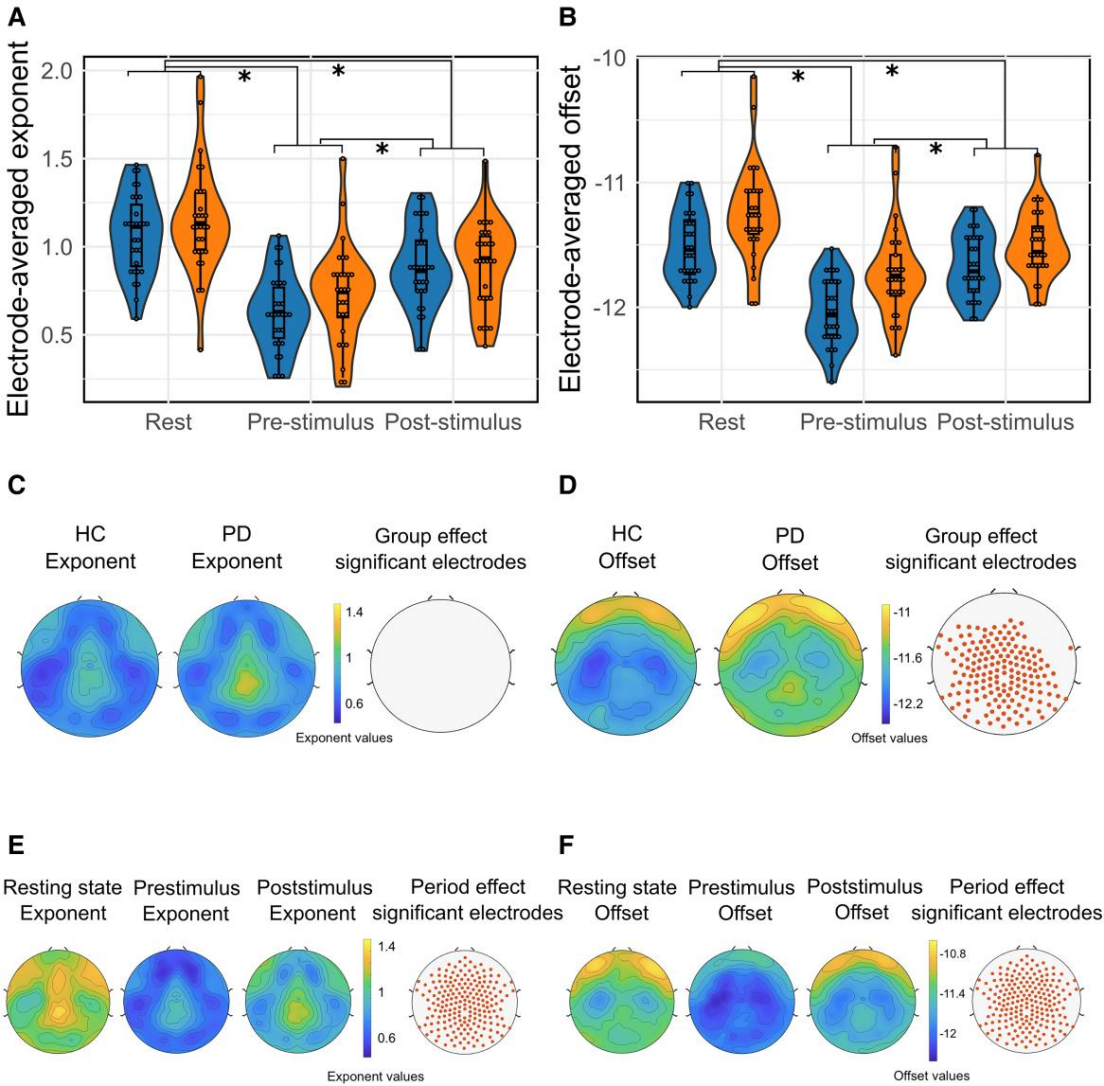
**Violin plots showing the distribution of electrode-averaged exponent (A) and offset (B) values by group (blue for HC, orange for Parkinson’s disease patients) and for each task period based on all electrodes.** Task period and group effects on electrode-averaged aperiodic exponent were assessed using a two-way repeated measures ANOVA [task period effect: *F*(2,114) = 82.47; *P* < 0.0001; group effect: *F*(1,57) = 1.48; *P* = 0.23]. Task period and group effects on electrode-averaged aperiodic offset were also assessed using a two-way repeated measures ANOVA [task period effect: *F*(2,114) = 74.02; *P* < 0.0001; group effect: *F*(1,57) = 14.71; *P* = 3.15^−4^]. Spatial topographies of the aperiodic exponent (**C**) and offset (**D**) values across the scalp regardless of task period for HC and Parkinson’s disease patients. Comparisons between groups were performed with two-way repeated measures ANOVAs for each electrode (FDR-corrected tests). Electrodes with significant differences in aperiodic parameters between HC and Parkinson’s disease patients are represented with red dots. For exact values of statistical tests, see Supplementary Table 3. Spatial topographies of the aperiodic exponent (**E**) and the offset (**F**) values across the scalp regardless of the group. Comparisons between task periods were performed with two-way repeated measures ANOVAs for each electrode (FDR-corrected tests). Electrodes with significant differences in aperiodic parameters between task periods are represented with red dots. For exact values of statistical tests, see Supplementary Table 3. HC, healthy control. * indicates *P* < 0.05

Electrode-averaged exponents did not significantly differ between groups [*F*(1,57) = 1.48; *P* = 0.229], but significantly differed according to the task period [*F*(2,114) = 82.47; *P* < 0.0001; Fig. 5A]. In addition to significant differences according to the task period [*F*(2,114) = 74.018; *P* < 0.0001], electrode-averaged offsets also differed between groups [*F*(1,57) = 14.71; *P* = 3.15^−4^; Fig. 5B].

The distribution of exponent values across the scalp for each group regardless of task period showed no significant group effect (Fig. 5C). However, the task period significantly differed regardless of group on all electrodes across the scalp (Fig. 5E). For further details, see Supplementary Table 3. *Post hoc* tests evidenced that exponents were greater (more negative slope) during the resting-state than during the post-stimulus period (*P* = 1.13^−8^) and even more so than during the pre-stimulus period (*P* = 2.09^−17^; see Supplementary Fig. 2A and Tables 5 and 6).

Distribution of the offset across the scalp was estimated for each group regardless of the task period (Fig. 5D). Offsets were higher in Parkinson’s disease patients over a large majority of electrodes across the scalp, except in frontal areas, especially on the right (Fig. 5F).

Regarding the offset distribution across the scalp for each task period regardless of the group, offsets were significantly different according to the period on the whole scalp. *Post hoc* comparisons showed that offsets during the resting-state period were higher than offsets during the pre-stimulus period on all electrodes (*P* = 6.5^−17^) and higher than offsets during the post-stimulus period on a large majority of electrodes across the scalp (*P* = 1.56^−5^). Moreover, post-stimulus offsets were significantly higher than pre-stimulus offsets on all electrodes across the scalp (*P* = 8.78^−9^; see Supplementary Fig. 2B and Tables 5 and 6).

##### Source level

ROI-averaged exponents did not significantly differ between groups [*F*(1,57) = 1.13; *P* = 0.292], while the task period had a significant effect on exponents [*F*(1.24,71.7) = 122.46; *P* < 0.0001]. The group had a significant effect on ROI-averaged offsets [*F*(1,57) = 6.079; *P* = 0.017]. ROI-averaged offsets also significantly differed according to the task period [*F*(1.34,76.23) = 122.46; *P* < 0.0001].

**Figure 6:**
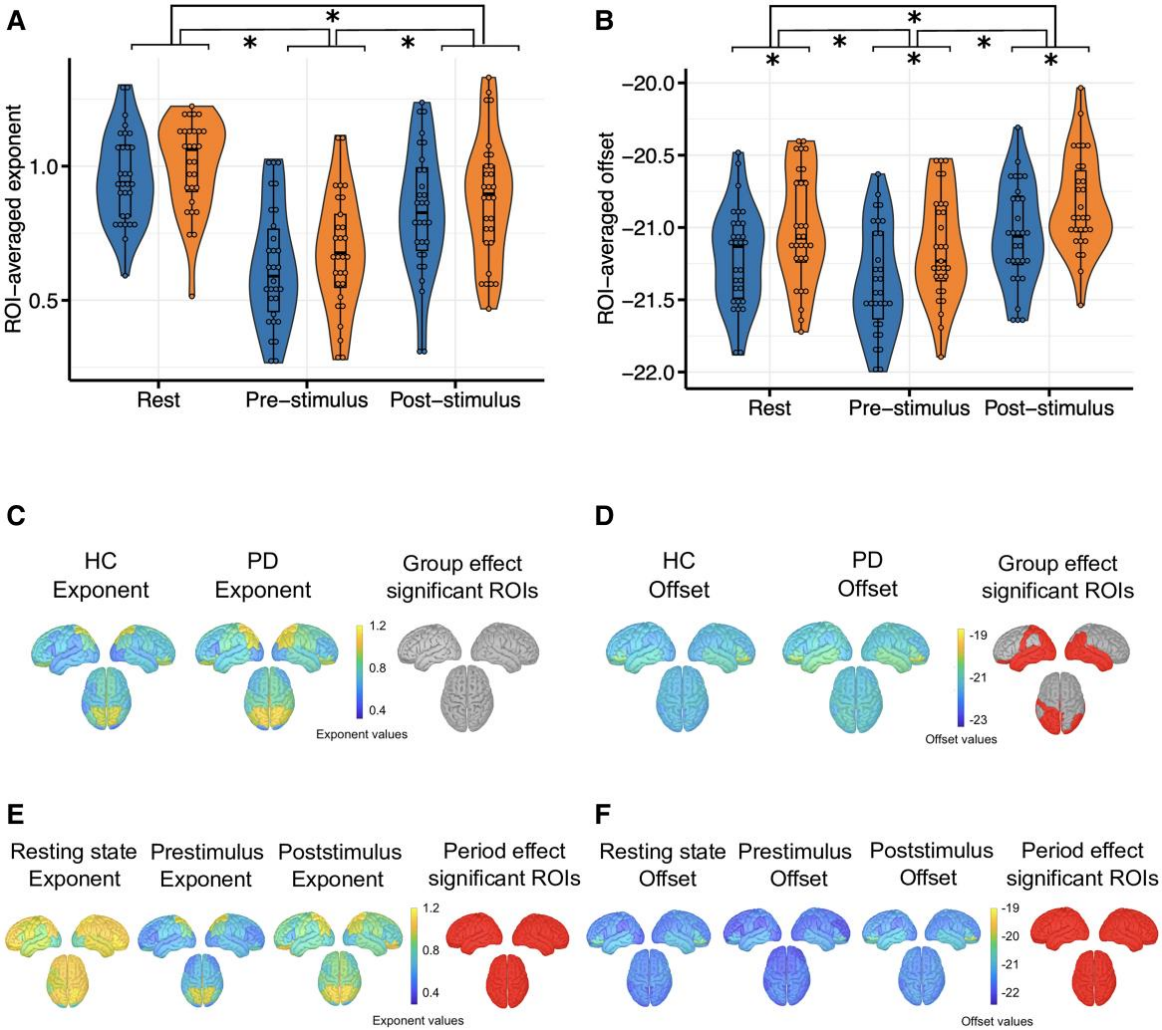
**Violin plots showing the distribution of ROI-averaged exponent (A) and offset (B) values by group (blue for HC, orange for Parkinson’s disease patients) and for each task period.** Task period and group effects on ROI-averaged aperiodic exponent were assessed using a two-way repeated measures ANOVA [task period effect: *F*(1.24, 71.7) = 122.46; *P* < 0.0001; group effect: *F*(1,57) = 1.13; *P* = 0.29]. Task period and group effects on ROI-averaged aperiodic offset were also assessed using a two-way repeated measures ANOVA [task period effect: *F*(1.34,76.23) = 122.46; *P* < 0.0001; group effect: *F*(1,57) = 6.079; *P* = 0.017]. Spatial topographies of the aperiodic exponent (**C**) and offset (**D**) values across the cortex regardless of task period for HC and Parkinson’s disease patients. Comparisons between groups were performed with two-way repeated measures ANOVAs for each ROI (FDR-corrected tests). ROIs with significant differences in aperiodic parameters between groups are represented in red. For exact values of statistical tests, see Supplementary Table 4. Spatial topographies of the aperiodic exponent (**E**) and offset (**F**) values across the cortex regardless of the group. Comparisons between task periods were performed with two-way repeated measures ANOVAs for each ROI (FDR-corrected tests). ROIs with significant differences in aperiodic parameters between task periods are represented in red. For exact values of statistical tests, see Supplementary Table 4. HC, healthy control; ROI, region of interest. * indicates *P* < 0.05.

The distribution of exponent values across the scalp for each group regardless of task period showed no significant group effect (Fig. 6C). However, the distribution of ROI-averaged exponents according to the task period regardless of the group showed a significant effect of the task period on all ROIs (Fig. 6E; Supplementary Table 1). Similar to the electrode-level results, *post hoc* tests evidenced that exponents were greater (more negative slope) during the resting-state than during the post-stimulus period (*P* = 2.33^−5^) and even more so than during the pre-stimulus period (*P* = 7.05^−20^; Supplementary Fig. 3A and Tables 7, 8 and 10).

Distribution of the offset across the cortex was estimated for each group regardless of the task period (Fig. 6C). Offsets were higher in Parkinson’s disease patients over occipital and left parietal cortical regions as well as in both temporal lobes (Fig. 6D; Supplementary Table 1). Regarding the offset distribution across the cortex for each task period regardless of the group, offsets were significantly different according to the period on the whole cortex. *Post hoc* tests showed that offsets during the post-stimulus period were greater than resting-state offsets (*P* = 9.36^−6^) that were themselves greater than offsets during the pre-stimulus period (*P* = 2.6^−5^; Supplementary Fig. 3B and Tables 7, 8 and 10).

### Correlation analysis

Correlation analyses did not find any significant association between behavioural or clinical variables and aperiodic parameters (see Supplementary Table 9).

## Discussion

The aim of this study was to test our hypothesis that aperiodic activity in the EEG power spectrum differs in Parkinson’s disease in comparison with HC during CAC, and that these aperiodic changes are linked to the CAC differences observed in Parkinson’s disease. Several key findings emerged from this study. First, we identified a diminished performance of CAC in Parkinson’s disease, as was expected from previous studies. Second, HD-EEG data evidenced that, in the post-stimulus period, the aperiodic offsets were higher in Parkinson’s disease patients in parieto-occipital areas at both scalp and cortex levels. In contrast, no group effect was found on the exponent distribution, except a congruence effect in motor areas. In addition, aperiodic parameters were both significantly different according to the task period: changes in offsets and exponents were found between resting-state, pre- and post-stimulus periods. Finally, no correlation between aperiodic activity and behaviour or clinical characteristics of the Parkinson’s disease population was found, thereby not supporting our hypothesis.

### Lower behavioural performance in Parkinson’s disease

Our results on the Simon task are consistent with the well-known CAC differences in Parkinson’s disease. Parkinson’s disease patients, as compared to HC, had an overall lower accuracy, as well as higher impulsive action selection and decreased strength of selective response suppression. No higher congruence effect or higher overall RTs were observed in Parkinson’s disease. Changes in the dynamic expression of CAC regarding impulsive action selection^27,29^ or suppression^25-28^ have been previously documented. The lack of a greater congruence effect on overall data may be due to several factors, such as the task settings,^82^ or according to the population characteristics that vary a lot among studies. Indeed, the Parkinson’s disease patients included in our study exhibited mild to moderate cognitive alteration.^83^ However, it has been found that the extent of cognitive disorders in Parkinson’s disease is quite heterogeneous,^84^ and symptom severity could be associated with a higher congruence effect or speed accuracy trade-off.^26,85^

### Group effect on aperiodic parameters

Our EEG analysis indicated an approximately symmetrical spatial distribution of the aperiodic parameters in HC and Parkinson’s disease patients, both at the scalp and source levels. These topographies are similar to those already observed in the literature in control^86^ and in patients.^61^ This suggests that aperiodic activity distribution pattern is preserved in Parkinson’s disease patients.

We observed significantly higher offsets in Parkinson’s disease patients versus control in the parietal–occipital regions. This group effect was found at both averaged and specific scales at scalp and cortex levels. Our results also evidenced that aperiodic offsets were significantly higher in Parkinson’s disease patients in comparison to HC during the post-stimulus phase but also more globally, regardless of task period. This result is in line with previous studies demonstrating greater offsets in Parkinson’s disease patients during resting state.^58,59,61^ More particularly, Wang *et al.*^61^ found that aperiodic offsets were greater in Parkinson’s disease patients versus control in parietal–occipital midline regions during resting state. Some studies suggested that aperiodic offset is linked to the rate of neural spiking,^87,88^ involving that these significant differences driven by aperiodic offset could be caused by increased neuronal spiking at the cortical level in Parkinson’s disease. This is supported by previous studies showing that neural spiking rate in Parkinson’s disease increased with motor symptom severity and disease progression.^89^

However, no significant difference has been evidenced between HC and Parkinson’s disease regarding exponent distribution, which contrasts with what has already been observed in the literature. For example, previous studies on aperiodic activity showed that exponents were significantly higher in Parkinson’s disease patients versus control during resting state.^57-61^ Recently, Helson *et al*.^57^ demonstrated that the exponent was significantly larger in Parkinson’s disease patients than in HC in all cortical areas except frontal ones, with stronger changes in sensory and motor areas. Physiological evidence has demonstrated that the aperiodic exponent can reflect the balance between excitation and inhibition.^40,90^ A flatter exponent is assumed to translate an increase in excitation over inhibition, while a steeper exponent is assumed to reflect the opposite. Moreover, previous studies also showed that impairments in dopaminergic and GABAergic neuronal activity are prominent in Parkinson’s disease, leading to an imbalance in E:I ratio.^91-93^

Our exponent results could be explained by the clinical criteria of our Parkinson’s disease population, given that our Parkinson’s disease patients were younger^59,60^ and had a less severe disease in comparison to the Parkinson’s disease population studied previously.^57,59,61^ We can hypothesize that slight to moderate symptoms are less associated with an imbalance in E:I ratio and consequently with more modest changes in aperiodic exponent.

### Congruence effect on aperiodic parameters

A congruence effect on central and posterior cingulate areas was only found at the cortical level. This result was not visible on a global scale, which underlines the very localized aspect of the congruence effect on aperiodic parameters. Aperiodic parameters were greater in incongruent conditions; however, the effect size was low for both aperiodic parameters, thus preventing strong conclusions. Very few studies have investigated aperiodic activity in the context of experimental cognitive tasks, and any with Parkinson’s disease patients, which limits the comparison of our results with the literature. Only one study focused on aperiodic neural activity during metacontrol, where Zhang *et al*.^94^ examined whether aperiodic activity reflects metacontrol analysing EEG and behavioural data of HC performing a Simon Go/NoGo task. They found higher aperiodic parameters in NoGo trials compared with Go trials in incongruent trials, as compared to congruent ones. However, McSweeney *et al.*^95^ investigated changes in aperiodic activity during early adolescence in a healthy population. They used an experimental task to assess the ability to suppress inappropriate responses with a Flanker task and found no significant effect of condition on aperiodic parameters. In our study, aperiodic parameters were greater in motor areas in incongruent conditions as compared to congruent ones. This difference can be associated with the motor execution of the task: incongruent conditions requiring greater motor control could be associated with an increase in aperiodic activity.

### Task period effect on aperiodic parameters

Our study showed significant changes in aperiodic parameters regarding the task period: both offsets and exponents were significantly different between resting-state, pre- and post-stimulus periods over a large majority of electrodes and brain areas. This result is consistent with those obtained in the study focusing on the link between aperiodic activity and metacontrol. Zhang *et al.*^94^ found that EEG power spectrum aperiodic activity reflected metacontrol states and particularly dynamic adjustments of metacontrol states to task demands. It has also been demonstrated that the aperiodic exponent among children reflects distinct cognitive processes.^96^ Moreover, a study from Lendner *et al*.^97^ also demonstrated that the aperiodic exponent delineates wakefulness from anaesthesia, rapid eye movement (REM) and non-REM sleep. In that sense, different arousal and cognitive states associated to the distinct task periods could explain these changes in both aperiodic parameters. These results suggest that aperiodic parameters changed over most brain regions according to the task execution.

### No correlation between behaviour or clinical features and aperiodic parameters

Contrary to our hypothesis, we did not find any significant relationship between behavioural measures (RT, accuracy, congruence effect, first bin of CAF, last slope of delta plots) and aperiodic parameters. The current literature provides mixed results about correlation between behaviour and aperiodic activity. Some studies showed an association between the aperiodic parameters and cognition: Ostlund *et al*.^42^ found that a lower exponent during resting state predicted better performance on the dual-task ‘Stopping task’, Smith *et al.*^51^ showed that the aperiodic exponent was associated with cognitive performance and Ouyang *et al*.^98^ also evidenced that aperiodic activity during resting state was associated with cognitive processing speed. However, a study aiming to investigate aperiodic parameters and their link with cognition did not show any correlation between aperiodic activity and behaviour.^95^ The absence of correlation between behaviour and aperiodic parameters in our study was not expected based on the literature but there is currently a limited number of studies that have shown this association and mixed results highlight the importance of attempting to further investigate this potential link.

Despite significant changes in aperiodic parameters, those were not correlated to clinical characteristics of the Parkinson’s disease population. This result is consistent with previous studies. Actually, no correlations between aperiodic parameters and UPDRS scores or motor-related sub-score have been found in the literature.^57,61^ Thus, this lack of clear association between aperiodic parameters and clinical criteria of the Parkinson’s disease population suggests that aperiodic parameters may not be useful as clinical biomarkers. Another hypothesis is that the population of Parkinson’s disease patients studied here did not experience sufficiently severe symptoms to reveal a clear association between clinical criteria and aperiodic parameters.

Regarding the sources of the observed differences, the absence of a congruence effect on aperiodic parameters at the scalp level, and at the source level in cortical regions where inter-group differences are observed, and the absence of a group and congruence interaction on the distribution of aperiodic parameters (scalp/source levels) suggest that the observed differences are not associated with cognitive control. This is in line with our results showing that the aperiodic differences observed between groups are the same whether we focus on resting-state or post-stimulus data. Regarding task effects, our comparisons of task-related activity to resting activity show that there is a clear task effect. However, it is true that we found no association between aspects of the task related to cognitive control (congruence, first bin incongruent accuracy, last delta slope) and aperiodic activity. Taken together, these results suggest that the task effects identified are likely due to general task execution mechanisms, rather than cognitive control *per se*.

### Limitations

An important limitation of our study, which also applies to most Parkinson’s disease-related studies, is that our Parkinson’s disease population is different in terms of clinical criteria to Parkinson’s disease populations in other studies, notably in age and disease severity, which can both influence cognitive control and EEG parameters. This limits our ability to compare our results and to establish firm conclusions. Regarding the methods, we used a brain template for source reconstruction, which limits the extent to which anatomical inter-individual variability is taken into account.

We also used a specific brain atlas, and it is not clear how the topography of aperiodic parameters may be impacted by using different atlas resolutions. Aperiodic components of the EEG have been linked to string subject-specific properties, which could explain at least in part the variability in our results.^99^ Furthermore, it would have been preferable to assess the ability to discriminate the colours used in the Simon task in order to exclude any bias linked to difficulties in chromatic discrimination due to dopamine deficits in Parkinson’s disease.^100^ The small sample size used in our study also prevents the generalization of our results, motivating the validation on independent data sets.

### Perspectives

Several open questions remain with this study, notably the potential link between aperiodic parameters and cognitive differences in Parkinson’s disease. While our study did not provide support for an association between behaviour and aperiodic activities, future efforts could include the use of other experimental cognitive tasks in which Parkinson’s disease patients show behavioural alterations, such as a visual working memory task. Donoghue *et al*.^35^ have already shown that event-related changes in aperiodic parameters can predict individual working memory performance; therefore, it could be tested whether or not this association holds in Parkinson’s disease patients. Moreover, it would be particularly interesting to study the complementary aspects between the periodic and aperiodic components by exploring periodic activity, on which the focus is traditionally placed. Furthermore, this study highlights the importance of considering aperiodic activity in EEG analyses and provides further evidence that this component is linked to pathologies such as Parkinson’s disease. In that sense, we suggest that studies focusing on the identification of clinical biomarkers of cognitive decline in Parkinson’s disease using machine learning should consider aperiodic activity as an interesting feature.

## Supplementary Material

fcae306_Supplementary_Data

## Data Availability

The channel file and all the MATLAB and R codes used for pre-processing and data analysis are publicly available at https://github.com/noemiemonchy/PD-SIMON.
