# Supplementary material for: Changes in electrophysiological aperiodic activity during cognitive control in Parkinson’s disease
Source: Brain Commun. 2024 Sep 7;6(5):fcae306. doi: 10.1093/braincomms/fcae306 (PMC11411214; doi:10.1093/braincomms/fcae306)
Supplement: fcae306_Supplementary_Data [file fcae306_supplementary_data.pdf]

Supplementary Material

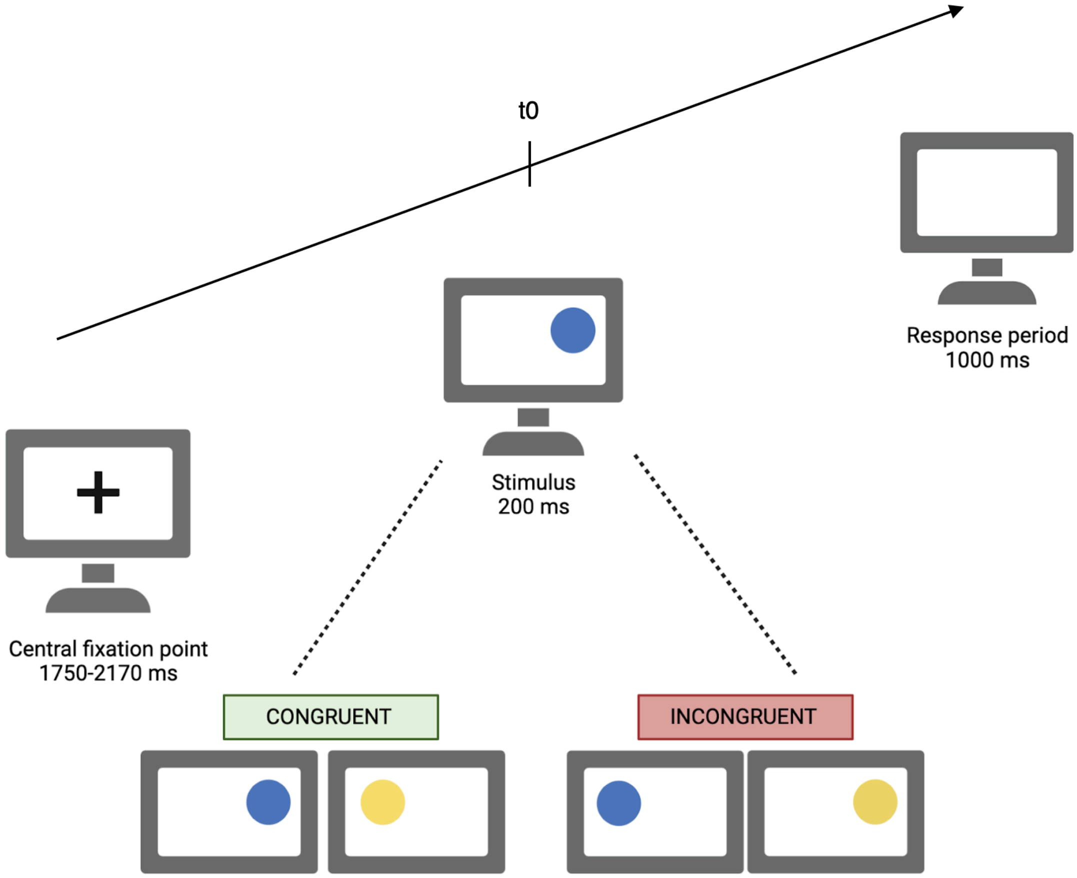

Supplementary Figure 1 Overview of the Simon task design. Each trial began with a black fixation cross at the screen center during a randomly defined period between 1750 and 2170 ms. Then, the stimulus was displayed during 200 ms. Participants had 1000 ms to answer by pressing the button. Two conditions could occur: congruent when the side of presentation of the circle and the required response side triggered the same response, and incongruent when both items did not lead to the same response.

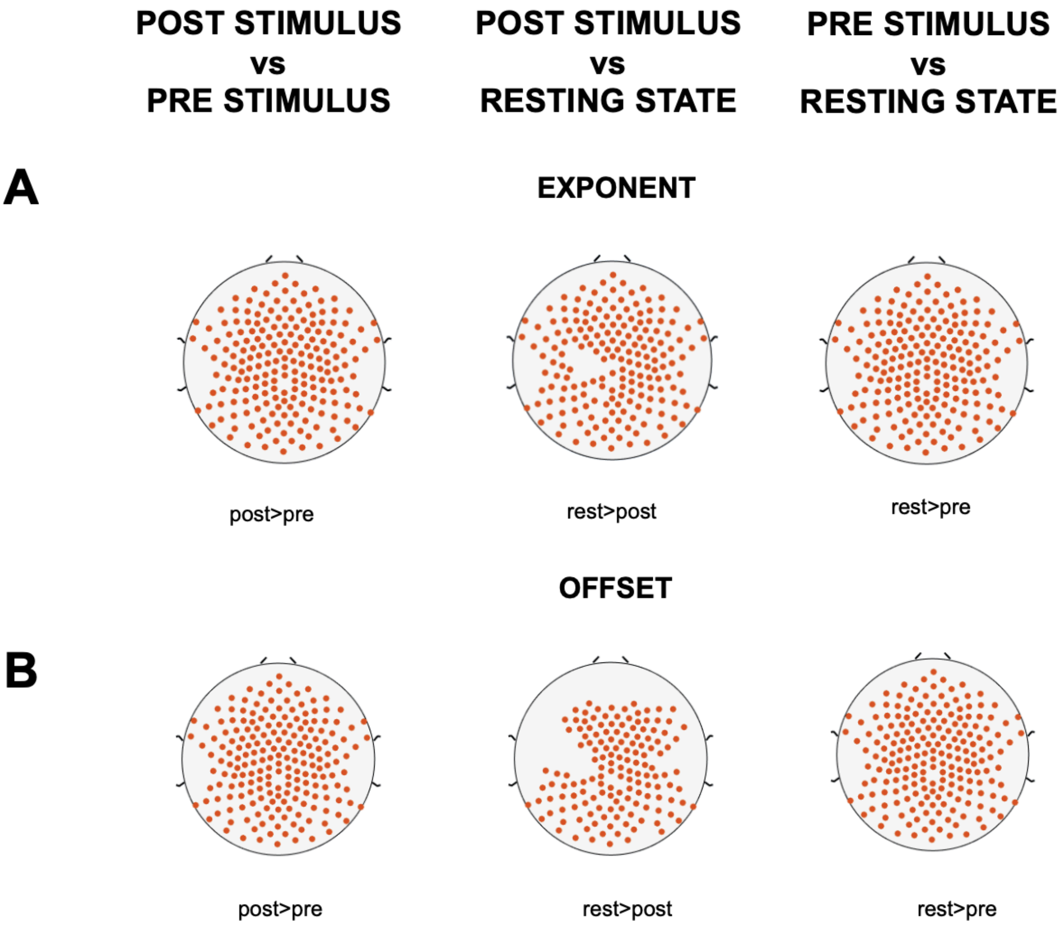

Supplementary Figure 2 P-values distribution of t-tests (FDR-corrected) performed for each electrode across the scalp. Comparisons of aperiodic exponent (A) and aperiodic offset (B) values were realized according to the task period. For exact values of statistical tests, see Supplementary Tables 5 and 6. Electrodes with significant differences in aperiodic parameters between each period are represented with red dots.

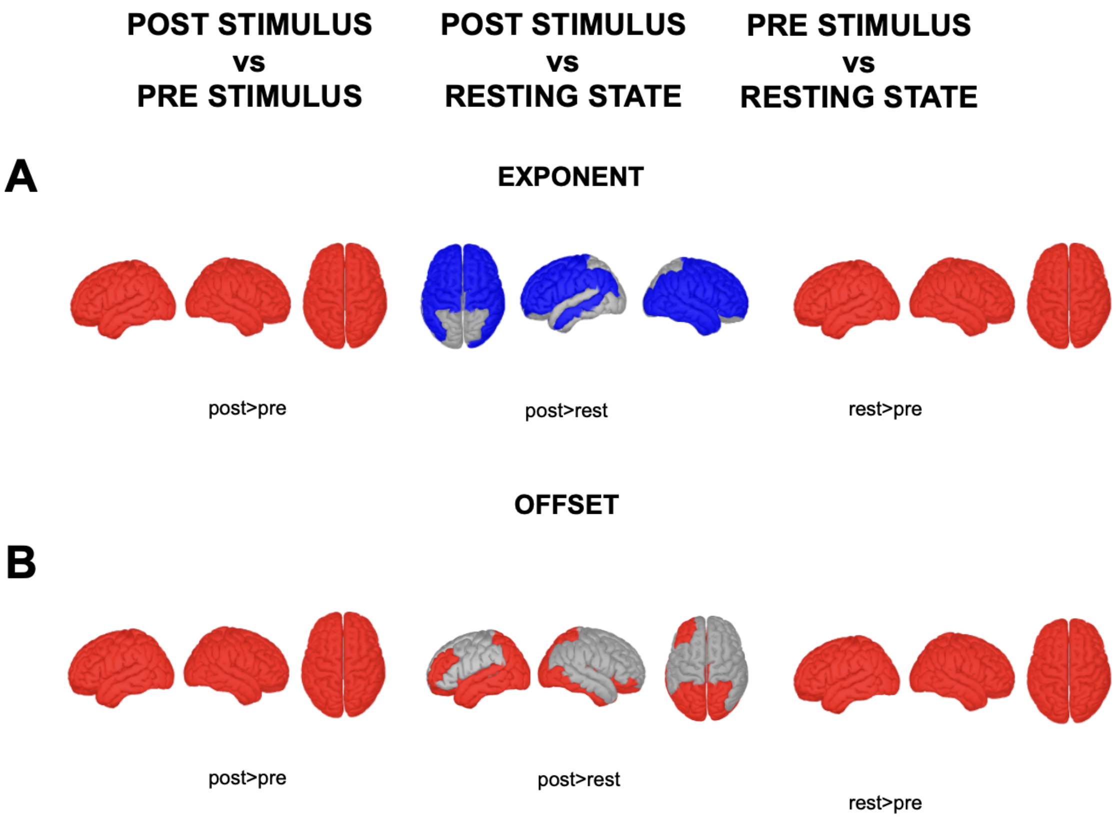

Supplementary Figure 3 P-values distribution of t-tests (FDR-corrected) performed for each ROI across the cortex. Comparisons of aperiodic exponent (A) and aperiodic offset (B) values were realized according to the task period. For exact values of statistical tests, see Supplementary Tables 7 and 8. ROIs with significant differences in aperiodic parameters between each period are represented in color. Red color corresponds to significant differences in the same direction as the legend while blue color indicates significant differences in the opposite direction of comparison.

Supplementary Table 1 Statistical values of ANOVAs assessing group and congruence effect on aperiodic parameters at scalp level.

	EXPONENT				OFFSET			
	group		congruence		group		cong	
Electrode	F	p	F	p	F	p	F	p
1	0,46	0,99	0,30	0,94	0,01	0,93	0,13	0,97
2	1,56	0,99	0,41	0,91	0,28	0,66	2,73	0,55
3	2,70	0,99	2,76	0,68	0,24	0,69	0,72	0,82
4	2,21	0,99	0,01	1,00	0,31	0,66	0,25	0,93
5	0,35	0,99	1,24	0,79	0,01	0,93	5,62	0,50
6	0,21	0,99	0,34	0,93	0,57	0,53	1,36	0,79
7	0,09	0,99	0,58	0,89	1,18	0,36	0,45	0,86
8	0,11	0,99	4,42	0,48	4,37	0,09	6,60	0,50
9	1,24	0,99	0,14	0,94	4,17	0,10	0,17	0,97
10	0,41	0,99	0,20	0,94	0,28	0,66	0,32	0,89
11	1,91	0,99	0,03	1,00	0,75	0,47	0,77	0,82
12	0,12	0,99	1,39	0,78	0,36	0,63	1,28	0,81
13	0,20	0,99	0,86	0,85	0,31	0,66	2,53	0,57
14	0,19	0,99	0,01	1,00	2,38	0,20	0,02	0,98
15	0,00	0,99	1,39	0,78	1,93	0,25	0,88	0,82
16	0,11	0,99	0,15	0,94	2,20	0,22	0,08	0,97
17	0,04	0,99	5,07	0,48	2,92	0,17	2,71	0,55
18	0,29	0,99	0,51	0,89	0,06	0,87	0,01	0,98
19	0,06	0,99	0,06	0,98	0,12	0,80	0,00	0,98
20	0,00	0,99	0,01	1,00	0,93	0,42	0,32	0,89
21	0,07	0,99	0,24	0,94	2,50	0,19	0,45	0,86
22	0,46	0,99	0,38	0,91	3,16	0,15	0,05	0,97
23	0,25	0,99	0,02	1,00	1,54	0,29	0,11	0,97
24	0,00	0,99	0,11	0,95	3,92	0,11	0,09	0,97
25	0,17	0,99	0,61	0,89	0,04	0,89	2,65	0,55
26	0,50	0,99	0,84	0,85	0,36	0,63	3,31	0,55
27	0,04	0,99	0,07	0,98	0,01	0,93	0,15	0,97
28	0,19	0,99	0,22	0,94	1,48	0,30	0,14	0,97
29	0,00	0,99	4,16	0,48	2,23	0,22	2,96	0,55
30	0,24	0,99	0,52	0,89	2,49	0,19	1,15	0,82
31	2,23	0,99	1,86	0,77	0,04	0,89	2,35	0,62
32	0,19	0,99	0,28	0,94	0,30	0,66	1,53	0,77
33	0,11	0,99	0,11	0,95	0,01	0,93	0,01	0,98
34	0,01	0,99	1,53	0,78	1,17	0,36	1,67	0,76
35	0,02	0,99	1,00	0,85	5,34	0,07	1,84	0,75
36	0,20	0,99	5,68	0,46	2,51	0,19	4,34	0,50
37	0,12	0,99	0,04	0,98	0,31	0,66	0,52	0,86
38	0,55	0,99	1,15	0,81	0,62	0,51	1,07	0,82
39	0,09	0,99	3,38	0,60	5,06	0,08	5,34	0,50
40	0,51	0,99	0,00	1,00	1,50	0,30	0,00	0,98
41	0,00	0,99	2,28	0,75	2,79	0,17	1,17	0,82

42	0,00	0,99	1,13	0,81	1,90	0,25	0,17	0,97
43	0,01	0,99	1,31	0,78	2,22	0,22	2,93	0,55
44	0,74	0,99	4,38	0,48	4,53	0,09	4,16	0,50
45	2,15	0,99	0,03	0,98	4,22	0,10	0,00	0,99
46	0,31	0,99	0,06	0,98	1,11	0,37	0,04	0,97
47	0,02	0,99	0,00	1,00	2,45	0,19	0,05	0,97
48	0,17	0,99	0,02	1,00	3,21	0,15	0,02	0,98
49	0,07	0,99	3,58	0,60	3,59	0,13	0,73	0,82
50	0,71	0,99	0,01	1,00	7,73	0,03	0,03	0,98
51	0,27	0,99	2,87	0,66	5,95	0,06	0,78	0,82
52	0,05	0,99	0,00	1,00	2,91	0,17	0,00	0,99
53	0,12	0,99	0,10	0,96	0,70	0,48	0,36	0,89
54	0,23	0,99	1,73	0,77	4,53	0,09	1,51	0,77
55	0,02	0,99	1,75	0,77	1,65	0,28	2,91	0,55
56	0,06	0,99	0,04	0,98	1,90	0,25	0,87	0,82
57	0,00	0,99	3,22	0,60	2,50	0,19	0,84	0,82
58	0,02	0,99	0,00	1,00	2,61	0,19	0,66	0,83
59	0,00	0,99	0,04	0,98	3,25	0,15	0,46	0,86
60	0,26	0,99	12,49	0,08	0,85	0,44	11,35	0,10
61	0,53	0,99	1,81	0,77	6,43	0,05	3,05	0,55
62	0,03	0,99	0,01	1,00	1,10	0,37	0,09	0,97
63	1,07	0,99	1,51	0,78	3,61	0,13	0,12	0,97
64	0,19	0,99	0,17	0,94	6,12	0,05	0,09	0,97
65	0,02	0,99	0,00	1,00	1,74	0,27	0,18	0,97
66	0,03	0,99	0,80	0,85	2,57	0,19	1,25	0,81
67	0,13	0,99	0,28	0,94	2,60	0,19	0,97	0,82
68	0,00	0,99	0,91	0,85	2,18	0,22	0,94	0,82
69	0,03	0,99	0,12	0,95	2,09	0,23	0,00	0,98
70	0,13	0,99	0,71	0,86	1,59	0,29	0,29	0,90
71	0,48	0,99	1,63	0,78	4,54	0,09	2,01	0,70
72	0,00	0,99	0,78	0,85	3,09	0,16	0,51	0,86
73	0,10	0,99	0,22	0,94	3,07	0,16	0,52	0,86
74	0,24	0,99	0,00	1,00	6,14	0,05	0,30	0,89
75	0,17	0,99	0,02	1,00	4,78	0,08	0,06	0,97
76	0,29	0,99	1,43	0,78	0,95	0,41	0,43	0,86
77	0,12	0,99	0,14	0,94	3,08	0,16	0,04	0,97
78	0,28	0,99	0,60	0,89	2,55	0,19	1,54	0,77
79	1,44	0,99	0,51	0,89	4,18	0,10	0,64	0,84
80	3,56	0,99	3,54	0,60	4,88	0,08	4,54	0,50
81	0,51	0,99	1,33	0,78	8,01	0,03	0,02	0,98
82	0,09	0,99	0,06	0,98	5,36	0,07	0,54	0,86
83	0,12	0,99	0,22	0,94	4,47	0,09	0,36	0,89
84	0,56	0,99	0,94	0,85	4,31	0,09	0,70	0,82
85	0,33	0,99	0,82	0,85	4,67	0,08	0,77	0,82
86	0,53	0,99	6,79	0,40	3,29	0,15	6,14	0,50
87	0,74	0,99	0,20	0,94	4,76	0,08	0,33	0,89
88	2,10	0,99	0,56	0,89	4,95	0,08	1,60	0,76
89	0,98	0,99	0,46	0,90	9,31	0,02	0,00	0,98
90	0,41	0,99	0,55	0,89	9,49	0,02	0,12	0,97

91	1,03	0,99	0,01	1,00	9,98	0,02	0,02	0,98
92	0,84	0,99	1,33	0,78	9,47	0,02	1,48	0,77
93	0,33	0,99	0,16	0,94	8,99	0,02	0,30	0,89
94	0,65	0,99	2,33	0,75	8,04	0,03	1,93	0,72
95	2,78	0,99	0,48	0,90	9,31	0,02	0,04	0,97
96	4,35	0,99	2,05	0,77	9,30	0,02	1,25	0,81
97	6,87	0,99	1,38	0,78	15,04	0,01	2,62	0,55
98	1,23	0,99	0,09	0,96	12,98	0,01	0,12	0,97
99	0,30	0,99	0,15	0,94	12,13	0,01	0,18	0,97
100	0,10	0,99	0,87	0,85	7,76	0,03	1,42	0,78
101	0,00	0,99	0,00	1,00	4,50	0,09	0,02	0,98
102	1,25	0,99	0,04	0,98	14,32	0,01	0,02	0,98
103	0,82	0,99	0,24	0,94	10,68	0,01	0,30	0,89
104	3,18	0,99	0,35	0,93	10,74	0,01	0,02	0,98
105	0,33	0,99	1,46	0,78	10,38	0,01	1,47	0,77
106	0,19	0,99	1,76	0,77	4,86	0,08	1,16	0,82
107	0,61	0,99	2,53	0,71	0,82	0,45	3,34	0,55
108	0,07	0,99	0,01	1,00	10,86	0,01	0,05	0,97
109	0,17	0,99	0,02	1,00	10,67	0,01	1,37	0,79
110	0,81	0,99	0,05	0,98	9,11	0,02	0,68	0,82
111	4,92	0,99	0,77	0,85	14,99	0,01	2,09	0,68
112	0,14	0,99	0,04	0,98	8,93	0,02	0,22	0,95
113	0,32	0,99	0,41	0,91	8,23	0,03	0,69	0,82
114	0,52	0,99	0,15	0,94	5,97	0,06	0,01	0,98
115	0,01	0,99	0,18	0,94	11,07	0,01	0,05	0,97
116	1,22	0,99	1,79	0,77	15,62	0,01	3,80	0,51
117	5,08	0,99	0,39	0,91	17,39	0,00	3,01	0,55
118	3,16	0,99	0,82	0,85	13,13	0,01	2,92	0,55
119	3,67	0,99	1,07	0,83	15,18	0,01	0,06	0,97
120	3,51	0,99	0,09	0,96	7,95	0,03	0,05	0,97
121	0,76	0,99	10,24	0,13	1,58	0,29	8,55	0,33
122	1,84	0,99	1,79	0,77	6,97	0,04	1,62	0,76
123	0,03	0,99	3,15	0,60	10,26	0,01	4,07	0,50
124	0,00	0,99	0,23	0,94	18,38	0,00	0,38	0,88
125	0,04	0,99	0,42	0,91	19,48	0,00	0,02	0,98
126	0,12	0,99	0,05	0,98	14,50	0,01	0,30	0,89
127	0,74	0,99	0,00	1,00	12,35	0,01	0,47	0,86
128	0,62	0,99	1,46	0,78	8,00	0,03	3,53	0,55
129	0,06	0,99	0,71	0,86	2,13	0,22	0,11	0,97
130	2,32	0,99	4,15	0,48	5,26	0,07	0,97	0,82
131	0,32	0,99	2,56	0,71	1,73	0,27	1,77	0,75
132	0,02	0,99	0,22	0,94	2,00	0,24	0,49	0,86
133	0,43	0,99	0,13	0,94	20,88	0,00	0,01	0,98
134	0,80	0,99	0,27	0,94	21,70	0,00	0,05	0,97
135	0,80	0,99	0,20	0,94	11,97	0,01	0,56	0,86
136	1,20	0,99	0,04	0,98	12,97	0,01	0,01	0,98
137	0,30	0,99	0,84	0,85	9,84	0,02	0,09	0,97
138	0,01	0,99	6,04	0,46	2,84	0,17	1,71	0,76
139	0,09	0,99	0,19	0,94	2,81	0,17	0,04	0,97

**140**	**0,41**	**0,99**	**2,49**	**0,71**	**2,71**	**0,18**	**2,70**	**0,55**
**141**	**0,41**	**0,99**	**1,89**	**0,77**	**5,98**	**0,06**	**3,22**	**0,55**
**142**	**0,25**	**0,99**	**1,16**	**0,81**	**16,05**	**0,01**	**0,69**	**0,82**
**143**	**0,05**	**0,99**	**0,52**	**0,89**	**9,89**	**0,02**	**0,86**	**0,82**
**144**	**0,34**	**0,99**	**0,17**	**0,94**	**10,25**	**0,01**	**0,47**	**0,86**
**145**	**0,87**	**0,99**	**0,01**	**1,00**	**15,27**	**0,01**	**0,11**	**0,97**
**146**	**0,11**	**0,99**	**3,30**	**0,60**	**4,84**	**0,08**	**2,12**	**0,68**
**147**	**0,03**	**0,99**	**3,01**	**0,63**	**1,21**	**0,35**	**2,83**	**0,55**
**148**	**0,37**	**0,99**	**1,15**	**0,81**	**4,79**	**0,08**	**1,78**	**0,75**
**149**	**1,79**	**0,99**	**1,33**	**0,78**	**7,30**	**0,03**	**0,72**	**0,82**
**150**	**0,03**	**0,99**	**1,31**	**0,78**	**10,19**	**0,01**	**0,89**	**0,82**
**151**	**0,12**	**0,99**	**1,37**	**0,78**	**3,83**	**0,11**	**2,67**	**0,55**
**152**	**0,11**	**0,99**	**1,87**	**0,77**	**5,20**	**0,07**	**2,29**	**0,63**
**153**	**0,04**	**0,99**	**5,50**	**0,46**	**4,58**	**0,09**	**3,96**	**0,50**
**154**	**0,15**	**0,99**	**4,33**	**0,48**	**4,89**	**0,08**	**3,90**	**0,50**
**155**	**0,11**	**0,99**	**0,79**	**0,85**	**3,92**	**0,11**	**0,46**	**0,86**
**156**	**0,98**	**0,99**	**0,20**	**0,94**	**2,07**	**0,23**	**0,13**	**0,97**
**157**	**0,17**	**0,99**	**0,39**	**0,91**	**6,54**	**0,05**	**0,91**	**0,82**
**158**	**0,11**	**0,99**	**0,05**	**0,98**	**7,16**	**0,04**	**0,10**	**0,97**
**159**	**0,65**	**0,99**	**0,44**	**0,91**	**7,80**	**0,03**	**1,02**	**0,82**
**160**	**0,02**	**0,99**	**0,47**	**0,90**	**4,83**	**0,08**	**0,85**	**0,82**
**161**	**0,45**	**0,99**	**4,81**	**0,48**	**5,48**	**0,07**	**4,41**	**0,50**
**162**	**0,90**	**0,99**	**0,82**	**0,85**	**7,87**	**0,03**	**0,70**	**0,82**
**163**	**1,12**	**0,99**	**1,02**	**0,85**	**6,08**	**0,06**	**0,86**	**0,82**
**164**	**2,11**	**0,99**	**14,66**	**0,06**	**7,22**	**0,03**	**13,78**	**0,09**
**165**	**0,13**	**0,99**	**3,19**	**0,60**	**2,61**	**0,19**	**4,22**	**0,50**
**166**	**0,25**	**0,99**	**0,90**	**0,85**	**1,88**	**0,25**	**0,93**	**0,82**
**167**	**1,08**	**0,99**	**8,19**	**0,24**	**4,65**	**0,08**	**4,98**	**0,50**
**168**	**0,24**	**0,99**	**0,29**	**0,94**	**5,17**	**0,08**	**0,01**	**0,98**
**169**	**0,01**	**0,99**	**2,49**	**0,71**	**5,53**	**0,07**	**2,94**	**0,55**
**170**	**0,08**	**0,99**	**0,21**	**0,94**	**8,79**	**0,02**	**0,39**	**0,88**
**171**	**0,08**	**0,99**	**5,42**	**0,46**	**3,37**	**0,14**	**5,42**	**0,50**
**172**	**0,25**	**0,99**	**0,00**	**1,00**	**3,00**	**0,16**	**0,12**	**0,97**
**173**	**0,72**	**0,99**	**0,19**	**0,94**	**3,13**	**0,16**	**0,17**	**0,97**
**174**	**2,41**	**0,99**	**0,00**	**1,00**	**7,13**	**0,04**	**0,21**	**0,96**
**175**	**0,09**	**0,99**	**0,57**	**0,89**	**0,30**	**0,66**	**0,84**	**0,82**
**176**	**0,08**	**0,99**	**2,08**	**0,77**	**1,69**	**0,28**	**0,97**	**0,82**
**177**	**0,01**	**0,99**	**4,70**	**0,48**	**2,51**	**0,19**	**4,70**	**0,50**
**178**	**0,00**	**0,99**	**0,00**	**1,00**	**3,28**	**0,15**	**0,00**	**0,98**
**179**	**0,61**	**0,99**	**0,73**	**0,86**	**0,08**	**0,84**	**0,60**	**0,85**
**180**	**0,27**	**0,99**	**0,26**	**0,94**	**0,10**	**0,83**	**0,82**	**0,82**
**181**	**0,27**	**0,99**	**0,68**	**0,87**	**0,86**	**0,44**	**0,54**	**0,86**
**182**	**0,05**	**0,99**	**0,00**	**1,00**	**1,26**	**0,35**	**0,34**	**0,89**
**183**	**0,23**	**0,99**	**1,28**	**0,78**	**2,14**	**0,22**	**0,00**	**0,98**
**184**	**0,17**	**0,99**	**8,63**	**0,24**	**1,24**	**0,35**	**5,02**	**0,50**
**185**	**0,00**	**0,99**	**0,69**	**0,87**	**1,19**	**0,36**	**0,16**	**0,97**
**186**	**0,45**	**0,99**	**1,46**	**0,78**	**0,02**	**0,93**	**1,65**	**0,76**
**187**	**0,22**	**0,99**	**1,83**	**0,77**	**0,02**	**0,93**	**1,04**	**0,82**
**188**	**0,41**	**0,99**	**0,16**	**0,94**	**0,04**	**0,89**	**0,08**	**0,97**

189	0,82	0,99	0,59	0,89	0,02	0,93	0,02	0,98
190	0,77	0,99	0,01	1,00	0,00	0,98	0,10	0,97
191	0,74	0,99	0,10	0,96	0,66	0,50	0,01	0,98
192	0,02	0,99	0,47	0,90	0,58	0,53	0,53	0,86
193	0,77	0,99	3,29	0,60	0,06	0,87	4,26	0,50
194	0,21	0,99	0,18	0,94	0,03	0,91	0,46	0,86
195	4,72	0,99	1,91	0,77	1,24	0,35	0,00	0,99
196	1,28	0,99	0,54	0,89	0,01	0,93	0,16	0,97
197	0,00	0,99	1,74	0,77	1,56	0,29	0,82	0,82
198	0,29	0,99	4,98	0,48	2,36	0,20	4,71	0,50
199	3,75	0,99	0,94	0,85	9,00	0,02	1,05	0,82

Supplementary Table 2 Statistical values of ANOVAs assessing group and congruence effect on aperiodic parameters at the cortex level.

	EXPONENT				OFFSET			
	group		congruence		group		congruence	
ROI	F	p	F	p	F	p	F	p
1	0,85	0,72	2,73	0,39	4,59	0,08	2,27	0,39
2	1,82	0,66	0,08	0,88	3,48	0,12	0,05	0,93
3	1,67	0,66	0,06	0,89	3,89	0,10	0,57	0,71
4	1,57	0,66	1,77	0,48	3,07	0,13	0,85	0,62
5	0,48	0,84	0,06	0,89	2,68	0,15	1,41	0,49
6	0,37	0,85	0,63	0,70	0,85	0,40	1,94	0,43
7	2,91	0,66	0,57	0,70	8,70	0,03	0,06	0,93
8	1,63	0,66	0,58	0,70	10,13	0,02	2,20	0,39
9	7,44	0,54	0,37	0,76	14,51	0,01	0,02	0,94
10	0,08	0,94	0,46	0,72	4,52	0,08	0,02	0,94
11	2,53	0,66	0,28	0,76	0,06	0,83	0,09	0,93
12	2,90	0,66	0,02	0,93	0,03	0,86	0,08	0,93
13	0,27	0,85	0,94	0,62	10,23	0,02	1,04	0,56
14	0,02	0,99	0,23	0,79	8,15	0,03	1,34	0,51
15	4,29	0,66	0,08	0,88	12,91	0,01	0,00	0,98
16	5,12	0,66	0,01	0,97	5,88	0,05	0,20	0,83
17	2,17	0,66	1,10	0,60	14,67	0,01	1,71	0,43
18	0,23	0,86	0,53	0,70	5,92	0,05	0,53	0,71
19	1,72	0,66	0,10	0,88	3,16	0,13	0,60	0,71
20	0,17	0,91	0,43	0,73	1,02	0,36	0,02	0,94
21	0,00	0,99	1,04	0,61	5,57	0,06	1,83	0,43
22	0,00	0,99	4,37	0,21	2,88	0,14	3,37	0,37
23	0,00	0,99	5,39	0,15	12,33	0,01	6,60	0,13
24	1,23	0,66	0,13	0,86	10,71	0,02	0,00	0,98
25	0,38	0,85	1,45	0,53	4,00	0,10	2,72	0,39
26	0,67	0,80	0,52	0,70	4,84	0,08	0,29	0,82

27	0,26	0,85	2,25	0,44	8,18	0,03	2,01	0,42
28	0,00	0,99	1,68	0,49	5,41	0,06	0,52	0,71
29	0,00	0,99	2,02	0,46	1,82	0,23	2,64	0,39
30	0,00	0,99	2,21	0,44	1,57	0,26	1,12	0,54
31	3,74	0,66	2,23	0,44	10,14	0,02	4,16	0,28
32	0,51	0,84	0,61	0,70	6,91	0,04	0,00	0,98
33	2,83	0,66	17,23	0,01	5,01	0,07	15,79	0,01
34	0,91	0,71	10,43	0,03	2,46	0,16	7,22	0,10
35	0,95	0,71	0,34	0,76	9,37	0,02	0,23	0,83
36	0,05	0,96	6,70	0,08	5,68	0,06	5,53	0,18
37	0,31	0,85	1,89	0,46	0,15	0,73	2,91	0,39
38	0,01	0,99	4,72	0,19	0,27	0,65	3,26	0,37
39	0,08	0,94	3,23	0,34	2,89	0,14	2,43	0,39
40	0,06	0,94	6,68	0,08	1,40	0,29	7,32	0,10
41	0,49	0,84	0,29	0,76	0,51	0,53	0,04	0,94
42	0,32	0,85	0,95	0,62	0,29	0,64	1,29	0,51
43	0,44	0,85	2,78	0,39	8,00	0,03	2,43	0,39
44	0,65	0,80	1,28	0,57	6,15	0,05	0,81	0,62
45	2,96	0,66	0,31	0,76	6,63	0,04	0,05	0,93
46	1,46	0,66	3,86	0,26	1,77	0,23	2,79	0,39
47	1,07	0,69	10,08	0,03	3,17	0,13	8,05	0,10
48	1,50	0,66	12,77	0,02	3,19	0,13	12,24	0,03
49	1,61	0,66	9,85	0,04	3,08	0,13	8,17	0,10
50	0,57	0,83	8,98	0,05	2,31	0,18	5,12	0,18
51	0,30	0,85	0,00	0,98	7,29	0,04	0,01	0,96
52	1,33	0,66	8,31	0,06	8,09	0,03	5,32	0,18
53	0,14	0,93	1,87	0,46	1,04	0,36	2,19	0,39
54	0,00	0,99	2,14	0,44	2,24	0,18	3,10	0,38
55	0,06	0,94	0,00	0,97	1,03	0,36	0,50	0,72
56	1,39	0,66	3,17	0,34	0,10	0,78	1,57	0,46
57	0,07	0,94	0,71	0,70	2,68	0,15	0,83	0,62
58	0,09	0,94	0,55	0,70	2,73	0,15	1,69	0,43
59	2,24	0,66	6,95	0,08	7,58	0,03	3,80	0,32
60	2,08	0,66	0,30	0,76	3,01	0,13	0,19	0,83
61	1,24	0,66	1,52	0,52	5,66	0,06	0,14	0,88
62	1,07	0,69	1,15	0,59	4,66	0,08	2,53	0,39
63	3,48	0,66	2,57	0,41	3,10	0,13	1,81	0,43
64	4,32	0,66	0,02	0,93	3,93	0,10	0,23	0,83
65	2,13	0,66	0,58	0,70	7,14	0,04	1,22	0,52
66	1,63	0,66	0,20	0,80	8,21	0,03	0,33	0,80
67	0,29	0,85	0,05	0,89	4,62	0,08	0,22	0,83
68	0,99	0,71	1,25	0,57	3,44	0,12	0,37	0,79

Supplementary Table 3 Statistical values of ANOVAs assessing group and period effect on aperiodic parameters at the scalp level.

	EXPONENT				OFFSET			
	group		period		group		period	
Electrode	F	p	F	p	F	p	F	p
1	0,03	0,97	69,54	2,25E-19	2,63	1,24E-01	58,52	9,30E-17
2	0,33	0,78	62,30	3,56E-18	0,91	3,57E-01	45,65	1,03E-14
3	0,85	0,63	72,83	7,60E-20	0,57	4,59E-01	52,31	8,84E-16
4	0,19	0,84	66,13	7,42E-19	1,90	1,87E-01	39,90	1,45E-13
5	0,30	0,79	60,86	6,90E-18	4,85	3,88E-02	40,31	1,17E-13
6	0,70	0,65	49,92	9,04E-16	6,52	1,83E-02	44,94	1,40E-14
7	0,72	0,65	48,79	1,49E-15	7,06	1,45E-02	52,07	8,84E-16
8	1,61	0,51	43,27	2,03E-14	10,95	4,06E-03	59,68	7,36E-17
9	3,22	0,45	32,36	8,40E-12	11,44	2,19E-03	49,24	2,84E-15
10	0,00	0,98	47,51	2,72E-15	4,13	5,60E-02	35,82	1,31E-12
11	0,11	0,88	72,12	8,76E-20	0,91	3,57E-01	21,33	1,41E-08
12	0,32	0,79	81,07	3,58E-21	4,93	3,71E-02	37,00	6,79E-13
13	0,93	0,63	65,72	8,62E-19	7,88	1,11E-02	36,71	7,88E-13
14	2,63	0,47	66,16	7,42E-19	9,65	5,80E-03	48,77	3,53E-15
15	1,15	0,58	52,10	3,02E-16	8,61	8,81E-03	51,64	1,02E-15
16	0,55	0,71	48,96	1,39E-15	7,94	1,11E-02	49,27	2,84E-15
17	0,54	0,71	39,71	1,29E-13	5,78	2,52E-02	38,17	3,68E-13
18	0,10	0,89	60,53	7,83E-18	0,83	3,75E-01	21,72	1,08E-08
19	0,13	0,87	81,94	3,58E-21	2,63	1,24E-01	27,33	2,31E-10
20	1,60	0,51	72,59	7,71E-20	7,93	1,11E-02	30,35	3,37E-11
21	3,01	0,45	70,81	1,37E-19	13,42	1,75E-03	42,66	3,78E-14
22	3,03	0,45	59,32	1,18E-17	11,30	2,19E-03	38,14	3,68E-13
23	0,12	0,88	46,87	3,76E-15	6,35	2,09E-02	34,41	2,89E-12
24	0,16	0,86	53,71	1,44E-16	5,69	2,62E-02	45,18	1,28E-14
25	0,83	0,63	59,73	1,03E-17	3,83	6,48E-02	16,87	3,88E-07
26	0,84	0,63	81,22	3,58E-21	2,44	1,37E-01	28,46	1,11E-10
27	0,65	0,67	84,54	2,03E-21	3,60	7,33E-02	27,47	2,12E-10
28	0,00	0,98	79,99	4,84E-21	6,20	2,17E-02	40,45	1,10E-13
29	0,18	0,85	68,17	3,53E-19	6,48	1,96E-02	47,63	4,81E-15
30	0,01	0,98	60,16	8,55E-18	6,12	2,17E-02	51,52	1,04E-15
31	0,15	0,86	56,20	4,60E-17	2,56	1,28E-01	16,43	5,39E-07
32	1,31	0,54	48,90	1,42E-15	0,75	3,99E-01	11,69	2,41E-05
33	0,02	0,98	81,72	3,58E-21	0,45	5,07E-01	23,13	3,99E-09
34	0,03	0,97	87,96	7,80E-22	3,30	8,51E-02	41,95	5,35E-14
35	0,03	0,97	76,44	1,75E-20	7,10	1,45E-02	49,36	2,84E-15
36	0,00	0,98	69,29	2,38E-19	5,52	2,84E-02	53,04	6,48E-16
37	0,12	0,88	59,59	1,06E-17	1,21	2,93E-01	29,77	4,83E-11
38	0,29	0,79	79,74	4,84E-21	2,07	1,70E-01	32,80	7,45E-12
39	0,02	0,97	93,01	2,21E-22	5,79	2,52E-02	60,83	5,22E-17
40	0,22	0,83	56,79	3,57E-17	3,32	8,51E-02	46,77	6,27E-15
41	0,26	0,81	55,88	5,28E-17	7,21	1,34E-02	39,58	1,71E-13

**42**	**0,50**	**0,72**	**45,59**	**6,70E-15**	**6,20**	**2,17E-02**	**32,05**	**1,17E-11**
**43**	**0,74**	**0,65**	**49,68**	**1,01E-15**	**6,59**	**1,83E-02**	**44,71**	**1,56E-14**
**44**	**1,22**	**0,56**	**45,02**	**8,90E-15**	**7,32**	**1,34E-02**	**32,43**	**9,36E-12**
**45**	**5,90**	**0,32**	**39,57**	**1,40E-13**	**11,56**	**2,19E-03**	**41,36**	**6,95E-14**
**46**	**0,16**	**0,86**	**47,84**	**2,34E-15**	**3,95**	**6,16E-02**	**35,66**	**1,42E-12**
**47**	**0,11**	**0,88**	**63,04**	**2,79E-18**	**8,49**	**8,81E-03**	**43,58**	**2,46E-14**
**48**	**0,01**	**0,98**	**60,23**	**8,55E-18**	**5,27**	**3,15E-02**	**40,59**	**1,02E-13**
**49**	**0,52**	**0,72**	**49,24**	**1,22E-15**	**8,57**	**8,81E-03**	**34,31**	**3,04E-12**
**50**	**3,09**	**0,45**	**35,20**	**1,65E-12**	**18,88**	**6,40E-04**	**28,83**	**8,75E-11**
**51**	**2,09**	**0,47**	**34,81**	**2,03E-12**	**16,86**	**8,02E-04**	**31,00**	**2,22E-11**
**52**	**1,39**	**0,54**	**29,67**	**4,64E-11**	**11,27**	**2,19E-03**	**26,25**	**4,76E-10**
**53**	**2,41**	**0,47**	**34,67**	**2,19E-12**	**5,08**	**3,48E-02**	**30,18**	**3,72E-11**
**54**	**0,86**	**0,63**	**56,55**	**3,93E-17**	**18,45**	**6,84E-04**	**42,55**	**3,91E-14**
**55**	**0,00**	**0,98**	**50,84**	**5,76E-16**	**6,03**	**2,29E-02**	**36,88**	**7,14E-13**
**56**	**0,25**	**0,81**	**46,50**	**4,42E-15**	**5,33**	**3,15E-02**	**33,63**	**4,57E-12**
**57**	**0,76**	**0,65**	**23,60**	**2,70E-09**	**11,80**	**2,19E-03**	**18,60**	**1,04E-07**
**58**	**1,81**	**0,49**	**24,19**	**1,80E-09**	**15,41**	**1,06E-03**	**19,96**	**3,78E-08**
**59**	**3,14**	**0,45**	**25,07**	**9,84E-10**	**16,96**	**8,02E-04**	**24,32**	**1,74E-09**
**60**	**1,29**	**0,54**	**29,12**	**6,61E-11**	**8,37**	**8,81E-03**	**28,84**	**8,70E-11**
**61**	**2,38**	**0,47**	**46,81**	**3,82E-15**	**17,49**	**8,02E-04**	**32,12**	**1,13E-11**
**62**	**1,34**	**0,54**	**42,84**	**2,50E-14**	**7,10**	**1,45E-02**	**38,31**	**3,45E-13**
**63**	**3,27**	**0,45**	**27,15**	**2,43E-10**	**8,73**	**8,81E-03**	**22,36**	**6,89E-09**
**64**	**2,12**	**0,47**	**22,36**	**6,45E-09**	**13,87**	**1,55E-03**	**22,32**	**7,02E-09**
**65**	**3,02**	**0,45**	**22,57**	**5,60E-09**	**13,60**	**1,68E-03**	**21,31**	**1,44E-08**
**66**	**2,45**	**0,47**	**22,57**	**5,60E-09**	**15,49**	**1,06E-03**	**21,14**	**1,61E-08**
**67**	**0,44**	**0,73**	**58,71**	**1,56E-17**	**10,20**	**4,06E-03**	**43,68**	**2,39E-14**
**68**	**1,13**	**0,58**	**42,74**	**2,62E-14**	**9,00**	**7,44E-03**	**34,95**	**2,09E-12**
**69**	**0,79**	**0,64**	**29,09**	**6,72E-11**	**15,15**	**1,12E-03**	**20,96**	**1,84E-08**
**70**	**0,46**	**0,73**	**24,71**	**1,26E-09**	**7,26**	**1,34E-02**	**24,56**	**1,48E-09**
**71**	**2,33**	**0,47**	**21,50**	**1,20E-08**	**12,21**	**2,19E-03**	**26,30**	**4,62E-10**
**72**	**2,11**	**0,47**	**23,97**	**2,10E-09**	**16,83**	**8,02E-04**	**25,80**	**6,43E-10**
**73**	**0,64**	**0,67**	**32,58**	**7,41E-12**	**5,55**	**2,84E-02**	**31,92**	**1,27E-11**
**74**	**1,64**	**0,51**	**26,57**	**3,59E-10**	**11,33**	**2,19E-03**	**28,46**	**1,11E-10**
**75**	**2,72**	**0,47**	**26,44**	**3,91E-10**	**15,65**	**1,06E-03**	**28,18**	**1,33E-10**
**76**	**1,47**	**0,53**	**25,37**	**8,05E-10**	**11,79**	**2,19E-03**	**24,87**	**1,21E-09**
**77**	**1,57**	**0,51**	**30,06**	**3,61E-11**	**13,24**	**1,87E-03**	**32,69**	**7,95E-12**
**78**	**2,50**	**0,47**	**42,24**	**3,38E-14**	**7,61**	**1,22E-02**	**31,46**	**1,66E-11**
**79**	**4,51**	**0,44**	**36,56**	**7,25E-13**	**9,01**	**7,44E-03**	**40,80**	**9,12E-14**
**80**	**5,64**	**0,32**	**33,04**	**5,73E-12**	**11,66**	**2,19E-03**	**39,51**	**1,76E-13**
**81**	**1,19**	**0,57**	**32,97**	**5,93E-12**	**13,87**	**1,55E-03**	**39,46**	**1,80E-13**
**82**	**0,99**	**0,61**	**33,52**	**4,29E-12**	**12,23**	**2,19E-03**	**35,96**	**1,21E-12**
**83**	**1,97**	**0,47**	**26,29**	**4,30E-10**	**14,56**	**1,31E-03**	**30,19**	**3,71E-11**
**84**	**3,83**	**0,45**	**27,25**	**2,29E-10**	**15,80**	**1,05E-03**	**31,77**	**1,37E-11**
**85**	**3,22**	**0,45**	**30,99**	**1,99E-11**	**14,81**	**1,21E-03**	**35,36**	**1,69E-12**
**86**	**3,24**	**0,45**	**45,61**	**6,70E-15**	**10,82**	**4,06E-03**	**40,86**	**9,04E-14**
**87**	**5,62**	**0,32**	**44,00**	**1,47E-14**	**14,32**	**1,39E-03**	**46,56**	**6,88E-15**
**88**	**6,83**	**0,31**	**43,57**	**1,77E-14**	**14,30**	**1,39E-03**	**53,33**	**6,14E-16**
**89**	**2,00**	**0,47**	**33,76**	**3,71E-12**	**15,74**	**1,05E-03**	**35,80**	**1,32E-12**
**90**	**1,37**	**0,54**	**34,52**	**2,38E-12**	**19,32**	**6,06E-04**	**45,87**	**9,38E-15**

**91**	**1,77**	**0,49**	**34,94**	**1,89E-12**	**14,81**	**1,21E-03**	**40,40**	**1,12E-13**
**92**	**1,98**	**0,47**	**33,13**	**5,45E-12**	**14,06**	**1,50E-03**	**42,63**	**3,80E-14**
**93**	**2,97**	**0,45**	**33,89**	**3,45E-12**	**20,28**	**5,57E-04**	**47,50**	**4,81E-15**
**94**	**3,12**	**0,45**	**37,02**	**5,71E-13**	**17,25**	**8,02E-04**	**44,05**	**2,05E-14**
**95**	**4,17**	**0,45**	**41,93**	**4,00E-14**	**16,46**	**8,51E-04**	**37,87**	**4,17E-13**
**96**	**10,87**	**0,20**	**38,53**	**2,43E-13**	**23,67**	**4,54E-04**	**39,59**	**1,71E-13**
**97**	**9,71**	**0,20**	**44,40**	**1,19E-14**	**24,73**	**4,25E-04**	**44,21**	**1,94E-14**
**98**	**2,30**	**0,47**	**38,56**	**2,43E-13**	**19,88**	**5,74E-04**	**41,04**	**8,31E-14**
**99**	**1,01**	**0,61**	**43,35**	**1,96E-14**	**16,44**	**8,51E-04**	**63,16**	**5,01E-17**
**100**	**0,71**	**0,65**	**34,29**	**2,72E-12**	**15,28**	**1,10E-03**	**46,15**	**8,45E-15**
**101**	**0,47**	**0,73**	**39,19**	**1,71E-13**	**11,30**	**2,19E-03**	**43,63**	**2,41E-14**
**102**	**2,00**	**0,47**	**34,55**	**2,36E-12**	**18,66**	**6,62E-04**	**41,70**	**5,88E-14**
**103**	**2,43**	**0,47**	**37,30**	**4,92E-13**	**17,37**	**8,02E-04**	**45,90**	**9,38E-15**
**104**	**7,30**	**0,31**	**32,54**	**7,55E-12**	**25,75**	**4,25E-04**	**39,91**	**1,45E-13**
**105**	**0,16**	**0,86**	**49,65**	**1,01E-15**	**15,96**	**1,01E-03**	**44,11**	**2,02E-14**
**106**	**0,23**	**0,82**	**46,54**	**4,37E-15**	**8,49**	**8,81E-03**	**45,53**	**1,08E-14**
**107**	**0,67**	**0,66**	**53,09**	**1,95E-16**	**1,04**	**3,28E-01**	**33,35**	**5,40E-12**
**108**	**0,07**	**0,93**	**40,79**	**7,38E-14**	**14,83**	**1,21E-03**	**49,64**	**2,70E-15**
**109**	**0,48**	**0,73**	**32,44**	**8,01E-12**	**16,90**	**8,02E-04**	**39,26**	**2,01E-13**
**110**	**2,16**	**0,47**	**32,65**	**7,16E-12**	**18,05**	**7,63E-04**	**34,45**	**2,83E-12**
**111**	**6,51**	**0,32**	**43,62**	**1,73E-14**	**22,66**	**4,54E-04**	**43,93**	**2,16E-14**
**112**	**0,76**	**0,65**	**42,09**	**3,66E-14**	**7,93**	**1,11E-02**	**44,22**	**1,94E-14**
**113**	**0,50**	**0,72**	**42,93**	**2,43E-14**	**9,78**	**5,80E-03**	**48,43**	**4,11E-15**
**114**	**0,45**	**0,73**	**35,13**	**1,70E-12**	**10,46**	**4,06E-03**	**45,12**	**1,31E-14**
**115**	**0,02**	**0,97**	**36,27**	**8,61E-13**	**16,83**	**8,02E-04**	**47,23**	**5,20E-15**
**116**	**1,56**	**0,51**	**32,62**	**7,27E-12**	**21,21**	**5,15E-04**	**52,19**	**8,84E-16**
**117**	**5,15**	**0,36**	**36,91**	**6,00E-13**	**20,96**	**5,15E-04**	**44,99**	**1,38E-14**
**118**	**5,72**	**0,32**	**37,12**	**5,38E-13**	**19,62**	**5,74E-04**	**42,33**	**4,40E-14**
**119**	**7,68**	**0,31**	**36,65**	**6,92E-13**	**21,47**	**5,15E-04**	**36,07**	**1,14E-12**
**120**	**7,01**	**0,31**	**46,26**	**4,91E-15**	**14,33**	**1,39E-03**	**47,55**	**4,81E-15**
**121**	**3,18**	**0,45**	**40,44**	**8,68E-14**	**7,86**	**1,11E-02**	**37,90**	**4,12E-13**
**122**	**4,67**	**0,44**	**38,24**	**2,87E-13**	**17,92**	**7,68E-04**	**47,43**	**4,81E-15**
**123**	**0,37**	**0,78**	**42,98**	**2,38E-14**	**12,21**	**2,19E-03**	**36,95**	**6,91E-13**
**124**	**0,29**	**0,79**	**40,48**	**8,64E-14**	**17,73**	**7,93E-04**	**48,01**	**4,66E-15**
**125**	**0,00**	**0,98**	**38,09**	**3,09E-13**	**21,61**	**5,15E-04**	**56,65**	**1,83E-16**
**126**	**0,34**	**0,78**	**36,72**	**6,68E-13**	**19,63**	**5,74E-04**	**56,29**	**1,97E-16**
**127**	**2,09**	**0,47**	**35,19**	**1,66E-12**	**20,70**	**5,17E-04**	**49,29**	**2,84E-15**
**128**	**2,36**	**0,47**	**41,62**	**4,68E-14**	**15,45**	**1,06E-03**	**47,15**	**5,31E-15**
**129**	**2,36**	**0,47**	**34,15**	**2,97E-12**	**9,63**	**5,80E-03**	**33,62**	**4,57E-12**
**130**	**5,71**	**0,32**	**41,15**	**6,04E-14**	**12,54**	**2,19E-03**	**37,10**	**6,43E-13**
**131**	**2,78**	**0,47**	**45,45**	**7,10E-15**	**7,92**	**1,11E-02**	**48,04**	**4,66E-15**
**132**	**1,50**	**0,52**	**41,75**	**4,37E-14**	**7,92**	**1,11E-02**	**42,14**	**4,83E-14**
**133**	**0,00**	**0,99**	**41,55**	**4,85E-14**	**22,66**	**4,54E-04**	**43,20**	**2,82E-14**
**134**	**0,01**	**0,98**	**43,77**	**1,63E-14**	**19,12**	**6,17E-04**	**62,37**	**5,01E-17**
**135**	**0,35**	**0,78**	**40,64**	**7,96E-14**	**14,02**	**1,50E-03**	**47,45**	**4,81E-15**
**136**	**1,68**	**0,51**	**44,98**	**9,00E-15**	**16,51**	**8,51E-04**	**57,45**	**1,38E-16**
**137**	**0,87**	**0,63**	**43,75**	**1,63E-14**	**11,44**	**2,19E-03**	**54,72**	**3,96E-16**
**138**	**0,80**	**0,64**	**46,19**	**5,05E-15**	**5,73**	**2,62E-02**	**47,88**	**4,81E-15**
**139**	**2,08**	**0,47**	**40,24**	**9,71E-14**	**8,02**	**1,04E-02**	**43,40**	**2,54E-14**

140	2,48	0,47	38,15	3,01E-13	7,86	1,11E-02	41,76	5,81E-14
141	4,05	0,45	30,75	2,31E-11	12,07	2,19E-03	38,05	3,84E-13
142	0,01	0,98	44,50	1,14E-14	17,23	8,02E-04	33,14	6,09E-12
143	0,00	0,98	43,51	1,81E-14	12,85	2,18E-03	44,68	1,56E-14
144	0,29	0,79	48,04	2,19E-15	11,23	2,19E-03	59,19	7,79E-17
145	1,76	0,49	55,77	5,46E-17	16,80	8,02E-04	61,05	5,22E-17
146	0,97	0,62	45,51	6,91E-15	7,65	1,22E-02	45,85	9,38E-15
147	1,33	0,54	46,03	5,45E-15	5,35	3,06E-02	38,86	2,51E-13
148	3,08	0,45	33,80	3,65E-12	12,00	2,19E-03	36,57	8,51E-13
149	4,01	0,45	35,05	1,78E-12	12,35	2,19E-03	25,70	6,84E-10
150	0,03	0,97	42,91	2,43E-14	12,23	2,19E-03	34,96	2,09E-12
151	0,01	0,98	50,51	6,72E-16	7,27	1,34E-02	43,47	2,51E-14
152	0,91	0,63	62,66	3,12E-18	7,88	1,11E-02	52,01	8,84E-16
153	1,39	0,54	49,24	1,22E-15	8,99	7,44E-03	46,99	5,67E-15
154	2,37	0,47	43,89	1,55E-14	11,50	2,19E-03	38,27	3,47E-13
155	2,24	0,47	38,53	2,43E-13	12,36	2,19E-03	34,37	2,94E-12
156	4,11	0,45	42,71	2,65E-14	8,20	1,04E-02	31,85	1,31E-11
157	0,43	0,74	58,42	1,75E-17	10,88	4,06E-03	37,81	4,28E-13
158	0,82	0,64	60,21	8,55E-18	9,34	5,80E-03	43,44	2,52E-14
159	2,03	0,47	47,69	2,50E-15	11,81	2,19E-03	40,97	8,55E-14
160	1,88	0,48	47,87	2,33E-15	13,79	1,58E-03	36,96	6,91E-13
161	4,05	0,45	38,53	2,43E-13	13,55	1,69E-03	29,40	6,14E-11
162	3,49	0,45	32,80	6,56E-12	15,42	1,06E-03	25,60	7,32E-10
163	3,85	0,45	34,69	2,18E-12	12,51	2,19E-03	23,27	3,63E-09
164	5,15	0,36	38,80	2,14E-13	11,18	2,19E-03	34,95	2,09E-12
165	2,51	0,47	40,44	8,68E-14	7,47	1,22E-02	43,51	2,48E-14
166	1,57	0,51	43,88	1,55E-14	6,68	1,73E-02	43,87	2,20E-14
167	2,24	0,47	32,97	5,93E-12	11,57	2,19E-03	38,13	3,68E-13
168	0,58	0,70	58,14	1,96E-17	9,83	5,80E-03	34,02	3,62E-12
169	0,87	0,63	46,39	4,64E-15	10,91	4,06E-03	39,74	1,58E-13
170	1,82	0,49	39,41	1,52E-13	15,13	1,12E-03	29,31	6,45E-11
171	1,01	0,61	40,70	7,71E-14	7,95	1,11E-02	29,31	6,45E-11
172	0,99	0,61	30,99	1,99E-11	7,60	1,22E-02	21,65	1,13E-08
173	3,48	0,45	37,22	5,09E-13	11,37	2,19E-03	26,76	3,40E-10
174	3,54	0,45	31,66	1,30E-11	10,50	4,06E-03	24,81	1,25E-09
175	2,87	0,47	37,34	4,81E-13	4,36	4,92E-02	37,43	5,35E-13
176	1,66	0,51	39,72	1,29E-13	6,54	1,83E-02	38,37	3,34E-13
177	1,34	0,54	42,30	3,30E-14	8,80	7,44E-03	43,85	2,20E-14
178	0,34	0,78	48,00	2,21E-15	7,76	1,11E-02	31,90	1,28E-11
179	0,08	0,91	39,15	1,75E-13	2,11	1,66E-01	20,12	3,35E-08
180	0,22	0,83	45,93	5,68E-15	3,03	9,89E-02	33,14	6,09E-12
181	1,98	0,47	40,02	1,10E-13	5,12	3,48E-02	35,37	1,69E-12
182	0,90	0,63	44,51	1,14E-14	4,66	4,22E-02	40,84	9,05E-14
183	1,87	0,48	52,45	2,61E-16	6,18	2,17E-02	47,60	4,81E-15
184	1,04	0,61	52,80	2,22E-16	5,37	3,06E-02	54,05	4,75E-16
185	0,29	0,79	57,60	2,44E-17	3,75	6,79E-02	35,08	1,99E-12
186	0,00	0,98	52,13	3,02E-16	1,74	2,05E-01	37,98	3,96E-13
187	0,09	0,89	44,84	9,59E-15	0,46	5,04E-01	34,97	2,09E-12
188	0,04	0,97	53,70	1,44E-16	1,10	3,15E-01	44,32	1,88E-14

189	0,00	0,99	54,26	1,15E-16	2,22	1,56E-01	47,42	4,81E-15
190	0,03	0,97	53,98	1,30E-16	3,01	9,95E-02	47,40	4,81E-15
191	0,01	0,98	67,76	4,04E-19	4,88	3,81E-02	48,12	4,66E-15
192	0,02	0,97	68,48	3,23E-19	2,05	1,71E-01	54,45	4,18E-16
193	0,20	0,83	46,89	3,76E-15	0,88	3,64E-01	41,73	5,83E-14
194	0,00	0,98	57,61	2,44E-17	1,74	2,05E-01	41,78	5,80E-14
195	1,35	0,54	66,66	6,39E-19	0,19	6,62E-01	41,45	6,72E-14
196	0,14	0,87	62,78	3,05E-18	4,86	3,88E-02	47,69	4,81E-15
197	0,06	0,93	63,10	2,79E-18	3,15	9,26E-02	47,56	4,81E-15
198	2,30	0,47	71,17	1,24E-19	12,58	2,19E-03	43,51	2,48E-14
199	9,49	0,20	36,97	5,85E-13	25,65	4,25E-04	53,27	6,14E-16

Supplementary Table 4 Statistical values of ANOVAs assessing group and period effect on aperiodic parameters at the cortex level.

	EXPONENT				OFFSET			
	group		period		group		period	
ROI	F	P	F	p	F	p	F	p
1	0,72	0,74	25,79	7,01E-10	5,51	0,05	13,75	4,48E-06
2	4,35	0,42	59,02	5,78E-18	6,94	0,04	14,14	3,31E-06
3	2,63	0,42	64,05	6,71E-19	4,75	0,06	47,11	5,58E-15
4	3,17	0,42	55,31	3,24E-17	4,73	0,06	36,83	1,42E-12
5	1,14	0,67	76,61	3,70E-21	2,75	0,14	29,95	6,63E-11
6	0,89	0,70	73,80	1,10E-20	0,88	0,40	28,43	1,63E-10
7	1,47	0,60	12,07	1,79E-05	7,81	0,03	24,84	1,57E-09
8	0,75	0,74	16,99	3,71E-07	9,04	0,02	29,07	1,09E-10
9	5,10	0,42	58,84	6,12E-18	11,65	0,02	43,81	3,06E-14
10	0,05	0,95	41,41	4,89E-14	5,20	0,05	46,79	6,21E-15
11	0,79	0,73	29,56	6,05E-11	0,00	1,00	48,12	3,42E-15
12	0,96	0,68	38,46	2,70E-13	0,00	0,97	48,57	2,90E-15
13	0,05	0,95	15,15	1,50E-06	9,50	0,02	20,60	2,71E-08
14	0,00	1,00	19,07	7,98E-08	6,52	0,04	20,86	2,31E-08
15	3,96	0,42	33,74	4,46E-12	11,37	0,02	14,56	2,45E-06
16	5,92	0,42	50,62	3,40E-16	5,94	0,05	14,97	1,80E-06
17	1,62	0,59	27,87	1,80E-10	10,53	0,02	24,61	1,78E-09
18	0,35	0,84	60,30	3,33E-18	5,62	0,05	26,50	5,49E-10
19	2,98	0,42	56,16	2,25E-17	4,78	0,06	35,93	2,25E-12
20	1,05	0,67	86,44	1,08E-22	2,35	0,17	31,64	2,57E-11
21	0,02	0,97	36,93	6,69E-13	8,15	0,03	30,41	5,07E-11
22	0,49	0,79	36,10	1,08E-12	7,24	0,04	21,42	1,63E-08
23	0,30	0,84	20,51	2,80E-08	6,82	0,04	20,97	2,18E-08
24	0,66	0,74	27,27	2,64E-10	9,04	0,02	18,70	1,02E-07
25	0,41	0,82	60,40	3,27E-18	5,60	0,05	57,80	5,27E-17
26	0,29	0,84	62,61	1,22E-18	4,30	0,07	43,05	4,22E-14

27	0,25	0,86	16,78	4,30E-07	4,46	0,07	25,29	1,20E-09
28	0,17	0,91	22,75	5,69E-09	4,39	0,07	21,50	1,57E-08
29	0,00	1,00	55,60	2,88E-17	2,63	0,15	57,86	5,27E-17
30	0,00	1,00	54,36	5,13E-17	2,20	0,18	54,82	1,48E-16
31	2,16	0,50	76,92	3,48E-21	8,54	0,03	31,14	3,34E-11
32	1,07	0,67	78,81	1,82E-21	7,00	0,04	29,14	1,09E-10
33	2,96	0,42	25,96	6,34E-10	6,27	0,04	20,79	2,40E-08
34	1,03	0,67	31,15	2,23E-11	3,16	0,11	18,70	1,02E-07
35	0,63	0,74	30,34	3,71E-11	8,57	0,03	31,80	2,40E-11
36	0,04	0,95	49,66	5,51E-16	6,28	0,04	32,69	1,41E-11
37	0,02	0,97	62,70	1,22E-18	0,37	0,59	39,90	2,38E-13
38	0,02	0,97	110,52	7,00E-26	0,15	0,73	56,28	8,45E-17
39	0,19	0,90	101,66	7,74E-25	3,32	0,11	34,39	5,39E-12
40	0,06	0,95	61,62	1,89E-18	1,30	0,30	43,31	3,85E-14
41	0,29	0,84	94,43	7,38E-24	0,50	0,54	36,03	2,21E-12
42	0,06	0,95	119,79	6,50E-27	0,28	0,63	51,72	5,84E-16
43	0,06	0,95	11,59	2,61E-05	5,35	0,05	26,23	6,42E-10
44	0,07	0,95	17,93	1,82E-07	4,74	0,06	22,61	7,13E-09
45	3,32	0,42	49,37	6,26E-16	5,78	0,05	20,42	3,03E-08
46	2,82	0,42	100,12	1,08E-24	1,66	0,24	28,17	1,89E-10
47	2,39	0,46	34,37	3,09E-12	5,79	0,05	19,55	5,68E-08
48	3,01	0,42	51,74	1,95E-16	5,76	0,05	30,50	4,90E-11
49	3,40	0,42	66,58	2,26E-19	2,81	0,13	21,35	1,67E-08
50	1,11	0,67	89,80	3,71E-23	1,39	0,28	31,21	3,29E-11
51	0,54	0,77	28,84	9,60E-11	11,20	0,02	29,09	1,09E-10
52	1,51	0,60	25,54	8,17E-10	10,11	0,02	32,93	1,25E-11
53	0,09	0,95	68,27	1,22E-19	2,06	0,19	74,72	6,26E-20
54	0,00	1,00	85,43	1,46E-22	3,35	0,11	82,62	4,53E-21
55	0,40	0,82	76,99	3,48E-21	0,47	0,55	60,40	2,21E-17
56	0,61	0,74	87,93	6,73E-23	0,02	0,92	61,09	2,11E-17
57	0,00	1,00	102,01	7,74E-25	2,97	0,12	56,53	8,45E-17
58	0,08	0,95	74,91	7,23E-21	2,25	0,18	40,99	1,32E-13
59	3,99	0,42	18,94	8,69E-08	10,44	0,02	24,76	1,64E-09
60	2,74	0,42	21,88	1,04E-08	3,98	0,08	19,17	7,42E-08
61	3,05	0,42	45,21	5,80E-15	9,80	0,02	33,60	8,51E-12
62	1,82	0,56	66,24	2,53E-19	6,46	0,04	34,80	4,32E-12
63	6,18	0,42	31,24	2,15E-11	3,98	0,08	14,38	2,77E-06
64	5,87	0,42	84,60	1,84E-22	4,21	0,07	25,09	1,35E-09
65	1,76	0,56	67,16	1,91E-19	6,24	0,04	54,73	1,48E-16
66	1,84	0,56	59,22	5,42E-18	8,79	0,02	53,39	2,69E-16
67	1,41	0,60	46,13	3,55E-15	8,05	0,03	26,85	4,52E-10
68	2,72	0,42	66,61	2,26E-19	4,70	0,06	26,67	5,00E-10

Supplementary Table 5 Statistical values of post-hoc t-tests comparing the aperiodic exponent distribution according to the task period at the scalp level.

	post-pre		post-rest		pre-rest	
**Electrode**	**t**	**p**	**t**	**p**	**t**	**p**
**1**	5,94	2,81E-06	-6,07	1,36E-06	-11,56	3,28E-15
**2**	4,98	3,56E-05	-6,40	4,63E-07	-10,81	3,08E-14
**3**	3,61	2,19E-03	-8,03	6,65E-09	-12,57	1,66E-16
**4**	2,99	1,21E-02	-7,39	3,80E-08	-12,30	3,40E-16
**5**	4,01	6,77E-04	-6,53	3,20E-07	-10,83	2,99E-14
**6**	4,17	4,15E-04	-5,48	8,31E-06	-10,30	1,51E-13
**7**	5,33	1,36E-05	-4,51	1,61E-04	-10,22	1,87E-13
**8**	6,43	9,85E-07	-3,44	3,78E-03	-9,38	2,61E-12
**9**	5,47	9,09E-06	-2,91	1,70E-02	-8,52	4,52E-11
**10**	4,18	4,10E-04	-5,47	8,61E-06	-10,54	7,19E-14
**11**	4,19	3,94E-04	-7,05	8,04E-08	-13,94	7,23E-18
**12**	2,93	1,50E-02	-8,93	1,01E-09	-14,22	4,33E-18
**13**	3,95	7,95E-04	-6,95	1,00E-07	-11,57	3,28E-15
**14**	5,35	1,31E-05	-5,90	2,19E-06	-12,03	7,03E-16
**15**	5,29	1,47E-05	-4,95	4,10E-05	-10,23	1,87E-13
**16**	5,34	1,36E-05	-4,75	7,88E-05	-9,59	1,30E-12
**17**	6,35	1,14E-06	-3,23	7,28E-03	-8,96	1,03E-11
**18**	4,76	6,66E-05	-6,54	3,19E-07	-10,78	3,24E-14
**19**	4,16	4,22E-04	-8,35	4,72E-09	-12,70	1,16E-16
**20**	4,42	1,97E-04	-6,78	1,71E-07	-13,45	2,66E-17
**21**	5,29	1,49E-05	-6,22	8,59E-07	-12,53	1,75E-16
**22**	5,45	9,68E-06	-5,39	1,05E-05	-11,19	1,06E-14
**23**	4,68	8,38E-05	-4,68	9,44E-05	-10,39	1,11E-13
**24**	5,72	4,51E-06	-4,83	5,97E-05	-10,48	8,33E-14
**25**	5,22	1,79E-05	-5,83	2,72E-06	-10,67	4,74E-14
**26**	5,14	2,26E-05	-7,09	7,72E-08	-12,91	7,03E-17
**27**	5,83	3,67E-06	-7,04	8,04E-08	-13,21	3,98E-17
**28**	5,86	3,34E-06	-6,77	1,71E-07	-12,45	2,13E-16
**29**	4,81	5,87E-05	-6,59	2,83E-07	-12,20	4,33E-16
**30**	5,33	1,36E-05	-5,47	8,54E-06	-11,47	4,24E-15
**31**	5,19	1,93E-05	-5,55	6,63E-06	-9,90	5,17E-13
**32**	4,82	5,71E-05	-4,98	3,85E-05	-9,93	4,64E-13
**33**	5,66	5,01E-06	-7,15	7,04E-08	-12,74	1,14E-16
**34**	5,61	5,84E-06	-7,42	3,80E-08	-13,20	3,98E-17
**35**	4,27	3,11E-04	-7,55	2,96E-08	-13,05	4,98E-17
**36**	4,96	3,79E-05	-6,49	3,56E-07	-12,13	5,34E-16
**37**	6,49	8,91E-07	-4,92	4,51E-05	-10,59	6,03E-14
**38**	5,22	1,79E-05	-7,10	7,72E-08	-13,09	4,98E-17
**39**	5,02	3,19E-05	-8,06	6,65E-09	-14,73	1,75E-18
**40**	4,25	3,33E-04	-6,02	1,59E-06	-10,98	1,88E-14
**41**	5,29	1,47E-05	-5,38	1,12E-05	-10,48	8,33E-14
**42**	4,84	5,43E-05	-4,83	6,03E-05	-9,46	1,95E-12
**43**	5,45	9,80E-06	-4,60	1,21E-04	-10,10	2,69E-13
**44**	6,52	8,73E-07	-3,47	3,78E-03	-8,94	1,10E-11
**45**	6,21	1,35E-06	-2,69	3,10E-02	-9,58	1,30E-12

**46**	6,69	5,67E-07	-3,34	4,98E-03	-9,63	1,14E-12
**47**	6,26	1,33E-06	-5,20	2,00E-05	-11,17	1,10E-14
**48**	6,29	1,30E-06	-4,95	4,10E-05	-10,78	3,24E-14
**49**	5,38	1,21E-05	-5,01	3,48E-05	-9,33	3,09E-12
**50**	5,33	1,36E-05	-3,61	2,62E-03	-7,74	6,56E-10
**51**	5,77	4,02E-06	-2,94	1,60E-02	-7,67	8,57E-10
**52**	5,67	4,99E-06	-2,41	6,17E-02	-7,26	3,88E-09
**53**	6,02	2,21E-06	-2,46	5,49E-02	-8,26	1,10E-10
**54**	6,44	9,85E-07	-4,48	1,78E-04	-10,17	2,19E-13
**55**	5,81	3,70E-06	-4,50	1,64E-04	-9,87	5,39E-13
**56**	5,28	1,51E-05	-5,06	3,11E-05	-8,78	1,88E-11
**57**	3,97	7,66E-04	-3,33	6,14E-03	-6,22	1,83E-07
**58**	5,19	1,93E-05	-2,22	9,48E-02	-6,32	1,25E-07
**59**	5,98	2,52E-06	-1,68	2,99E-01	-5,97	4,61E-07
**60**	6,76	5,67E-07	-1,29	6,09E-01	-6,42	8,56E-08
**61**	6,08	1,86E-06	-3,58	2,62E-03	-9,12	6,53E-12
**62**	5,12	2,35E-05	-4,42	2,10E-04	-8,77	1,93E-11
**63**	4,10	5,08E-04	-3,79	1,36E-03	-6,68	3,31E-08
**64**	3,41	4,21E-03	-3,60	2,62E-03	-6,15	2,40E-07
**65**	4,78	6,17E-05	-2,18	1,04E-01	-5,87	6,81E-07
**66**	4,96	3,79E-05	-1,79	2,38E-01	-6,07	3,25E-07
**67**	5,90	3,11E-06	-5,44	9,20E-06	-9,87	5,39E-13
**68**	5,72	4,51E-06	-3,89	1,10E-03	-8,40	6,58E-11
**69**	4,06	5,74E-04	-4,05	6,91E-04	-6,86	1,70E-08
**70**	3,71	1,12E-03	-3,98	8,60E-04	-6,11	2,71E-07
**71**	3,93	8,27E-04	-3,21	7,28E-03	-5,80	8,55E-07
**72**	4,48	1,67E-04	-2,69	3,10E-02	-6,25	1,68E-07
**73**	3,49	3,21E-03	-5,41	1,02E-05	-6,85	1,72E-08
**74**	4,30	2,90E-04	-4,11	5,79E-04	-5,97	4,60E-07
**75**	3,96	7,75E-04	-3,65	2,62E-03	-6,67	3,32E-08
**76**	4,79	6,17E-05	-2,32	7,52E-02	-6,60	4,29E-08
**77**	6,09	1,86E-06	-1,55	3,80E-01	-7,24	4,09E-09
**78**	7,09	3,85E-07	-2,31	7,79E-02	-7,94	3,17E-10
**79**	6,35	1,14E-06	-2,44	5,78E-02	-8,34	8,38E-11
**80**	5,77	4,02E-06	-2,23	9,43E-02	-8,12	1,74E-10
**81**	3,14	8,16E-03	-6,08	1,35E-06	-6,77	2,31E-08
**82**	3,29	5,21E-03	-5,95	1,88E-06	-6,81	1,98E-08
**83**	3,28	5,21E-03	-4,28	3,29E-04	-6,78	2,22E-08
**84**	3,96	7,66E-04	-3,52	3,78E-03	-7,18	5,20E-09
**85**	5,21	1,79E-05	-2,55	4,35E-02	-8,22	1,24E-10
**86**	7,03	3,85E-07	-3,11	1,08E-02	-9,33	3,05E-12
**87**	6,68	5,67E-07	-3,08	1,18E-02	-8,75	2,01E-11
**88**	4,52	1,46E-04	-4,72	8,35E-05	-9,66	1,07E-12
**89**	3,67	2,19E-03	-5,44	9,24E-06	-6,95	1,20E-08
**90**	3,17	7,22E-03	-6,48	3,65E-07	-6,87	1,61E-08
**91**	3,15	8,16E-03	-6,22	8,59E-07	-7,03	8,82E-09
**92**	3,34	4,21E-03	-5,99	1,72E-06	-7,11	6,63E-09
**93**	4,21	3,81E-04	-4,37	2,50E-04	-7,60	1,10E-09
**94**	5,96	2,60E-06	-3,05	1,18E-02	-8,19	1,35E-10
**95**	7,20	3,85E-07	-1,84	2,15E-01	-9,07	7,44E-12
**96**	6,67	5,67E-07	-2,79	2,46E-02	-7,88	3,98E-10
**97**	5,68	4,90E-06	-4,12	5,56E-04	-9,05	7,91E-12
**98**	3,78	1,12E-03	-6,22	8,59E-07	-7,49	1,63E-09

**99**	3,36	4,21E-03	-7,32	4,30E-08	-8,05	2,22E-10
**100**	3,43	3,21E-03	-6,19	9,38E-07	-7,10	6,87E-09
**101**	3,80	1,12E-03	-6,01	1,63E-06	-7,70	7,61E-10
**102**	4,32	2,69E-04	-4,74	8,03E-05	-7,18	5,19E-09
**103**	6,02	2,21E-06	-2,92	1,70E-02	-8,42	6,28E-11
**104**	5,87	3,22E-06	-2,20	9,85E-02	-8,16	1,52E-10
**105**	3,83	1,11E-03	-7,29	4,45E-08	-8,78	1,88E-11
**106**	4,34	2,55E-04	-6,95	1,00E-07	-8,11	1,75E-10
**107**	4,97	3,68E-05	-6,60	2,82E-07	-8,96	1,02E-11
**108**	5,88	3,22E-06	-4,25	3,66E-04	-7,51	1,54E-09
**109**	6,67	5,67E-07	-1,86	2,09E-01	-6,70	2,99E-08
**110**	5,60	5,84E-06	-2,51	4,76E-02	-7,60	1,09E-09
**111**	6,68	5,67E-07	-3,27	6,14E-03	-8,68	2,60E-11
**112**	4,95	3,87E-05	-5,10	2,63E-05	-8,01	2,53E-10
**113**	4,82	5,70E-05	-5,82	2,80E-06	-7,78	5,65E-10
**114**	4,76	6,66E-05	-4,63	1,13E-04	-7,06	7,81E-09
**115**	6,36	1,14E-06	-2,93	1,60E-02	-7,24	4,09E-09
**116**	6,16	1,48E-06	-2,57	4,18E-02	-6,89	1,50E-08
**117**	6,22	1,33E-06	-3,03	1,29E-02	-7,63	9,71E-10
**118**	5,72	4,51E-06	-3,34	4,98E-03	-8,30	9,65E-11
**119**	5,81	3,70E-06	-3,00	1,39E-02	-8,68	2,59E-11
**120**	5,81	3,70E-06	-3,69	2,62E-03	-10,30	1,51E-13
**121**	5,40	1,11E-05	-3,90	1,10E-03	-8,83	1,58E-11
**122**	4,97	3,73E-05	-3,95	9,28E-04	-8,84	1,53E-11
**123**	4,91	4,26E-05	-5,15	2,31E-05	-8,09	1,92E-10
**124**	5,22	1,79E-05	-4,37	2,51E-04	-7,77	5,97E-10
**125**	5,59	6,16E-06	-3,80	1,36E-03	-7,38	2,45E-09
**126**	5,88	3,22E-06	-3,49	3,78E-03	-7,16	5,52E-09
**127**	6,26	1,33E-06	-2,99	1,39E-02	-7,29	3,49E-09
**128**	5,63	5,54E-06	-3,87	1,20E-03	-8,71	2,36E-11
**129**	5,13	2,29E-05	-2,86	2,02E-02	-8,59	3,60E-11
**130**	5,72	4,51E-06	-3,03	1,29E-02	-9,89	5,18E-13
**131**	6,19	1,40E-06	-3,63	2,62E-03	-9,20	4,92E-12
**132**	5,30	1,47E-05	-3,98	8,51E-04	-9,06	7,79E-12
**133**	4,99	3,54E-05	-4,60	1,21E-04	-7,87	4,03E-10
**134**	5,51	7,96E-06	-4,41	2,18E-04	-7,88	4,01E-10
**135**	4,80	5,92E-05	-4,65	1,06E-04	-7,54	1,34E-09
**136**	5,05	2,89E-05	-4,91	4,51E-05	-8,12	1,74E-10
**137**	6,24	1,33E-06	-4,25	3,63E-04	-7,80	5,21E-10
**138**	5,65	5,16E-06	-4,64	1,10E-04	-8,56	3,91E-11
**139**	5,68	4,90E-06	-3,50	3,78E-03	-8,50	4,74E-11
**140**	6,17	1,48E-06	-3,15	9,65E-03	-8,26	1,10E-10
**141**	4,13	4,67E-04	-3,80	1,36E-03	-7,63	9,84E-10
**142**	4,81	5,76E-05	-5,28	1,56E-05	-8,17	1,46E-10
**143**	5,12	2,35E-05	-5,02	3,48E-05	-7,89	3,83E-10
**144**	3,92	8,37E-04	-6,45	3,99E-07	-8,48	5,10E-11
**145**	5,32	1,41E-05	-6,74	1,81E-07	-8,45	5,61E-11
**146**	4,88	4,69E-05	-5,24	1,72E-05	-8,57	3,74E-11
**147**	4,84	5,37E-05	-4,94	4,22E-05	-9,25	4,11E-12
**148**	4,51	1,50E-04	-3,83	1,32E-03	-7,90	3,80E-10
**149**	4,77	6,47E-05	-3,98	8,60E-04	-7,95	3,15E-10
**150**	4,70	7,91E-05	-5,19	2,05E-05	-8,00	2,64E-10
**151**	4,11	4,84E-04	-6,94	1,01E-07	-8,56	3,91E-11

**152**	4,13	4,67E-04	-8,08	6,65E-09	-9,57	1,33E-12
**153**	3,82	1,12E-03	-6,73	1,84E-07	-8,97	1,01E-11
**154**	3,48	3,21E-03	-5,59	6,03E-06	-9,68	1,02E-12
**155**	3,94	8,15E-04	-4,80	6,60E-05	-8,58	3,74E-11
**156**	4,17	4,17E-04	-5,16	2,25E-05	-9,03	8,39E-12
**157**	4,43	1,93E-04	-7,52	2,99E-08	-8,97	1,02E-11
**158**	4,36	2,37E-04	-7,94	7,69E-09	-9,76	7,72E-13
**159**	3,93	8,27E-04	-6,49	3,56E-07	-8,99	9,55E-12
**160**	3,68	2,19E-03	-5,98	1,72E-06	-9,11	6,55E-12
**161**	3,72	1,12E-03	-5,17	2,19E-05	-8,25	1,15E-10
**162**	3,81	1,12E-03	-4,28	3,30E-04	-8,20	1,35E-10
**163**	4,50	1,52E-04	-3,95	9,30E-04	-8,18	1,41E-10
**164**	4,93	4,15E-05	-4,44	1,99E-04	-8,03	2,33E-10
**165**	4,23	3,59E-04	-4,96	4,02E-05	-8,66	2,79E-11
**166**	5,23	1,78E-05	-4,57	1,35E-04	-8,89	1,30E-11
**167**	6,65	5,67E-07	-2,56	4,26E-02	-7,49	1,65E-09
**168**	4,91	4,27E-05	-6,72	1,84E-07	-9,83	6,33E-13
**169**	3,95	7,81E-04	-5,97	1,83E-06	-9,13	6,33E-12
**170**	3,25	6,22E-03	-5,63	5,39E-06	-8,09	1,89E-10
**171**	3,05	1,02E-02	-5,89	2,22E-06	-8,75	2,01E-11
**172**	3,42	3,21E-03	-5,00	3,57E-05	-7,08	7,34E-09
**173**	3,54	2,19E-03	-5,50	8,08E-06	-8,13	1,66E-10
**174**	3,85	1,06E-03	-4,55	1,43E-04	-7,48	1,67E-09
**175**	3,76	1,12E-03	-5,05	3,11E-05	-8,23	1,23E-10
**176**	4,37	2,36E-04	-4,79	6,82E-05	-8,50	4,74E-11
**177**	5,78	3,95E-06	-3,75	1,36E-03	-9,08	7,21E-12
**178**	5,07	2,76E-05	-4,96	4,02E-05	-9,56	1,36E-12
**179**	2,93	1,41E-02	-5,76	3,35E-06	-8,41	6,38E-11
**180**	4,18	4,10E-04	-5,76	3,36E-06	-8,84	1,53E-11
**181**	4,39	2,20E-04	-5,11	2,63E-05	-8,21	1,27E-10
**182**	4,23	3,57E-04	-5,56	6,55E-06	-8,97	1,02E-11
**183**	4,57	1,25E-04	-5,92	2,07E-06	-9,64	1,14E-12
**184**	5,07	2,70E-05	-5,36	1,18E-05	-10,16	2,25E-13
**185**	5,69	4,90E-06	-5,77	3,34E-06	-9,75	7,96E-13
**186**	5,13	2,29E-05	-5,46	8,91E-06	-9,63	1,14E-12
**187**	4,52	1,45E-04	-5,05	3,16E-05	-9,56	1,36E-12
**188**	4,75	6,70E-05	-6,08	1,35E-06	-9,63	1,14E-12
**189**	3,96	7,75E-04	-6,55	3,18E-07	-9,98	4,02E-13
**190**	3,97	7,61E-04	-6,17	1,00E-06	-10,14	2,35E-13
**191**	6,33	1,17E-06	-5,60	5,94E-06	-11,05	1,47E-14
**192**	6,23	1,33E-06	-5,95	1,88E-06	-11,07	1,46E-14
**193**	5,14	2,28E-05	-4,59	1,25E-04	-9,77	7,72E-13
**194**	4,20	3,91E-04	-7,05	8,04E-08	-10,22	1,87E-13
**195**	3,65	2,19E-03	-7,36	3,91E-08	-11,44	4,56E-15
**196**	3,74	1,12E-03	-6,77	1,71E-07	-10,84	2,90E-14
**197**	6,71	5,67E-07	-5,26	1,62E-05	-10,00	3,80E-13
**198**	7,28	3,85E-07	-4,71	8,60E-05	-11,08	1,42E-14
**199**	5,25	1,64E-05	-3,46	3,78E-03	-8,75	2,07E-11

Supplementary Table 6 Statistical values of post-hoc t-tests comparing the aperiodic offset distribution according to the task period at the scalp level.

	post-pre		post-rest		rest-pre	
Electrode	t	p	t	p	t	p
1	7,60	2,85E-08	-3,93	1,63E-03	-10,14	5,94E-13
2	6,26	7,16E-07	-3,40	7,24E-03	-8,86	2,03E-11
3	5,80	2,36E-06	-4,21	8,30E-04	-10,40	3,65E-13
4	4,10	4,12E-04	-4,18	8,53E-04	-10,19	5,94E-13
5	4,19	3,12E-04	-4,47	4,93E-04	-9,40	4,93E-12
6	3,90	7,79E-04	-5,10	9,87E-05	-10,39	3,65E-13
7	4,55	1,00E-04	-5,14	9,48E-05	-11,32	5,07E-14
8	6,23	7,28E-07	-4,71	2,50E-04	-11,56	5,07E-14
9	5,67	3,43E-06	-4,32	6,38E-04	-10,44	3,65E-13
10	5,48	6,27E-06	-2,37	7,86E-02	-8,74	2,72E-11
11	4,86	3,84E-05	-1,03	9,65E-01	-6,45	7,99E-08
12	4,96	3,02E-05	-3,33	8,73E-03	-9,14	9,34E-12
13	4,69	6,58E-05	-3,68	3,94E-03	-8,76	2,59E-11
14	5,10	1,92E-05	-4,72	2,50E-04	-10,13	5,94E-13
15	4,90	3,50E-05	-5,18	8,66E-05	-10,40	3,65E-13
16	4,46	1,31E-04	-5,35	8,32E-05	-10,19	5,94E-13
17	4,90	3,53E-05	-3,96	1,52E-03	-8,62	3,89E-11
18	5,72	2,94E-06	-0,32	1,00E+00	-5,73	1,15E-06
19	5,13	1,80E-05	-1,86	2,36E-01	-7,25	3,98E-09
20	4,94	3,08E-05	-2,52	5,74E-02	-8,78	2,49E-11
21	5,23	1,33E-05	-3,77	2,21E-03	-9,95	9,52E-13
22	4,46	1,31E-04	-4,18	8,53E-04	-9,23	7,55E-12
23	3,62	2,00E-03	-4,42	5,27E-04	-9,17	9,02E-12
24	4,75	5,35E-05	-5,18	8,66E-05	-9,00	1,38E-11
25	5,21	1,41E-05	0,04	1,00E+00	-5,20	8,24E-06
26	5,58	4,67E-06	-1,62	3,68E-01	-7,69	8,28E-10
27	5,24	1,28E-05	-1,79	2,71E-01	-7,92	3,77E-10
28	5,69	3,20E-06	-3,39	7,24E-03	-9,25	7,25E-12
29	5,29	1,14E-05	-4,32	6,38E-04	-10,17	5,94E-13
30	5,37	8,72E-06	-4,79	2,12E-04	-10,33	4,27E-13
31	5,26	1,21E-05	0,55	1,00E+00	-4,40	0,000142717
32	4,62	8,08E-05	0,05	1,00E+00	-4,18	3,00E-04
33	6,05	1,25E-06	-0,47	1,00E+00	-5,86	7,29E-07
34	6,28	7,06E-07	-2,91	2,23E-02	-9,30	6,48E-12
35	5,75	2,78E-06	-4,37	5,89E-04	-9,55	2,90E-12
36	5,83	2,25E-06	-4,34	6,22E-04	-10,40	3,65E-13
37	6,55	3,79E-07	-1,17	7,87E-01	-7,68	8,55E-10
38	5,90	1,87E-06	-1,65	3,47E-01	-7,75	6,68E-10
39	6,78	2,25E-07	-4,15	9,08E-04	-10,97	1,35E-13
40	5,97	1,52E-06	-4,03	1,29E-03	-9,02	1,31E-11
41	5,27	1,19E-05	-3,57	3,94E-03	-8,92	1,72E-11
42	4,15	3,46E-04	-3,69	2,21E-03	-8,47	6,27E-11
43	5,00	2,59E-05	-4,30	6,53E-04	-9,83	1,42E-12

44	5,26	1,21E-05	-3,18	1,19E-02	-7,50	1,68E-09
45	5,74	2,83E-06	-3,40	7,24E-03	-9,06	1,21E-11
46	7,21	6,82E-08	-1,59	3,87E-01	-8,13	1,87E-10
47	6,92	1,84E-07	-2,41	7,31E-02	-8,70	3,13E-11
48	6,61	3,40E-07	-2,58	4,98E-02	-8,19	1,51E-10
49	5,29	1,14E-05	-3,07	1,59E-02	-7,97	3,17E-10
50	4,92	3,31E-05	-2,49	6,19E-02	-7,50	1,66E-09
51	4,85	3,93E-05	-2,70	3,71E-02	-7,96	3,35E-10
52	5,04	2,32E-05	-2,50	6,10E-02	-6,59	4,80E-08
53	4,78	5,01E-05	-3,01	1,87E-02	-7,46	1,93E-09
54	7,25	6,82E-08	-1,53	4,22E-01	-8,40	7,71E-11
55	7,24	6,82E-08	-1,66	3,42E-01	-7,75	6,80E-10
56	6,42	5,06E-07	-2,38	7,66E-02	-7,46	1,93E-09
57	4,26	2,45E-04	-1,42	5,20E-01	-5,81	8,72E-07
58	4,48	1,23E-04	-1,41	5,26E-01	-6,32	1,28E-07
59	4,68	6,73E-05	-2,14	1,32E-01	-6,37	1,08E-07
60	5,82	2,26E-06	-1,87	2,33E-01	-6,49	6,89E-08
61	6,69	2,76E-07	-0,51	1,00E+00	-6,78	2,36E-08
62	6,32	6,55E-07	-2,07	1,54E-01	-8,18	1,60E-10
63	5,05	2,24E-05	-1,73	3,03E-01	-6,03	3,86E-07
64	5,10	1,94E-05	-1,58	3,88E-01	-6,00	4,29E-07
65	4,75	5,35E-05	-1,40	5,36E-01	-5,97	4,80E-07
66	4,38	1,69E-04	-1,71	3,11E-01	-6,10	3,05E-07
67	7,54	3,13E-08	-1,77	2,82E-01	-8,02	2,73E-10
68	7,20	6,82E-08	-0,33	1,00E+00	-7,26	3,93E-09
69	6,13	9,35E-07	0,17	1,00E+00	-5,25	6,76E-06
70	6,36	5,80E-07	-1,17	7,87E-01	-5,67	1,43E-06
71	5,34	9,69E-06	-2,01	1,73E-01	-6,69	3,31E-08
72	4,60	8,55E-05	-2,38	7,66E-02	-6,62	4,25E-08
73	6,28	7,06E-07	-2,96	1,99E-02	-6,85	1,86E-08
74	5,61	4,21E-06	-2,85	2,60E-02	-6,56	5,24E-08
75	4,28	2,29E-04	-3,23	1,04E-02	-7,37	2,64E-09
76	4,29	2,22E-04	-2,42	7,10E-02	-7,03	9,22E-09
77	5,51	5,72E-06	-2,43	6,93E-02	-7,80	5,67E-10
78	5,71	3,05E-06	-1,86	2,34E-01	-7,44	2,00E-09
79	5,83	2,25E-06	-3,08	1,59E-02	-8,75	2,67E-11
80	5,47	6,35E-06	-2,90	2,34E-02	-9,69	1,99E-12
81	4,87	3,82E-05	-4,76	2,29E-04	-7,81	5,54E-10
82	4,26	2,47E-04	-4,63	3,18E-04	-7,82	5,36E-10
83	3,79	1,01E-03	-4,02	1,31E-03	-7,64	9,82E-10
84	3,86	8,65E-04	-3,88	1,87E-03	-8,35	9,15E-11
85	4,76	5,15E-05	-3,28	8,73E-03	-9,29	6,62E-12
86	5,97	1,52E-06	-3,11	1,48E-02	-9,15	9,34E-12
87	6,41	5,19E-07	-2,94	2,09E-02	-9,39	5,02E-12
88	5,88	1,95E-06	-4,74	2,35E-04	-9,61	2,46E-12
89	6,31	6,55E-07	-3,65	3,94E-03	-7,37	2,62E-09
90	5,39	8,02E-06	-5,26	8,32E-05	-8,15	1,73E-10
91	4,42	1,50E-04	-5,12	9,87E-05	-7,96	3,35E-10
92	4,05	4,82E-04	-5,63	7,76E-05	-8,31	1,04E-10

93	4,53	1,08E-04	-5,09	9,87E-05	-9,66	2,12E-12
94	4,87	3,78E-05	-4,21	8,30E-04	-9,70	1,99E-12
95	6,23	7,28E-07	-2,16	1,25E-01	-8,95	1,61E-11
96	6,57	3,75E-07	-2,95	2,09E-02	-8,27	1,15E-10
97	6,46	4,84E-07	-3,17	1,19E-02	-8,74	2,70E-11
98	4,73	5,70E-05	-4,98	1,31E-04	-8,27	1,18E-10
99	5,44	6,89E-06	-6,65	6,63E-06	-10,05	7,45E-13
100	4,86	3,84E-05	-5,29	8,32E-05	-8,65	3,62E-11
101	3,77	1,01E-03	-6,13	2,44E-05	-8,78	2,51E-11
102	3,86	8,65E-04	-5,76	6,83E-05	-7,83	5,14E-10
103	5,76	2,68E-06	-3,95	1,58E-03	-9,71	1,99E-12
104	5,81	2,29E-06	-2,67	4,09E-02	-9,70	1,99E-12
105	4,51	1,13E-04	-5,44	8,32E-05	-8,51	5,67E-11
106	5,09	1,99E-05	-5,25	8,32E-05	-8,45	6,65E-11
107	4,05	4,81E-04	-5,18	8,66E-05	-7,34	2,93E-09
108	5,54	5,28E-06	-5,02	1,14E-04	-8,90	1,86E-11
109	5,80	2,39E-06	-2,95	2,09E-02	-8,01	2,82E-10
110	5,47	6,35E-06	-2,29	9,22E-02	-7,84	5,12E-10
111	6,79	2,25E-07	-2,89	2,34E-02	-8,83	2,18E-11
112	6,05	1,25E-06	-3,54	3,94E-03	-8,61	4,03E-11
113	6,85	2,06E-07	-4,29	6,59E-04	-8,42	7,33E-11
114	5,48	6,27E-06	-4,46	4,93E-04	-8,65	3,62E-11
115	6,49	4,43E-07	-3,42	5,74E-03	-8,84	2,10E-11
116	6,17	8,31E-07	-4,07	1,16E-03	-9,16	9,02E-12
117	6,67	2,85E-07	-3,01	1,87E-02	-8,66	3,61E-11
118	6,88	2,02E-07	-2,85	2,60E-02	-8,56	4,71E-11
119	6,49	4,43E-07	-2,48	6,28E-02	-8,10	2,01E-10
120	6,44	4,99E-07	-3,49	5,74E-03	-9,56	2,84E-12
121	4,84	4,10E-05	-3,93	1,63E-03	-8,35	9,07E-11
122	4,20	3,01E-04	-5,28	8,32E-05	-10,47	3,65E-13
123	5,99	1,47E-06	-3,05	1,59E-02	-7,84	5,12E-10
124	6,77	2,25E-07	-3,53	3,94E-03	-8,41	7,33E-11
125	7,23	6,82E-08	-4,04	1,24E-03	-8,88	1,90E-11
126	6,43	5,06E-07	-4,62	3,19E-04	-8,97	1,49E-11
127	6,24	7,16E-07	-3,83	2,14E-03	-8,89	1,86E-11
128	5,45	6,65E-06	-4,22	8,24E-04	-9,35	5,59E-12
129	5,05	2,23E-05	-2,81	2,86E-02	-8,40	7,71E-11
130	5,50	5,88E-06	-2,71	3,71E-02	-8,80	2,39E-11
131	5,47	6,35E-06	-4,12	1,00E-03	-9,88	1,19E-12
132	4,86	3,84E-05	-4,52	4,43E-04	-8,83	2,19E-11
133	6,72	2,62E-07	-2,80	2,96E-02	-7,83	5,14E-10
134	8,01	9,51E-09	-3,99	1,41E-03	-9,13	9,79E-12
135	6,24	7,16E-07	-4,41	5,27E-04	-7,91	3,89E-10
136	6,19	8,08E-07	-5,23	8,43E-05	-8,93	1,69E-11
137	6,33	6,55E-07	-5,09	9,87E-05	-8,45	6,65E-11
138	5,41	7,55E-06	-4,93	1,40E-04	-8,74	2,70E-11
139	5,88	1,95E-06	-3,95	1,58E-03	-8,31	1,04E-10
140	5,74	2,83E-06	-3,62	3,94E-03	-8,82	2,20E-11
141	4,65	7,35E-05	-4,18	8,53E-04	-8,53	5,20E-11

142	6,15	8,80E-07	-2,72	3,62E-02	-6,94	1,29E-08
143	6,57	3,75E-07	-3,42	5,74E-03	-8,24	1,29E-10
144	6,00	1,42E-06	-5,36	8,32E-05	-9,28	6,80E-12
145	6,37	5,76E-07	-5,42	8,32E-05	-9,04	1,27E-11
146	5,92	1,73E-06	-4,45	5,08E-04	-8,42	7,33E-11
147	5,18	1,54E-05	-3,90	1,81E-03	-8,44	7,01E-11
148	5,14	1,77E-05	-3,59	3,94E-03	-8,16	1,73E-10
149	4,77	5,05E-05	-2,79	2,96E-02	-6,64	4,04E-08
150	6,25	7,16E-07	-2,14	1,31E-01	-7,31	3,22E-09
151	5,87	2,01E-06	-4,11	1,04E-03	-8,11	1,99E-10
152	6,18	8,08E-07	-5,32	8,32E-05	-8,64	3,63E-11
153	5,02	2,49E-05	-5,44	8,32E-05	-8,50	5,84E-11
154	4,21	2,86E-04	-4,51	4,43E-04	-8,64	3,63E-11
155	4,52	1,09E-04	-4,08	1,11E-03	-7,75	6,80E-10
156	5,13	1,80E-05	-3,10	1,48E-02	-7,48	1,76E-09
157	5,72	2,92E-06	-3,54	3,94E-03	-7,66	9,36E-10
158	6,78	2,25E-07	-3,68	3,94E-03	-8,03	2,64E-10
159	6,21	7,55E-07	-4,19	8,53E-04	-7,80	5,63E-10
160	4,86	3,84E-05	-4,37	5,90E-04	-7,66	9,26E-10
161	4,94	3,08E-05	-3,39	7,24E-03	-6,90	1,50E-08
162	5,23	1,31E-05	-2,43	6,93E-02	-6,54	5,77E-08
163	4,67	6,87E-05	-2,35	8,06E-02	-6,58	4,87E-08
164	5,22	1,36E-05	-3,66	3,94E-03	-7,80	5,67E-10
165	4,80	4,68E-05	-4,66	2,89E-04	-9,01	1,36E-11
166	4,55	9,99E-05	-4,94	1,38E-04	-9,09	1,10E-11
167	5,66	3,53E-06	-3,57	3,94E-03	-8,26	1,18E-10
168	5,94	1,66E-06	-2,98	1,99E-02	-7,30	3,32E-09
169	5,52	5,65E-06	-4,26	7,25E-04	-7,98	3,17E-10
170	4,87	3,78E-05	-3,75	2,21E-03	-6,83	2,01E-08
171	5,08	2,07E-05	-3,10	1,48E-02	-7,04	9,13E-09
172	5,06	2,22E-05	-1,89	2,26E-01	-5,80	8,99E-07
173	4,43	1,45E-04	-3,07	1,59E-02	-6,78	2,38E-08
174	3,96	6,37E-04	-3,35	7,24E-03	-6,67	3,62E-08
175	4,15	3,47E-04	-4,42	5,27E-04	-8,57	4,58E-11
176	4,56	9,76E-05	-4,50	4,57E-04	-8,32	9,86E-11
177	4,75	5,35E-05	-4,34	6,25E-04	-10,01	8,01E-13
178	5,84	2,17E-06	-2,65	4,20E-02	-7,61	1,09E-09
179	4,57	9,41E-05	-1,60	3,76E-01	-5,76	1,06E-06
180	5,27	1,19E-05	-3,27	8,73E-03	-7,44	2,03E-09
181	4,95	3,05E-05	-3,38	7,24E-03	-8,29	1,08E-10
182	4,94	3,08E-05	-4,18	8,53E-04	-9,01	1,35E-11
183	4,95	3,06E-05	-4,89	1,50E-04	-9,67	2,12E-12
184	4,37	1,71E-04	-5,59	7,76E-05	-11,39	5,07E-14
185	6,01	1,40E-06	-2,44	6,93E-02	-7,63	1,01E-09
186	6,25	7,16E-07	-2,72	3,62E-02	-8,14	1,79E-10
187	5,16	1,61E-05	-3,09	1,48E-02	-8,13	1,86E-10
188	5,12	1,86E-05	-4,36	5,90E-04	-9,23	7,55E-12
189	4,50	1,15E-04	-4,95	1,37E-04	-10,09	6,63E-13
190	4,48	1,24E-04	-4,89	1,50E-04	-10,14	5,94E-13

191	8,05	9,51E-09	-2,39	7,63E-02	-8,86	2,03E-11
192	7,93	9,51E-09	-3,28	8,73E-03	-9,07	1,15E-11
193	5,94	1,66E-06	-3,02	1,74E-02	-9,17	9,02E-12
194	4,52	1,11E-04	-5,04	1,11E-04	-8,63	3,77E-11
195	4,48	1,25E-04	-4,42	5,27E-04	-9,34	5,76E-12
196	4,76	5,15E-05	-4,30	6,53E-04	-10,70	2,85E-13
197	7,95	9,51E-09	-2,43	7,02E-02	-8,45	6,65E-11
198	7,97	9,51E-09	-0,06	1,00E+00	-7,93	3,64E-10
199	5,04	2,32E-05	-5,26	8,32E-05	-10,40	3,65E-13

Supplementary Table 7 Statistical values of post-hoc t-tests comparing the aperiodic exponent distribution according to the task period at the cortex level.

	post-pre		post-rest		rest-pre	
ROI	t	p	t	p	t	p
1	6,38	1,00E-07	-3,41	1,05E-02	-5,90	7,49E-07
2	7,10	7,19E-09	-5,88	3,09E-06	-9,00	7,98E-12
3	13,19	9,44E-18	-3,22	1,36E-02	-9,52	1,17E-12
4	11,99	2,47E-16	-3,33	1,21E-02	-9,01	7,91E-12
5	8,63	2,31E-11	-6,09	1,95E-06	-11,19	5,53E-15
6	11,19	3,51E-15	-6,02	2,14E-06	-10,16	1,61E-13
7	7,81	5,10E-10	-0,46	1,00E+00	-3,55	2,03E-03
8	9,06	5,03E-12	-0,07	1,00E+00	-4,12	3,83E-04
9	11,94	2,85E-16	-1,57	5,34E-01	-9,86	4,51E-13
10	15,36	4,01E-20	0,33	1,00E+00	-6,57	6,67E-08
11	6,72	2,90E-08	-2,66	5,37E-02	-7,14	8,11E-09
12	7,36	2,66E-09	-3,45	8,16E-03	-8,00	3,10E-10
13	7,39	2,48E-09	-1,34	7,55E-01	-4,44	1,29E-04
14	8,13	1,49E-10	-1,31	7,84E-01	-5,28	7,18E-06
15	10,22	9,08E-14	-3,21	1,36E-02	-6,64	5,28E-08
16	10,19	9,95E-14	-4,14	1,02E-03	-8,46	5,48E-11
17	8,84	1,09E-11	-2,65	5,41E-02	-6,00	5,27E-07
18	10,13	1,19E-13	-4,54	2,69E-04	-9,56	1,05E-12
19	12,54	5,44E-17	-3,00	2,55E-02	-8,85	1,38E-11
20	12,89	1,79E-17	-4,80	1,26E-04	-12,26	2,84E-16
21	10,25	8,89E-14	-1,11	1,00E+00	-6,68	4,53E-08
22	8,47	4,16E-11	-0,91	1,00E+00	-7,06	1,08E-08
23	10,50	3,87E-14	-1,28	7,97E-01	-5,15	1,11E-05
24	6,56	5,28E-08	-3,32	1,21E-02	-6,01	5,22E-07
25	12,48	6,17E-17	-0,98	1,00E+00	-9,71	6,65E-13
26	13,03	1,32E-17	-1,98	2,65E-01	-9,88	4,29E-13
27	10,11	1,22E-13	0,10	1,00E+00	-3,91	7,60E-04
28	8,78	1,38E-11	-1,63	5,17E-01	-5,52	3,04E-06
29	12,37	7,56E-17	-1,43	6,62E-01	-9,62	8,57E-13
30	12,44	6,20E-17	-1,43	6,62E-01	-9,70	6,75E-13
31	12,06	2,06E-16	-4,83	1,17E-04	-10,32	9,86E-14

32	10,03	1,51E-13	-5,64	7,30E-06	-11,01	9,86E-15
33	7,51	1,59E-09	0,25	1,00E+00	-5,97	5,86E-07
34	9,35	1,73E-12	-0,88	1,00E+00	-6,20	2,62E-07
35	9,88	2,43E-13	-1,89	3,15E-01	-6,18	2,75E-07
36	11,80	4,36E-16	-2,76	4,47E-02	-8,76	1,88E-11
37	5,04	1,47E-05	-6,63	6,32E-07	-9,77	5,83E-13
38	6,53	5,77E-08	-8,21	4,59E-09	-14,56	5,07E-19
39	10,22	9,08E-14	-6,07	1,95E-06	-14,02	1,43E-18
40	10,04	1,48E-13	-2,75	4,53E-02	-11,39	3,35E-15
41	6,68	3,31E-08	-7,58	2,13E-08	-12,77	6,81E-17
42	6,80	2,18E-08	-8,08	4,59E-09	-16,07	1,03E-20
43	8,14	1,47E-10	0,53	1,00E+00	-3,04	1,10E-02
44	9,42	1,35E-12	-0,63	1,00E+00	-4,66	6,32E-05
45	14,73	1,49E-19	-3,06	2,19E-02	-8,48	5,40E-11
46	12,91	1,79E-17	-6,12	1,93E-06	-12,26	2,84E-16
47	10,91	8,98E-15	-1,56	5,34E-01	-6,63	5,34E-08
48	13,78	1,78E-18	-2,80	4,20E-02	-8,22	1,34E-10
49	9,96	1,90E-13	-4,76	1,38E-04	-10,87	1,57E-14
50	13,91	1,36E-18	-5,94	2,67E-06	-11,76	1,12E-15
51	10,45	4,41E-14	1,28	7,97E-01	-4,98	2,09E-05
52	10,07	1,36E-13	0,61	1,00E+00	-4,82	3,61E-05
53	13,48	4,08E-18	-1,24	8,31E-01	-10,75	2,26E-14
54	15,25	4,01E-20	-1,61	5,17E-01	-11,81	1,03E-15
55	8,88	9,69E-12	-4,62	2,11E-04	-12,09	4,49E-16
56	6,36	1,06E-07	-6,44	7,40E-07	-14,03	1,43E-18
57	15,99	1,31E-20	-6,45	7,40E-07	-11,64	1,56E-15
58	14,32	4,32E-19	-5,09	4,88E-05	-10,16	1,61E-13
59	6,85	1,85E-08	0,37	1,00E+00	-4,50	1,06E-04
60	6,67	3,42E-08	-0,58	1,00E+00	-5,45	3,87E-06
61	11,69	6,15E-16	-2,67	5,33E-02	-8,30	1,01E-10
62	7,21	4,70E-09	-5,33	2,13E-05	-10,47	5,91E-14
63	8,67	2,05E-11	-2,88	3,30E-02	-6,53	7,58E-08
64	10,09	1,31E-13	-6,40	7,55E-07	-11,19	5,53E-15
65	12,45	6,20E-17	-1,71	4,52E-01	-11,22	5,53E-15
66	13,06	1,29E-17	-1,61	5,17E-01	-9,78	5,66E-13
67	10,23	9,08E-14	-4,01	1,49E-03	-7,87	4,91E-10
68	5,14	1,01E-05	-6,51	7,40E-07	-9,73	6,30E-13

Supplementary Table 8 Statistical values of post-hoc t-tests comparing the aperiodic offset distribution according to the task period at the cortex level.

	post-pre		post-rest		rest-pre	
	t	p	t	p	t	p
1	12,19	6,12E-17	2,35	1,01E-01	-2,12	1,71E-01
2	10,83	5,77E-15	1,27	7,87E-01	-3,09	1,65E-02
3	12,57	1,76E-17	5,50	8,94E-06	-3,32	9,44E-03

4	11,97	1,24E-16	4,17	6,50E-04	-3,56	4,39E-03
5	17,50	8,43E-23	1,47	5,81E-01	-4,77	1,23E-04
6	13,35	1,56E-18	1,08	1,00E+00	-4,90	8,23E-05
7	8,86	7,72E-12	6,57	5,20E-07	1,51	5,78E-01
8	9,00	4,60E-12	6,88	1,94E-07	1,29	8,12E-01
9	13,75	4,62E-19	5,50	8,94E-06	-2,07	1,87E-01
10	14,75	3,71E-20	7,58	6,41E-08	0,66	1,00E+00
11	14,74	3,71E-20	1,25	7,87E-01	-6,86	1,47E-07
12	15,25	1,20E-20	0,42	1,00E+00	-7,20	4,69E-08
13	11,76	2,48E-16	4,28	4,97E-04	-0,75	1,00E+00
14	8,67	1,52E-11	5,13	3,00E-05	0,69	1,00E+00
15	9,23	1,99E-12	3,89	1,58E-03	-0,79	1,00E+00
16	8,49	3,02E-11	2,63	5,22E-02	-2,24	1,39E-01
17	12,11	7,82E-17	3,57	3,78E-03	-2,23	1,39E-01
18	12,81	8,33E-18	2,69	4,64E-02	-3,59	4,39E-03
19	13,84	3,88E-19	5,65	6,08E-06	-1,21	9,02E-01
20	14,01	2,52E-19	4,24	5,62E-04	-2,46	8,50E-02
21	9,28	1,68E-12	6,08	1,85E-06	-0,79	1,00E+00
22	7,60	9,34E-10	4,46	2,74E-04	-1,19	9,16E-01
23	11,86	1,78E-16	4,47	2,74E-04	0,01	1,00E+00
24	13,94	2,96E-19	2,85	3,14E-02	-2,31	1,23E-01
25	16,10	1,03E-21	6,19	1,35E-06	-3,05	1,79E-02
26	14,42	9,24E-20	5,69	5,65E-06	-2,12	1,71E-01
27	10,35	3,34E-14	5,30	1,73E-05	0,00	1,00E+00
28	10,84	5,75E-15	4,06	9,33E-04	-1,31	7,92E-01
29	14,11	2,04E-19	5,73	5,49E-06	-3,83	2,36E-03
30	13,78	4,35E-19	5,82	4,66E-06	-3,49	6,18E-03
31	16,73	2,99E-22	2,76	3,91E-02	-3,87	2,20E-03
32	12,91	6,12E-18	1,85	2,89E-01	-4,63	1,96E-04
33	6,17	2,15E-07	5,73	5,49E-06	1,17	9,29E-01
34	7,02	8,38E-09	5,20	2,39E-05	0,32	1,00E+00
35	10,83	5,77E-15	5,60	6,95E-06	-1,14	9,53E-01
36	12,57	1,76E-17	4,20	6,12E-04	-2,94	2,44E-02
37	18,83	4,51E-24	-0,14	1,00E+00	-6,66	2,81E-07
38	16,24	8,50E-22	-1,34	7,08E-01	-8,24	1,65E-09
39	16,39	6,63E-22	0,70	1,00E+00	-5,83	5,39E-06
40	14,12	2,04E-19	3,08	1,61E-02	-5,34	2,35E-05
41	13,03	4,39E-18	-0,67	1,00E+00	-6,61	2,97E-07
42	12,88	6,57E-18	-0,57	1,00E+00	-8,56	1,45E-09
43	9,29	1,67E-12	6,21	1,35E-06	1,36	7,45E-01
44	10,94	4,19E-15	4,83	8,16E-05	-0,21	1,00E+00
45	15,00	2,18E-20	2,03	2,04E-01	-3,27	9,44E-03
46	10,85	5,75E-15	0,84	1,00E+00	-5,24	3,00E-05
47	9,01	4,43E-12	1,75	3,50E-01	-3,63	4,39E-03
48	12,94	5,84E-18	0,11	1,00E+00	-5,49	1,50E-05
49	11,56	4,92E-16	1,06	1,00E+00	-4,29	5,78E-04
50	13,99	2,62E-19	0,88	1,00E+00	-5,48	1,50E-05
51	9,05	3,94E-12	6,23	1,35E-06	0,77	1,00E+00
52	8,84	8,26E-12	7,21	1,34E-07	0,26	1,00E+00

53	14,87	2,97E-20	6,90	1,94E-07	-3,88	2,20E-03
54	16,76	2,99E-22	6,41	8,31E-07	-5,09	4,88E-05
55	14,17	1,84E-19	3,14	1,47E-02	-7,36	3,03E-08
56	16,21	8,50E-22	1,26	7,87E-01	-8,30	1,65E-09
57	14,05	2,31E-19	1,48	5,80E-01	-7,35	3,03E-08
58	13,66	5,98E-19	2,12	1,70E-01	-5,52	1,50E-05
59	7,26	3,33E-09	6,90	1,94E-07	1,13	9,62E-01
60	6,38	9,62E-08	5,49	8,94E-06	0,37	1,00E+00
61	16,86	2,99E-22	3,70	1,94E-03	-3,32	9,44E-03
62	15,08	1,85E-20	1,96	2,32E-01	-5,04	5,59E-05
63	13,83	3,88E-19	2,38	9,58E-02	-2,17	1,59E-01
64	11,10	2,45E-15	0,60	1,00E+00	-4,91	8,23E-05
65	14,17	1,84E-19	4,94	5,60E-05	-4,40	4,23E-04
66	13,45	1,17E-18	5,69	5,65E-06	-3,52	6,18E-03
67	14,73	3,71E-20	2,64	5,18E-02	-3,57	4,39E-03
68	12,23	5,46E-17	0,57	1,00E+00	-5,30	2,52E-05

Supplementary Table 9 P-values (FDR-corrected) and rho coefficients of Spearman correlations between aperiodic activity and behavioral data or clinical characteristics at the scalp level (A) and source level (B).

A - SCALP

Spearman correlation FDR-corrected		Aperiodic activity			
		HC (n=30)		PD (n=29)	
		Exponent	Offset	Exponent	Offset
**Behavioral data**	Accuracy	p=0.86,r=-0.38	p=0.86,r=-0.43	p=0.28,r=0.13	p=0.86,r=0.07
	First bin	p=0.86,r=-0.34	p=0.76,r=-0.40	p=0.76,r=0.22	p=0.76,r=0.17
	Last slope	p=0.28,r=-0.16	p=0.40,r=-0.07	p=0.76,r=0.14	p=0.76,r=0.31
**Clinical characteristics**	Age	p=0.86,r=0.16	p=0.86,r=0.14	p=0.28,r=0.09	p=0.86,r=0.04
	MoCA	p=0.28,r=-0.36	p=0.86,r=-0.20	p=0.28,r=-0.06	p=0.60,r=-0.05
	UPDRS – on	-	-	p=0.28,r=-0.27	p=0.76,r=-0.04
	UPDRS – off	-	-	p=0.86,r=-0.04	p=0.76,r=-0.09
	LEDD	-	-	p=0.76,r=-0.09	p=0.76,r=-0.17
	Duration	-	-	p=0.76,r=-0.36	p=0.86,r=-0.18

B - SOURCE

Spearman correlation FDR-corrected		Aperiodic activity			
		HC (n=30)		PD (n=29)	
		Exponent	Offset	Exponent	Offset
**Behavioral data**	Accuracy	p=0.98,r=-0.06	p=0.98,r=-0.46	p=0.98,r=8.9^{-3}	p=0.98,r=-0.01
	First bin	p=0.98,r=6.2^{-3}	p=0.98,r=-0.39	p=0.98,r=0.13	p=0.75,r=0.14
	Last slope	p=0.91,r=0.21	p=0.98,r=0.26	p=0.80,r=0.24	p=0.98,r=0.10
**Clinical characteristics**	Age	p=0.98,r=0.02	p=0.98,r=0.18	p=0.98,r=0.15	p=0.98,r=0.02
	MoCA	p=0.98,r=5.4^{-3}	p=0.98,r=-0.11	p=0.98,r=-0.06	p=0.98,r=-0.07
	UPDRS – on	-	-	p=0.70,r=-0.32	p=0.14,r=-0.06
	UPDRS – off	-	-	p=0.98,r=-0.01	p=0.37,r=-0.09
	LEDD	-	-	p=0.98,r=-8.9^{-3}	p=0.75,r=0.28
	Duration	-	-	p=0.14,r=-0.46	p=0.98,r=-0.11

Supplementary Table 10 Regions of interest where aperiodic parameters are significantly different according to the effect tested (two-way repeated measures ANOVAs with FDR correction).

Tested effect	Aperiodic parameter	Significant ROI
**Group – post stimulus part**	Exponent	-
	Offset	cuneus L and R<br>entorhinal L<br>fusiform L<br>fusiform R<br>inferior parietal L<br>inferior temporal L<br>lateral occipital L and R<br>lingual L<br>middle temporal L and R<br>parahippocampal L<br>pericalcarine L<br>postcentral L<br>precuneus L and R<br>superior parietal L<br>temporal pole L and R
**Congruence – post stimulus part**	Exponent	paracentral L and R<br>posterior cingulate L and R<br>precentral L and R
	Offset	paracentral L<br>posterior cingulate R
**Group – all parts**	Exponent	-
	Offset	bankssts L and R<br>cuneus L and R<br>entorhinal L<br>fusiform L and R<br>inferior parietal L and R<br>inferior temporal L and R<br>isthmus cingulate L and R<br>lateral occipital L and R<br>lateral orbitofrontal L<br>middle temporal L and R<br>paracentral L<br>parahippocampal L and R<br>pericalcarine L<br>postcentral L<br>posterior cingulate L and R<br>precuneus L and R<br>superior parietal L<br>superior temporal L and R<br>temporal pole L and R<br>transverse temporal L
**Task period**	Exponent	All
	Offset	All

L: left; R: right.

Supplementary Table 11 : Statistical values of ANOVAs assessing the onset side effect within the PD patients population on aperiodic parameters at both scalp and cortex level.

	OFFSET		EXPONENT			OFFSET		EXPONENT	
ELECTRODE	F_group	p_group	F_group	p_group	ROI	F_group	p_group	F_group	p_group
1	0,41	0,86	1,09	0,86	1	1,55	0,63	1,06	0,67
2	0,31	0,81	0,41	0,91	2	0,47	0,63	0,34	0,67
3	0,18	1,00	0,26	0,91	3	0,03	0,46	0,03	0,73
4	0,01	0,84	0,01	0,86	4	0,00	0,98	0,07	0,86
5	1,06	0,65	1,23	0,91	5	2,93	0,42	1,67	0,91
6	1,74	0,65	1,99	0,71	6	3,64	0,42	1,38	0,63
7	0,34	0,94	0,28	1,00	7	0,88	0,75	0,38	0,89
8	0,69	0,65	0,54	0,71	8	0,44	0,07	0,22	0,24
9	0,09	0,94	0,04	0,97	9	0,15	0,76	0,02	0,91
10	0,10	1,00	0,28	0,95	10	1,18	0,95	0,81	0,76
11	1,78	0,88	0,93	0,94	11	0,05	0,88	0,01	0,91
12	0,60	0,77	0,00	0,71	12	0,03	0,98	0,11	0,91
13	0,96	0,79	0,65	0,91	13	0,13	0,65	0,00	0,74
14	1,64	0,99	1,98	0,95	14	0,12	0,98	0,00	0,91
15	0,41	1,00	0,67	0,94	15	1,84	0,96	2,23	0,91
16	0,30	0,65	0,50	0,85	16	1,91	0,64	2,27	0,85
17	0,79	0,71	0,27	0,66	17	0,18	0,76	0,00	0,85
18	0,13	0,99	1,09	0,95	18	0,26	0,73	0,00	0,74
19	0,02	0,94	0,00	0,91	19	1,30	0,60	0,01	0,74
20	0,08	0,93	0,17	0,97	20	2,53	0,98	0,59	0,74
21	0,12	1,00	0,05	0,94	21	0,11	0,95	0,18	0,95
22	0,13	0,94	0,26	0,94	22	0,01	0,98	0,01	0,85
23	0,71	1,00	0,93	0,95	23	0,21	0,90	0,10	0,91
24	1,86	0,91	1,37	0,94	24	1,47	0,98	0,44	0,86
25	0,06	0,86	0,02	0,99	25	0,55	0,63	0,03	0,74
26	0,01	0,84	0,01	0,94	26	1,81	0,63	0,09	0,49
27	0,01	1,00	0,16	0,95	27	0,43	0,18	1,12	0,20
28	0,18	0,97	0,78	0,98	28	0,05	0,19	0,05	0,07
29	0,26	0,79	0,23	0,85	29	0,01	0,32	0,17	0,36
30	0,06	0,87	0,01	0,95	30	0,91	0,64	0,09	0,63
31	0,18	0,65	0,28	0,79	31	3,56	0,18	0,78	0,31
32	0,05	0,86	0,00	0,96	32	0,04	0,98	0,01	0,95

**33**	0,04	1,00	0,00	0,95	**33**	0,00	0,37	0,46	0,31
**34**	0,52	0,86	0,05	0,94	**34**	0,00	0,50	0,51	0,39
**35**	0,05	0,80	0,00	0,94	**35**	0,28	0,98	0,10	0,99
**36**	0,09	0,89	0,00	0,91	**36**	0,01	0,42	0,01	0,39
**37**	0,04	0,97	0,04	0,91	**37**	0,73	0,75	0,19	0,72
**38**	0,74	1,00	0,45	0,98	**38**	2,61	0,58	0,09	0,60
**39**	0,36	0,87	0,46	0,94	**39**	0,67	0,46	0,19	0,67
**40**	0,31	1,00	0,22	0,95	**40**	1,63	0,41	0,02	0,60
**41**	0,09	0,71	0,30	0,83	**41**	1,38	0,74	0,05	0,91
**42**	0,04	1,00	0,06	0,86	**42**	0,93	0,75	0,02	0,82
**43**	0,81	0,93	0,03	0,91	**43**	0,02	0,06	0,21	0,07
**44**	0,10	0,86	0,01	0,83	**44**	0,11	0,82	0,10	0,67
**45**	0,06	1,00	0,58	0,99	**45**	0,56	0,98	0,00	0,91
**46**	0,64	0,93	0,37	0,91	**46**	1,09	0,75	0,70	0,68
**47**	0,33	0,94	2,04	0,91	**47**	0,47	0,75	0,41	0,53
**48**	0,29	1,00	0,63	0,99	**48**	0,66	0,63	0,56	0,53
**49**	0,42	0,86	1,55	0,71	**49**	1,18	0,64	0,08	0,63
**50**	0,33	0,86	0,33	0,91	**50**	2,39	0,49	1,50	0,24
**51**	0,04	0,94	0,40	0,91	**51**	0,28	0,99	0,96	0,91
**52**	0,00	0,90	0,30	0,91	**52**	0,48	0,82	0,30	0,66
**53**	1,25	1,00	3,21	0,95	**53**	0,13	0,64	0,00	0,72
**54**	0,92	0,93	0,00	0,91	**54**	0,00	0,88	0,09	0,91
**55**	0,34	0,77	2,58	0,85	**55**	0,31	0,63	0,42	0,45
**56**	0,20	0,86	1,59	0,98	**56**	0,46	0,75	0,24	0,53
**57**	0,04	0,97	0,67	0,86	**57**	0,84	0,63	1,05	0,73
**58**	1,19	0,86	0,01	0,95	**58**	0,36	0,46	0,52	0,74
**59**	0,11	0,89	0,11	0,95	**59**	1,66	0,81	1,41	0,64
**60**	0,02	0,65	0,32	0,71	**60**	0,28	0,88	0,85	0,86
**61**	0,35	0,78	0,09	0,86	**61**	0,61	0,33	0,01	0,39
**62**	0,61	0,86	1,53	0,91	**62**	0,14	0,07	0,05	0,16
**63**	0,24	1,00	0,36	0,94	**63**	4,96	0,63	2,54	0,53
**64**	0,13	1,00	0,53	0,95	**64**	1,53	0,96	1,53	0,85
**65**	1,40	0,86	0,06	0,86	**65**	0,50	0,18	0,04	0,36
**66**	1,01	1,00	0,28	0,95	**66**	0,04	0,98	0,05	0,91
**67**	1,25	0,86	0,02	0,91	**67**	0,01	0,82	0,34	0,91
**68**	0,33	0,86	0,03	0,91	**68**	0,22	0,98	0,62	0,91
**69**	0,20	0,87	0,88	0,84					
**70**	0,58	1,00	0,06	0,95					
**71**	1,60	0,86	0,05	0,85					

**72**	0,55	0,86	0,47	0,91					
**73**	0,00	0,93	0,31	0,91					
**74**	2,71	0,88	0,41	0,91					
**75**	2,05	1,00	1,04	1,00					
**76**	0,32	0,71	0,50	0,85					
**77**	0,42	0,93	1,55	0,91					
**78**	3,11	0,94	4,19	0,95					
**79**	0,02	0,94	2,36	0,95					
**80**	2,64	0,86	4,70	0,94					
**81**	0,24	0,92	0,00	0,86					
**82**	0,03	1,00	0,00	0,91					
**83**	0,03	0,86	0,12	0,91					
**84**	0,00	1,00	0,13	0,95					
**85**	0,22	1,00	1,01	0,95					
**86**	0,00	0,65	1,04	0,71					
**87**	0,30	0,86	2,44	0,91					
**88**	0,32	0,89	4,04	0,95					
**89**	0,88	1,00	0,22	0,91					
**90**	0,05	0,90	0,25	0,95					
**91**	0,47	0,94	0,01	0,95					
**92**	0,52	0,95	0,13	0,95					
**93**	0,89	0,94	1,34	0,94					
**94**	0,51	0,99	1,72	0,91					
**95**	1,77	0,86	5,69	0,84					
**96**	0,08	0,90	2,32	0,91					
**97**	2,13	0,86	10,54	0,91					
**98**	0,00	0,99	0,00	0,95					
**99**	0,17	1,00	0,01	0,99					
**100**	0,01	0,86	0,00	0,86					
**101**	0,34	0,86	0,14	0,86					
**102**	1,56	0,65	1,52	0,71					
**103**	0,39	0,92	1,94	0,91					
**104**	0,20	0,94	2,96	0,91					
**105**	0,64	1,00	0,21	0,91					
**106**	0,04	0,94	0,04	0,95					
**107**	0,00	0,78	0,00	0,78					
**108**	0,05	0,86	0,10	0,91					

**109**	0,11	0,65	0,85	0,91					
**110**	0,26	1,00	0,08	0,94					
**111**	0,61	0,86	5,45	0,95					
**112**	0,79	0,97	0,03	0,98					
**113**	0,18	0,87	0,03	0,91					
**114**	0,35	1,00	0,39	0,95					
**115**	0,11	0,94	0,02	0,94					
**116**	0,07	0,84	0,47	0,88					
**117**	0,32	0,65	3,04	0,91					
**118**	0,67	0,86	6,04	0,95					
**119**	0,67	0,94	3,77	0,86					
**120**	0,43	0,87	4,96	0,95					
**121**	0,05	0,65	1,27	0,27					
**122**	0,10	0,86	1,46	0,86					
**123**	0,14	0,65	0,04	0,71					
**124**	0,49	0,92	0,36	0,95					
**125**	0,03	0,86	0,00	0,71					
**126**	0,89	0,94	0,01	0,97					
**127**	0,18	0,94	0,09	0,91					
**128**	0,15	0,79	0,65	0,88					
**129**	0,01	0,92	0,76	0,86					
**130**	0,99	0,71	3,35	0,35					
**131**	1,01	0,86	2,75	0,85					
**132**	0,80	0,78	2,88	0,85					
**133**	1,33	1,00	0,19	0,95					
**134**	0,58	0,87	0,00	0,86					
**135**	0,29	0,87	0,02	0,94					
**136**	0,42	0,92	0,04	0,91					
**137**	0,15	1,00	0,97	0,95					
**138**	0,54	1,00	1,10	0,91					
**139**	0,30	0,65	1,59	0,66					
**140**	2,45	0,84	4,09	0,84					
**141**	3,34	0,94	3,28	0,94					
**142**	2,46	0,84	0,38	0,83					
**143**	0,41	0,86	0,52	0,91					
**144**	0,20	0,95	0,06	0,95					
**145**	0,57	0,86	1,17	0,94					

**146**	0,69	0,86	0,56	0,86					
**147**	0,76	0,86	0,66	0,86					
**148**	5,80	0,84	6,31	0,85					
**149**	8,21	0,86	6,73	0,71					
**150**	0,01	0,86	0,11	0,86					
**151**	0,02	0,86	0,09	0,91					
**152**	0,58	0,91	0,31	0,91					
**153**	0,78	0,86	0,38	0,71					
**154**	0,58	0,65	0,31	0,71					
**155**	2,72	0,84	2,78	0,71					
**156**	2,64	1,00	4,65	0,94					
**157**	0,00	0,87	0,44	0,94					
**158**	1,42	0,94	0,57	0,91					
**159**	0,48	0,94	0,09	0,95					
**160**	0,78	1,00	0,47	0,94					
**161**	2,32	0,77	1,74	0,71					
**162**	5,35	0,84	4,36	0,83					
**163**	1,46	0,84	1,30	0,71					
**164**	4,17	0,60	2,72	0,14					
**165**	5,62	0,65	2,20	0,71					
**166**	1,22	1,00	0,83	0,95					
**167**	0,69	0,84	1,07	0,71					
**168**	0,24	1,00	0,60	0,91					
**169**	0,01	0,86	0,09	0,91					
**170**	0,12	1,00	0,20	0,95					
**171**	4,08	0,71	1,73	0,71					
**172**	2,64	0,86	1,70	0,91					
**173**	0,81	0,93	0,05	0,95					
**174**	6,75	1,00	1,07	0,98					
**175**	0,44	0,87	0,11	0,91					
**176**	1,22	0,65	0,23	0,27					
**177**	0,45	0,86	0,12	0,86					
**178**	0,31	0,90	0,41	0,94					
**179**	2,43	0,80	0,45	0,85					
**180**	0,30	0,92	0,12	0,95					
**181**	2,16	0,87	0,35	0,91					
**182**	1,48	0,86	0,19	0,91					

**183**	4,65	1,00	1,19	0,85					
**184**	2,26	0,86	0,84	0,71					
**185**	0,02	0,86	0,02	0,85					
**186**	0,63	0,97	0,21	0,94					
**187**	1,10	0,94	0,26	0,91					
**188**	0,19	0,90	0,17	0,91					
**189**	0,23	0,97	0,03	0,91					
**190**	1,91	0,86	1,11	0,91					
**191**	0,34	1,00	0,49	0,95					
**192**	0,10	0,92	0,66	0,91					
**193**	0,58	0,71	0,02	0,84					
**194**	1,14	0,94	0,80	0,91					
**195**	0,00	0,94	0,08	0,71					
**196**	0,14	0,86	0,15	0,95					
**197**	0,82	0,86	0,21	0,84					
**198**	1,07	0,65	0,31	0,71					
**199**	0,00	1,00	0,37	0,98					
